# Factors influencing methamphetamine use among Lahu hill tribe youth in Chiang Rai, Thailand: A case‒control study

**DOI:** 10.1371/journal.pone.0344775

**Published:** 2026-03-12

**Authors:** Phootawan Thinpanyawong, Tawatchai Apidechkul, Karl Peltzer, Pilasinee Wongnuch

**Affiliations:** 1 School of Health Science, Mae Fah Luang University, Chiang Rai, Thailand; 2 Regional Health Promotion Center-2 Phitsanulok, Department of Health, Ministry of Public Health, Phitsanulok, Thailand; 3 Center of Excellence for the Hill tribe Health Research, Mae Fah Luang University, Chiang Rai, Thailand; 4 Department of Health Education and Behavioral Sciences, Faculty of Public Health, Mahidol University, Bangkok, Thailand; 5 Department of Psychology, Asia University, Taichung, Taiwan; Shenzhen University, CHINA

## Abstract

**Background:**

Methamphetamine (MA) use presents a significant public health issue that strongly affects human health and social security. Marginalized populations, including youth from the Lahu tribe, which represents the second-largest hill tribe living in the remote border areas of northern Thailand, are among the most vulnerable to MA use. This study aimed to determine the factors associated with MA use among Lahu youths aged 15–24 years who lived in Chiang Rai Province, Thailand.

**Methods:**

A case‒control study was conducted to determine the factors associated with MA use among Lahu youths aged 15–24 years. The Lahu youths who used MA were considered cases of MA use, and those who never used MA were considered controls. The participants were randomly recruited from Mae Suai, Mae Chan, Mae Fah Luang, and Muang Districts, Chiang Rai Province, Thailand, the areas of Thailand most commonly populated by the Lahu people. A validated questionnaire was used to collect data, and multiple logistic regression was used to detect association factors with a statistical significance set at α = 0.05.

**Results:**

A total of 136 cases and 272 controls were included in the study. The majority of the participants were male (79.4%), 77.9% held a Thai national identification card, and 24.0% had completed primary school. After controlling for age, those females were 5.29-fold (95% CI = 2.02–13.89) more likely to use MA than males. Those with easy-to-access MA had a 2.28-fold (95% CI = 1.14–4.54) higher likelihood of MA use than those who had difficulty accessing. Those who had no schooling had a 4.29-fold (95% CI = 1.17–15.73) higher likelihood of MA use than those who had vocational and university education. Those who were unemployed had a 3.92-fold (95% CI = 1.14–13.37) higher likelihood of MA than those who were students. Those who smoked or had ever smoked had a 29.08-fold (95% CI = 11.57–73.12) greater likelihood of MA use than those who never smoked. Those who drank alcohol had a 2.53-fold (95% CI = 1.05–6.11) higher likelihood of MA use than those who did not. Those who had high knowledge scores of MA prevention and control would be more likely to use MA than those who had lower scores (AOR = 1.29; 95% CI = 1.11–1.50).

**Conclusions:**

An effective intervention and policy that focuses on females with low levels of education, unemployed, and having a history of substance use should be urgently developed and implemented to reduce MA use among Lahu youths.

## Introduction

Methamphetamine (MA) is a strongly addictive stimulant that affects the central nervous system (CNS) by releasing and inhibiting the reabsorption of serotonin, norepinephrine, and dopamine [1,2]. High levels of these neurotransmitters in synapses lead to euphoria, insomnia, increased libido, and increased energy for a longer time awake [3]. MA abuse is defined as the use of MA in any context, leading to long-term use with both physical and mental health consequences [4–6], including suicidal behaviors [7]. The long-term use of MA can lead to several chronic diseases [8]. MA not only negatively affects the health of users of MA but also affects the family’s finances and the security of people in the community [4]. People initiate MA use for various reasons, such as curiosity [9], having a positive viewpoint of MA use [10–11], and pressure from peers [11] or by family members [11]. Youths are the group most vulnerable to MA use [12].

The United Nations Office on Drugs and Crime (UNODC) reported that in 2019, the majority of amphetamine-type stimulant (ATS) substances were MA, and 271 million people (5.5%) used narcotic drugs worldwide; among these, 29 million used MA, and this number has been increasing each year [13]. The majority of users of MA use began using MA while they were young, typically around the age of 22 years [14]. Previous studies of European populations reported MA use beginning at a median of 20 years of age [15]. More than 120 tons of MA were produced in Southeast Asia in 2018 [16]. Thailand, particularly the northern region of Thailand, has been reported as one of the largest sources of MA worldwide [17]. MA is the most commonly used drug substance across Thailand [18]. Among new registered patients in substance abuse clinics, 26.5% are under 25 years of age [18,19]. High rates of cigarette use and alcohol consumption are also observed within this age group, which could ultimately lead to MA use [20–22].

Chiang Rai Province is located in northernmost Thailand, with a population of more than 1.2 million people, and has been reported as a major part of the route through which MA is imported from neighboring countries, transported to large cities in Thailand, and distributed throughout Thailand or exported to other countries [23]. More than 30% of the people living in Chiang Rai Province are members of hill tribes, each with its own culture, language, and traditional subsistence crops grown for daily survival [23]. There are six main tribes in the Chiang Rai Province: the Akha, Lahu, Hmong, Yao, Karn, and Lisu peoples. After the Akha people, the Lahu population is the second largest of these groups [20]. A large proportion of hill tribe people, including the Lahu people, live below Thailand's national poverty line ($1400 per year per family) [24,25]. Many Lahu people are not granted a Thai identification card, which is required for all public services, including health care services; the national Thai government allocates resources to only those who hold an ID card [26]. Several conditions, including the distance from their village to a school and poor family economics, also limit Lahu youths’ access to education [27]. These conditions promote MA use among the Lahu people as a means of relieving life stressors and participation in MA trade to obtain money for family members, particularly the youths.

Given their poor socioeconomic status and location along the border, Lahu youths have been reported as having one of the highest rates of MA use, with 14.8% of those aged 15–24 years using MA [25]. However, studies on the factors associated with MA use among Lahu youths are lacking. The present study aimed to determine the factors associated with MA use among Lahu youths aged 15–24 years n Chiang Rai Province, Thailand. These findings could be used to generate effective public health policies and interventions to address this particular issue.

## Methods

### Study design

A community-based case‒control study design was used for data collection to test the hypothesis that education, individual health risk behaviors, and peer relationships are associated with MA use among Lahu youths.

### Study population

Lahu youths aged 15–24 years who were living in Lahu villages in Chiang Rai Province, Thailand, comprised the study population.

### Sample size

The sample size was calculated based on information obtained from a previous report about the MA use in this population in Thailand [19]. The sample size was calculated using the standard formula for a case‒control study [28] and on the basis of the following assumptions: p1 (proportion of exposed cases based on previous study) = 0.884 [32], p2 (proportion of exposed controls based on previous study) = 0.769 [32], q1 – (1 – p1) = 0.116, q2 – (1 – p2) = 0.231, Z_1‒α_(confidence at an error of 5.0%)= 1.96, Z_1‒β_(the power of the study at 80.0%) = 0.84, r (the ratio of cases to controls) = 2, and the odds ratio (OR) from the previous study = 2.29 [19]. Using these assumptions, we calculated that a total of 136 cases and 272 controls (a control/case ratio of 2.0) were needed for the analysis.

A total of 136 cases were randomly selected from the 227 cases of MA use in the clinics between January 2022 and March 2024. No individuals refused to provide this information. However, three selected cases moved to other provinces and could not be reached for information. In addition, 276 gender-matched controls were selected from the villages.

### Study sample

The study participants were recruited from Lahu youths who lived in Mae Fah Luang, Mae Chan, Mae Suai, and Muang Districts, Chiang Rai Province. The cases included Lahu youth who currently use or have previously used MA. The inclusion criteria for cases included (i) members of the Lahu tribe aged 15–24 years, (ii) self-reported MA use, and (iii) participation in the MA treatment program as a client. The controls were Lahu youth who had never used MA, and the inclusion criteria for controls included (i) members of the Lahu tribe between 15 and 24 years old and (ii) triple-negative MA testing results based on the following: (a) never-user of MA by self-report, (b) a negative MA urine test result, and (c) a non-MA user consensus in the triple-check method. However, patients and controls with cognitive impairment and those who were unable to communicate were excluded from the study. Individuals who had a positive result in one of the three methods were excluded from the control group.

A triple-check method was used to confirm the MA use status of the control by identifying three people who knew the potential participants (a village headman, a close friend, and a family member). These individuals were asked whether the potential participant used MA or not. The responses were “yes” or “no”. If any of these persons responded “yes”, the potential participant was excluded from the control selection. The information obtained remained with the researcher without being shared with anyone. The method aimed to ensure that all selected controls had never used MA.

### Recruitment procedure

Lahu youths living in Lahu village in Chiang Rai Province who attended any of four district hospital MA clinics, namely, the Mae Fah Luang, Mae Chan, Muang, and Mae Suai district hospitals, between January 2022 and March 2024 were listed and selected randomly as cases. Once a case was selected, the village headman was contacted to obtain a list of Lahu youth aged 15–24 years who did not use MA living in the village. At the meeting with the village headman to learn where the case(s) lived, information about the project was provided. Youths aged 15–24 years who did not use MA and lived in the same village as the cases were randomly selected as controls. Controls were matched by sex to cases.

A triple-check method was applied to confirm the lack of MA use among the control. Once two controls for each case were obtained, the potential controls were asked for a voluntary urine test. The participants were asked to complete a self-administered questionnaire. Identifiable personal information was not included in the questionnaire. During the completion of the questionnaire, all the participants were informed that they were encouraged to ask any questions if they did not understand parts of the questionnaire. All parts of the questionnaire and testing were conducted in a private room after the participants agreed to participate in the study. This process continued until two controls were enrolled for each case from a given village. If we were unable to find controls in the same village as the case, a control was selected from another nearby Lahu village. All steps of the recruitment process were performed voluntarily and privately using a confidential and anonymous system. The participants were informed at the beginning of data collection that they were able to withdraw from the study at any time. Data were collected between June 2023 and March 2024.

### Research instruments

A validated questionnaire was used as the primary research instrument and was developed from a literature review [1,8,11,25] and discussion with experts and local people. Two nurses who were working in the MA cessation clinic in the regional hospital located in Chiang Rai Province and four local people (two Lahu village headmen and two Lahu village health volunteers) discussed the situations and factors related to MA use among the Lahu youths. The discussion with two nurses started with a general question about the clinic and then focused on the specific questions about the Lahu youths who attended the clinics, particularly the risk behaviors leading to MA use. Discussions with local people, both the village headman and the village’s health volunteer, were conducted in two villages. The main discussion focused on the MA use among young people and the factors influencing MA use. All the preliminary information obtained from these two forums was included for questionnaire development.

Finally, the questionnaire consisted of three parts. In part one, 13 questions were used to collect demographic information, including social media experience. For example, the questions in this section included a question asking participants about gender with three options for their response—male, female, or other—and a question asking about social media use with six options: Facebook, Line, Twitter (X), Instagram, or other. Participants checked the appropriate box for any social media platform that they used and left blank those they did not use. Regarding the income, a 15,000-baht monthly salary was used as the cutoff according to the average recent graduate (typically aged 22–24 years) in Thailand. In part two, ten questions were used to collect information on substance use behaviors, including questions such as “Do you smoke?”, with three response options: “yes”, “ever”, and “no”. When the participants responded “yes”, they were asked about their frequency of use and the availability of cigarettes. Part three was divided into two subsections: ten questions were used to detect knowledge of MA prevention and control, and another ten questions were used to detect attitudes toward MA prevention and control. In the knowledge section, each question had three options: “correct”, “incorrect”, and “unknown”. For positive questions, anyone who responded “correct” was given a score of one, and those who responded “incorrect” or “unknown” were given a score of 0. On the other hand, for the negative question, those who responded “incorrect” were given a value of “1”, whereas those who responded “correct” and “unknown” were given a value of “0”. There were five positive and five negative questions in the knowledge section. At the end of the questionnaire, those scoring ≤ 4 were defined as having poor knowledge, those scoring 5–7 points were defined as having moderate knowledge, and those scoring 8 and above were defined as having high knowledge [29,30]. With respect to attitudes, there were ten questions with three options for each question: “agree”, “neutral”, and “disagree”. There were five positive questions and five negative questions. Those who answered “agree,” “neutral,” and “disagree” with a positive question were given 2, 1, and 0, respectively. For the negative questions, those who answered “agree,” “neutral,” or “disagree” with a positive question were given 0, 1, or 2, respectively. The total score was 20. At the end of the questionnaire, those who had scores between 0 and 6 were considered to have a negative attitude toward MA use, those with scores between 7 and 13 were considered to have a neutral attitude, and those with a score of 14 or greater were considered to have a positive attitude [29–31]. In the last part, 24 questions were used to collect information about the subjects’ social environment related to MA use among the Lahu youth, such as having had peers who used MA and having had a family member who used MA (Supplement File 1).

The questionnaire was validated by the item-objective congruent (IOC) method using three external experts’ opinions on each question: one professional psychologist and one nurse who were working at the MA cessation clinic and one healthcare provider working in a health-promoting hospital. Each expert provided a score for each item with three choices: “-1” for questions not relevant to the study, “0” for questions relevant to the study but requiring improvement, and “+1” for questions completely relevant to the study. Once the scores from three experts on each item were obtained, the average score was calculated and interpreted. If the average score was less than 0.5, the question was deleted from the questionnaire. If the average score was between 0.5 and 0.7, the question was revised as suggested by the experts before they were considered further. Those questions that scored over 0.7 could be further revised or left unaltered in the final form. Afterward, the questionnaire was tested in a pilot study in Mae Chan District, Chiang Rai Province, Thailand, on 20 individuals whose characteristics were similar to those of the study population. During the pilot, seven (11) questions/points were found that required improvement before use, such as a question asking about the education of the participants, which was confusing because some people did not complete three years of secondary school and instead dropped out of school after one or one and a half years of secondary school. In this case, the question was modified to as about the participants’ completed level of their education with six (6) choices: no education, primary school, secondary school, high school, vocational school, and a bachelor's degree or above. In addition, the reliability of the ten (10) knowledge questions and the ten (10) attitude questions was evaluated using a Cronbach’s alpha of 0.78 and 0.83, respectively. Bioline methamphetamine strips were used to detect MAs in urine; these strips have a sensitivity of 98.0% and a specificity of 99.0% (Pacific Biotech Co., Ltd., Bangkok, Thailand) [32].

### Statistical analysis

The data were analyzed using SPSS (Version 24; Chicago, IL). Missing data were checked and managed properly before further analysis. Missing data were excluded from all subsequent analyses and reporting in this study. Descriptive statistics were used to present the general characteristics of the participants. Categorical data are presented as percentages, whereas continuous data are presented as the means and standard deviations. Chi-square tests and multiple logistic regression were used to detect factors associated with MA use at a significance level of α = 0.05. Collinearity was tested to determine high correlations among the variables before they were entered into the logistic regression. In this step, cannabis accessibility and opium accessibility were found to have a correlation of r = 0.87; thus, we maintained only opium accessibility in the final prediction model. The variance inflation factor (VIF) and tolerance were also tested with all the variables included in the model. No variables with a VIF > 5 and a tolerance < 0.2 were detected. Univariable logistic regression was used to explore the associations between independent variables and MA use at a p-value = 0.05. Stepwise multiple logistic regression with a probability of 0.2 was used to maintain or exclude variables from the model before fitting the final model. At the end of the analysis, age in continuous form was controlled for in the model because it was found to be a significant confounding factor in previous studies [33]. The pseudo-R-squared values and Hosmer–Lemeshow test were used to examine the model's fit and for interpretation.

### Ethical approval and consent to participate

The proposal and relevant protocols were approved by the Chiang Rai Provincial Health Office Ethical Committee, No. CROOHP 39/2566. Before the interviews, the essential information was clearly communicated in Thai and local languages to the participants. Informed consent was obtained voluntarily. The interviews were conducted in private and confidential rooms. For Lahu youth aged less than 18 years, parents were asked to provide permission for their personal information by signing an informed consent form voluntarily. The study procedures were performed in accordance with the relevant guidelines and regulations and within the Declaration of Helsinki of 1975, as revised in 2000 (5).

## Results

Four hundred and eight participants were recruited and analyzed in the study: 79.4% were male, 77.9% held a Thai national identification card, 24.0% graduated from primary school, and 34.8% had greater than a high school education. More than half (59.1%) had one partner, and 75.9% were unmarried. The parents of the cases lived together 18% less often than those of the controls did (p-value <0.001). Half of them (50.5%) had moderate knowledge of MA prevention and control, and 63.2% had moderate attitudes toward MA prevention and control (Table 1).

**Table 1:** Comparisons of general characteristics between cases and controls.

General characteristics	Total		Cases		Controls		χ^2^	p-value
n	%	n	%	n	%		
**Total**	408	100.0	136	33.3	272	66.7	N/A	N/A
**Age** (years)								
15-19	243	59.6	76	55.9	167	61.4	1.14	0.285
20-24	165	40.4	60	44.1	105	38.6		
*Mean = 19.11, SD = 2.84*								
**Gender**								
Male	324	79.4	108	79.4	216	79.4	0.00	1.000
Female	84	20.6	28	20.6	56	20.6		
**Identification card**								
Thai national card	318	77.9	100	73.5	218	80.2	2.03	0.163
Non-Thai national card/None	90	22.1	36	26.5	54	19.9		
**Education**								
Primary school	98	24.0	44	32.4	54	19.9	41.49	<0.001*
Secondary school	100	24.5	34	25.0	66	24.3		
High school/Vocational/University	142	34.8	21	15.4	121	44.5		
No schooling	68	16.7	37	27.2	31	11.4		
**Occupation**								
Private or government employee/Trader	40	9.8	7	5.1	33	12.1	45.70	<0.001*
Agriculturist	47	11.5	23	16.9	24	8.8		
Daily wage	117	28.7	47	34.6	70	25.7		
Student	123	30.1	17	12.5	106	39.0		
Unemployed	81	19.9	42	30.9	39	14.3		
**Household income** (baht/month) (n = 335)								
< 15000	293	87.5	105	95.5	188	83.6	9.54	0.002*
≥ 15001	42	12.5	5	4.5	37	16.4		
**Number of partners** (persons)								
One	241	59.1	81	59.6	160	58.8	5.34	0.072
≥ 2	53	13.0	24	17.6	29	10.7		
No	114	27.9	31	22.8	83	30.5		
**Marital status**								
Single	310	76.0	103	75.7	207	76.1	**0.20**	0.922
Married	88	21.6	29	21.3	59	21.7		
Separated/Divorced/Widowed	10	2.5	4	2.9	6	2.2		
**Parents’ living status**								
Living together	292	71.6	81	59.6	211	77.6	14.57	0.001*
Separated	53	13.0	26	19.1	27	9.9		
Divorced/either one or both died	63	15.4	29	21.3	34	12.5		
**Knowledge toward MA prevention and control**								
High	123	30.1	48	35.3	75	27.6	21.23	<0.001*
Moderate	206	50.5	79	58.1	127	46.7		
Low	79	19.4	9	6.6	70	25.7		
**Attitude toward MA prevention and control**								
Good	103	25.2	17	12.5	86	31.6	20.01	<0.001*
Moderate	258	63.2	96	70.6	162	59.6		
Poor	47	11.5	23	16.9	24	8.8		

*Significant at a p-value≤ of 0.05

Several characteristics were significantly different between the case and control groups: education, occupation, household income, parental living status, knowledge of MA prevention and control, and attitudes toward MA prevention and control (Table 1).

With respect to substance use behaviors, as shown in Table 2, 55.1% had consumed alcohol, and 43.9% had smoked. The majority (90.9%) had low stress, and friends (59.6%) were the people whom they first consulted with while in need, followed by family members and partners. Several behaviors differed between those who used MA and those who did not. Compared with the controls, those with MA use were more likely to smoke cigarettes and drink alcohol and more frequently consumed whisky, beer, cannabis, opium, and heroin (Table 2).

**Table 2:** Comparisons of risk behaviors for MA use between the case and control groups.

Substances use behaviors	Total		Cases		Controls		χ^2^	p-value
	n	%	n	%	n	%		
**Cigarette smoking**								
Ever	179	43.9	116	85.3	59	21.7	149.73	<0.001*
No	229	56.1	20	14.7	213	78.3		
**Current smoker and amount per day** (n = 116)								
Current smoker	116	100.0	92	79.3	24	20.7		
≤ *10 Cigarettes/day*	60	51.7	45	48.9	15	62.5	1.41	0.236
≥ *11 Cigarettes/day*	56	48.3	47	51.1	9	37.5		
**Alcohol use**								
Ever	225	55.1	117	86.0	108	39.7	78.65	<0.001*
No	183	44.9	19	14.0	164	60.3		
**Frequency of whisky consumption among current users** (n = 130)								
Current drinking	130	100.0	82	63.1	48	36.9		
*Once/month*	47	36.2	17	20.7	30	62.5	23.15	<0.001*
*2-4 times/month*	60	46.2	48	58.5	12	23.5		
*2-3 times/week*	23	17.7	17	20.7	6	12.5		
**Frequency of beer consumption among current users** (n = 134)								
Current drinking	134	100.0	79	59.0	55	41.0		
*Once/month*	35	26.1	14	17.7	21	38.2	9.28	0.010*
*2-4 times/month*	79	59.0	51	64.6	28	50.9		
*2-3 times/week*	20	14.9	14	17.7	6	10.9		
**Cannabis use**								
Ever	90	22.1	59	43.4	30	11.0	55.64	<0.001*
No	318	77.9	77	56.6	242	89.0		
**Frequency of cannabis use among current users of cannabis** (n = 36)								
Current use	36	100.0	21	58.3	15	41.7		
≤ *1 times/week*	23	63.9	12	57.1	11	73.3	0.99	0.319
≥ *2 times/week*	13	36.1	9	42.9	4	26.7		
**Opium use**								
Ever	45	11.0	36	26.5	9	3.3	49.56	<0.001*
No	363	89.0	100	73.5	263	96.7		
**Frequency of opium use among current users of opium** (n = 19)								
Current use	19	100.0	14	73.6	5	26.4		
*1-2 times/month*	11	57.9	7	50.0	4	80.0	3.35	0.181^a^
≥ *2 times/week*	8	52.1	7	50.0	1	20.0		
**Heroin**								
Ever	47	11.5	41	30.1	6	2.2	69.44	<0.001*
No	361	88.5	95	69.9	266	97.8		
**Frequency of heroin use among current users of heroin** (n = 23)								
Currently use	23	100.0	18	78.3	5	21.7		
≤ *1 times/week*	5	21.7	4	22.2	1	20.0	0.01	1.000^a^
≥ *2 times/week*	18	78.3	14	77.8	4	80.0		
**Stress** (ST-5)								
Moderate to very high	37	9.1	10	7.4	27	9.9	0.72	0.393
No	371	90.9	126	92.6	245	90.1		
**Social support during a personal challenge**								
Friends								
*Yes*	243	59.6	79	58.1	164	60.3	0.18	0.669
*No*	165	40.4	57	41.9	108	39.7		
Partners								
*Yes*	211	51.7	76	55.9	135	49.6	1.42	0.234
*No*	197	48.3	60	44.1	137	50.4		
Family member								
*Yes*	219	53.7	49	36.0	170	62.5	25.55	<0.001*
*No*	189	46.3	87	64.0	102	37.5		
**Social media use**								
Facebook								
*Yes*	319	78.2	106	77.9	213	78.3	0.01	0.932
*No*	89	21.8	30	22.1	59	21.7		
Line								
*Yes*	285	69.9	97	71.3	188	69.1	0.21	0.647
*No*	123	30.1	39	28.7	84	30.9		
Twitter (X)								
*Yes*	55	13.5	4	2.9	51	18.9	19.43	<0.001*
*No*	353	86.5	132	97.1	221	81.2		
Instagram								
*Yes*	57	14.0	7	5.1	50	18.4	13.22	<0.001*
*No*	351	86.0	129	94.9	222	81.6		

* Significant at p-value≤ 0.05; ^*a*^ *= Fisher’s exact test*

Facebook (78.2%) and Line (69.9%) were the most commonly used social media platforms, but use levels were not significantly different between cases and controls. Sharing personal challenges with family members differed significantly between groups, with only 36.0% of the cases doing so compared to 62.5% of the controls (p-value<0.001)—cases were much more isolated from family with respect to communication. While approximately 1 in 5 controls used Twitter (X) and Instagram, fewer cases used both (2.9% and 5.1%), both with p-values <0.001 (Table 2).

In Table 3, half of the participants (52.9%) reported having friends used alcohol, and 49.0% reported having friends smoked. A total of 62.7% reported having a family member who used alcohol, and 59.3% had a family member who smoked. Moreover, the number participants reporting peer pressure from friends to try MA (27.2%) was greater than the number reporting pressure from family members (3.7%). Most of the participants reported that they did not find it difficult to access cigarettes (98.8%) or alcoholic beverages (99.8%).

**Table 3:** Comparison of the impact of proximity and accessibility to substances between the case and control groups.

Substance use	Total		Cases		Controls		χ^2^	p-value
n	%	n			%	n	%
**Having a friend who smoked**								
Yes	200	49.0	101	74.3	99	36.4	52.03	<0.001*
No/Unsure	208	51.0	35	25.7	173	63.6		
**Having a friend who drank alcohol**								
Yes	216	52.9	97	71.3	119	43.8	27.67	<0.001*
No/Unsure	192	47.1	39	28.7	153	56.2		
**Having a friend who used cannabis, opium, or heroin**								
Yes	131	32.1	75	55.1	56	20.6	49.68	<0.001*
No/Unsure	277	67.9	61	44.9	216	79.4		
**Having a friend who used MA**								
Yes	107	26.2	78	57.4	29	10.7	102.16	<0.001*
No/Unsure	301	73.8	58	42.6	243	89.3		
**Having been pressured by a friend to use MA**								
Yes	111	27.2	85	62.5	26	9.6	128.31	<0.001*
No	297	72.8	51	37.5	246	90.4		
**Having a family member who smoked**								
Yes	242	59.3	101	74.3	141	51.8	18.90	<0.001*
No	166	40.7	35	25.7	131	48.2		
**Having a family member who used alcohol**								
Yes	256	62.7	107	78.7	149	54.8	22.15	<0.001*
No	152	37.3	29	21.3	123	45.2		
**Having a family member who used cannabis, opium, or heroin**								
Yes	44	10.8	21	15.4	23	8.5	4.60	0.041*
No	364	89.2	115	84.6	249	91.5		
**Having a family member who used MA**								
Yes	37	9.1	20	14.7	17	6.2	7.86	0.005*
No	371	90.9	116	85.3	255	93.8		
**Having been pressured by a family member to use MA**								
Yes	15	3.7	10	7.4	5	1.8	7.79	0.005*
No	393	69.3	126	92.6	267	98.2		
**Cigarette accessibility**								
Easy-to-moderate	403	98.8	135	99.3	268	98.5	0.41	0.669^a^
Difficulty	5	1.2	1	0.7	4	1.5		
**Alcohol accessibility**								
Easy-to-moderate	407	99.8	136	100.0	271	99.6	0.50	1.000^a^
Difficulty	1	0.2	0	0.00	1	0.4		
**Cannabis accessibility**								
Easy-to-moderate	111	27.2	59	43.4	52	19.1	26.96	<0.001*
Difficulty	297	72.8	77	56.6	220	80.9		
**Opium accessibility**								
Easy-to-moderate	52	12.7	33	24.3	19	7.0	24.34	<0.001*
Difficulty	356	87.3	103	75.7	253	93.0		
**Heroin accessibility**								
Easy-to-moderate	71	17.4	37	27.2	34	12.5	13.64	<0.001*
Difficulty	337	82.6	99	72.8	238	87.5		
**MA accessibility**								
Easy	256	62.7	106	77.9	150	55.1	20.15	<0.001*
Difficulty	152	37.3	30	22.1	122	44.9		

* Significant at a p-value≤ of 0.05

Many experiences were significantly different between those with MA use and those without MA use, with a greater proportion of the following among cases: having a friend who smoked; having a friend who drank alcohol; having a friend who used cannabis, opium, or heroin; having a friend who used MA; having been pressured by a friend to use MA; having a family member who smoked; having a family member who drank alcohol; having a family who used cannabis, opium, or heroin; having a family who used MA; having been pressured by a family member to use MA; and having access to MA. Compared with the controls, the cases had much greater access to cannabis, opium, and heroin (all p-values <0.001).

The study included several independent variables or predictors, including age, sex, marital status, education, occupation, family income, knowledge of MA prevention and control, and attitudes toward MA prevention and control. The dependent variable or outcome was MA use. Age was treated as a confounder in the model.

Several variables were found to be associated with MA use among Lahu youths in the univariate logistic regression model: education; occupation; household income; knowledge of MA prevention and control; attitudes toward MA prevention and control; smoking status; alcohol consumption; cannabis use; opium use; heroin use; parental living status; having friends who smoke cigarettes; having friends who use cannabis, opium, or heroin; having friends who use MA; having been pressured by friends to use MA; having family members who smoke cigarettes; having family members who drink alcohol; having family members who use cannabis, opium, or heroin; having family members who use MA; having been pressured by family members to use MA; cannabis accessibility; opium accessibility; heroin accessibility; and MA accessibility (Table 4).

**Table 4:** Factors associated with MA use among Lahu youth by univariate analysis.

Factors	Cases	Controls	OR	95% CI	p-value
**Age** (years)					
15-19	76 (55.9)	167 (61.4)	1.00		
20-24	60 (44.1)	105 (38.6)	1.26	0.82-1.91	0.285
**Gender**					
Male	108 (79.4)	216 (79.4)	1.00		
Female	28 (20.6)	56 (20.6)	1.00	0.60-1.66	1.000
**Identification card**					
Thai national card	100 (73.5)	218 (80.1)	1.00		
Non-Thai national card/None	36 (26.5)	54 (19.9)	1.45	0.87-2.36	0.130
**Number of partners** (person)					
One	81 (59.6)	160 (58.8)	1.36	0.83-2.22	0.225
≥ 2	24 (17.6)	29 (10.7)	2.22	1.12-4.38	0.022*
No	31 (22.8)	83 (30.5)	1.00		
**Married status**					
Single	103 (75.7)	207 (76.1)	1.00		
Married	29 (21.3)	59 (21.7)	0.97	0.58-1.63	0.962
Separated/Divorce/Widowed	4 (2.9)	6 (2.2)	1.34	0.37-4.85	0.656
**Parental living status**					
Living together	81 (59.6)	211 (77.6)	1.00		
Separated	26 (19.1)	27 (9.9)	2.51	1.38-4.55	0.003*
Divorced/either one or both died	29 (21.3)	34 (12.5)	2.22	1.27-3.88	0.005*
**Education**					
High School/Vocational/University	21 (15.4)	121 (44.5)	1.00		
Secondary school	34 (25.0)	66 (24.3)	2.97	1.60-5.52	0.001*
Primary school	44 (32.4)	54 (19.9)	4.70	2.55-8.65	<0.001*
No schooling	37 (27.2)	31 (11.4)	6.88	3.54-13.37	<0.001*
**Occupation**					
Private/Governance employee/Trader	7 (5.1)	33 (12.1)	1.32	0.51-3.47	0.569
Agriculturist	23 (16.9)	24 (8.8)	5.98	2.77-12.88	<0.001*
Daily wage worker	47 (34.6)	70 (25.7)	4.19	2.27-7.87	<0.001*
Unemployed	42 (30.9)	39 (14.3)	6.72	3.43-13.16	<0.001*
Student	17 (12.5)	106 (39.0)	1.00		
**Knowledge of MA prevention and control**					
High	48 (35.3)	75 (27.6)	4.98	2.27-10.89	<0.001*
Moderate	79 (58.1)	127 (46.7)	4.84	2.28-10.22	<0.001*
Low	9 (6.6)	70 (25.7)	1.00		
**Attitude toward MA prevention and control**					
Good	17 (12.5)	86 (31.6)	1.00		
Moderate	96 (70.6)	162 (59.6)	3.00	1.68-5.34	<0.001*
Poor	23 (16.9)	24 (8.8)	4.85	2.23-10.50	<0.001*
**Stress status**					
Moderate to very high	10 (7.4)	27 (9.9)	1.00		
No	126 (92.6)	245 (90.1)	1.39	0.65-2.96	0.395
**Social support during a personal challenge**					
Friends					
*Yes*	79 (58.1)	164 (60.3)	1.00		
*No*	57 (41.9)	108 (39.7)	1.10	0.72-1.67	0.669
Partners					
*Yes*	76 (55.9)	135 (49.6)	1.00		
*No*	60 (44.1)	137 (50.4)	0.78	0.51-1.18	0.234
Family member					
*Yes*	49 (36.0)	170 (62.5)	1.00		
*No*	87 (64.0)	102 (37.5)	3.76	2.30-6.21	<0.001*
**Social media use**					
Facebook					
*Yes*	106 (77.9)	213 (78.3)	1.00		
*No*	30 (22.1)	59 (21.7)	1.02	0.62-1.68	0.932
Line					
*Yes*	97 (71.3)	188 (69.1)	1.00		
*No*	39 (28.7)	84 (30.9)	0.90	0.57-1.14	0.647
Twitter					
*Yes*	4 (2.9)	51 (18.8)	1.00		
*No*	132 (97.1)	221 (81.2)	7.62	2.69-21.55	<0.001*
Instagram					
*Yes*	7 (5.1)	50 (18.4)	1.00		
*No*	129 (94.9)	222 (81.6)	4.15	1.83-9.43	<0.001*
**Cigarette smoking**					
Yes/Ever	120 (88.2)	59 (21.7)	27.10	14.91-49.14	<0.001*
No	16 (11.8)	213 (78.3)	1.00		
**Alcohol consumption**					
Yes/Ever	117 (86.0)	108 (39.7)	9.35	5.43-16.08	<0.001*
No	19 (14.0)	164 (60.3)	1.00		
**Cannabis use**					
Yes/Ever	60 (44.1)	30 (89.0)	6.37	3.83-10.58	<0.001*
No	76 (55.9)	242 (11.0)	1.00		
**Opium use**					
Yes/Ever	36 (80.0)	9 (20.0)	10.52	4.89-22.62	<0.001*
No	100 (27.5)	263 (72.5)	1.00		
**Heroin use**					
Yes/Ever	41 (30.1)	6 (2.2)	19.13	7.87-46.50	<0.001*
No	95 (69.9)	266 (97.8)	1.00		
**Having friend who smokes cigarette**					
Yes	101 (74.3)	99 (36.4)	5.04	3.19-7.86	<0.001*
No/Unsure	35 (25.7)	173 (63.6)	1.00		
**Having friend who drinks alcohol**					
Yes	97 (71.3)	119 (43.8)	3.20	2.05-4.97	<0.001*
No/Unsure	39 (28.7)	153 (56.2)	1.00		
**Having friend who used cannabis, opium, or heroin**					
Yes	75 (55.1)	56 (20.6)	4.74	3.03-7.42	<0.001*
No/Unsure	61 (44.9)	216 (79.4)	1.00		
**Having a friend who used MA**					
Yes	78 (57.4)	29 (10.7)	11.26	6.74-18.83	<0.001*
No	58 (42.6)	243 (89.3)	1.00		
**Having been pressured by a friend to use MA**					
Yes	85 (62.5)	26 (9.6)	15.77	9.25-26.86	<0.001*
No	51 (37.5)	246 (90.4)	1.00		
**Having a family member who smokes cigarette**					
Yes	101 (74.3)	141 (51.8)	2.68	1.70-4.21	<0.001*
No	35 (25.7)	131 (48.2)	1.00		
**Having a family member who drinks alcohol**					
Yes	107 (78.7)	149 (54.8)	3.05	1.89-4.89	<0.001*
No	29 (21.3)	123 (45.2)	1.00		
**Having a family member who uses cannabis, opium, or heroin**					
Yes	21 (15.4)	23 (8.5)	1.98	1.05-3.72	0.034*
No	115 (84.6)	249 (91.5)	1.00		
**Having a family member who uses MA**					
Yes	20 (14.7)	17 (6.2)	2.59	1.30-5.11	0.006*
No	116 (85.3)	255 (93.8)	1.00		
**Having been pressured by a family member to use MA**					
Yes	10 (7.4)	5 (1.8)	4.24	1.41-12.65	0.010*
No	126 (92.6)	267 (98.2)	1.00		
**Cigarette accessibility**					
Easy-to-moderate	135 (99.3)	268 (98.5)	2.02	0.22-18.20	0.533
Difficulty	1 (0.7)	4 (1.5)	1.00		
**Alcohol accessibility**					
Easy-to-moderate	136 (100.0)	271 (99.6)	N/A	N/A	N/A
Difficulty	0 (0.00)	1 (0.4)	1.00		
**Cannabis accessibility**					
Easy-to-moderate	59 (43.4)	52 (19.1)	3.24	2.06-5.11	<0.001*
Difficulty	77 (56.6)	220 (80.9)	1.00		
**Opium accessibility**					
Easy-to-moderate	33 (24.3)	19 (7.0)	4.27	2.32-7.85	<0.001*
Difficulty	103 (75.7)	253 (93.0)	1.00		
**Heroin accessibility**					
Easy-to-moderate	37 (27.2)	34 (12.5)	2.62	1.55-4.41	<0.001*
Difficulty	99 (72.8)	238 (87.5)	1.00		
**MA accessibility**					
Easy	106 (77.9)	150 (55.1)	2.87	1.79-46.00	<0.001*
Difficulty	30 (22.1)	122 (44.9)	1.00		

* Significant at a p-value≤ of 0.05

In the univariate analysis (Table 4) it is notable that the influence of friends exceeded that of the influence of family in the odds ratios in each category regarding Smoking, Alcohol use, Cannabis use, MA use, and pressure to use MA – in some cases, the Friends ORs were more than two- to three-fold higher than Family ORs. This has implications for prevention and intervention concerning social networks.”

After controlling for age in the stepwise multivariate logistic regression, ten (10) variables remained in the final prediction model (Table 5). Among these variables, seven (7) variables were found to be associated with MA use among Lahu youths. Those females were 5.29-fold (95% CI = 2.02–13.89) more likely to use MA than males. Those with easy-to-access MA had a 2.28-fold (95% CI = 1.14–4.54) higher likelihood of MA use than those who had difficulty accessing. Those who had no schooling had a 4.29-fold (95% CI = 1.17–15.73) higher likelihood of MA use than those who had vocational and university education. Those who were unemployed had a 3.92-fold (95% CI = 1.14–13.37) higher likelihood of MA than those who were students. Those who smoked or had ever smoked had a 29.08-fold (95% CI = 11.57–73.12) greater likelihood of MA use than those who never smoked. Those who drank alcohol had a 2.53-fold (95% CI = 1.05–6.11) higher likelihood of MA use than those who had never drunk. Those who had high knowledge scores of MA prevention and control would be more likely to use MA than those who had lower scores (AOR = 1.29; 95% CI = 1.11–1.50).

**Table 5:** Factors associated with MA use among Lahu youth by multivariate logistic regression.

Factors	AOR	95% CI	p-value
**Sex**			
Male	1.00		
Female	5.27	1.98-13.82	<0.001*
**MA accessibility**			
Easy	2.35	1.18-4.65	0.015*
Difficulty	1.00		
**Married status**			
Single	1.00		
Married	0.76	0.31-1.84	0.543
Separated/Divorce/Widowed	0.48	0.75-3.02	0.431
**Parental living status**			
Living together	1.00		
Separated	1.73	0.70-4.31	0.236
Divorced/either one or both died	1.70	0.70-4.10	0.239
**Education**			
High School/Vocational/University	1.00		
Secondary school	0.86	0.29-2.57	0.783
Primary school	1.46	0.42-5.02	0.552
No schooling	4.07	1.12-14.75	0.033*
**Occupation**			
Private/Governance employee/Trader	1.53	0.34-6.87	0.578
Agriculturist	2.45	0.52-11.49	0.256
Daily wage worker	2.85	0.79-10.24	0.108
Unemployed	3.75	1.10-12.76	0.034*
Student	1.00		
**Knowledge of MA prevention and control (Score)**	1.30	1.12-1.51	<0.001*
**Cigarette smoking**			
Yes/Ever	27.08	11.07-66.22	<0.001*
No	1.00		
**Alcohol consumption**			
Yes/Ever	2.68	1.12-6.38	0.026*
No	1.00		
**Sex**			
Male	1.00		
Female	5.29	2.02-13.89	0.001*
**Cigarette accessibility**			
Easy-to-moderate	7.65	0.25-229.55	0.241
Difficulty	1.00		
**MA accessibility**			
Easy	2.28	1.14-4.54	0.019*
Difficulty	1.00		
**Married status**			
Single	1.00		
Married	0.80	0.33-1.97	0.640
Separated/Divorce/Widowed	0.48	0.07-3.07	0.433
**Parental living status**			
Living together	1.00		
Separated	1.71	0.68-4.27	0.246
Divorced/either one or both died	1.85	0.75-4.54	0.176
**Education**			
High School/Vocational/University	1.00		
Secondary school	0.84	0.28-2.53	0.762
Primary school	1.46	0.42-5.06	0.543
No schooling	4.29	1.17-15.73	0.028*
**Occupation**			
Private/Governance employee/Trader	1.56	0.34-7.17	0.567
Agriculturist	2.24	0.47-10.69	0.309
Daily wage worker	2.81	0.78-10.15	0.114
Unemployed	3.92	1.14-13.37	0.029*
Student	1.00		
**Knowledge of MA prevention and control (Score)**	1.29	1.11-1.50	0.001*
**Cigarette smoking**			
Yes/Ever	29.08	11.57-73.12	<0.001*
No	1.00		
**Alcohol consumption**			
Yes/Ever	2.53	1.05-6.11	0.038*
No	1.00		

* p-value≤ = 0.05; age was controlled in continuous data form in the analysis

## Discussion

Multiple factors contribute to the risk of MA use among Lahu youths, including sex, MA accessibility, low education, unemployed status, knowledge of MA prevention and control, smoking, and drinking alcohol.

Lahu females had a significantly greater likelihood of MA use than males. A study reported that females had a greater number of MA use compared to males [34]. Females were reported to have a greater prevalence of MA use in their lifetime than males in Thailand, even though males had earlier use of MA than females [35]. A study conducted in the United States [36] reported that females had a higher prevalence and frequency of use than males. Weight reduction was the main reason to use MA among females [37]. However, among the Lahu youths, particularly females, the main reason to use MA could be to increase their farming products which is as after using MA, people could spend more time working their farm [11,38].

Lahu youths with low education levels had a significantly greater likelihood of MA use than those with high education levels did. Several studies [12,39] have reported that young people with low education levels have a greater likelihood of engaging in MA use than did those with high education levels do. Another study reported that young people who did not attend school spent time with their friends and eventually used MA [10]. A study conducted in the United States reported that there was a strong association between MA use and low socioeconomic status [40]. A study conducted among the Akha and Lahu people in northern Thailand revealed that those who failed their studies and dropped out of school had a greater likelihood of initiating MA than did those who attended school did [11,25]. Among Lahu youths, those who have a low level of education and who drop out of school have a greater likelihood of using MA because their health literacy is low and because they are more easily pressured by their peers while living together outside the school. Most Lahu villages are located in border and remote areas where access to MA in their daily life is necessary for engaging in MA use.

In our study, it was found that the unemployed status played a role in influencing MA use among the Lahu youths. A study conducted in Iran indicated that unemployed individuals were at risk of MA use than other groups [41], and those who were unemployed individuals were more at risk of relapsing to use substances, including MA [42]. Several studies confirmed that the unemployed working status had a greater risk of MA use than the full-time working status [43,44]. In Thailand, it was found that unemployed young adults were at a higher risk of using MA than employed individuals. The Lahu youths who were not working could spend their time with their friend and be persuaded to use MA. In addition, those who were unemployed could be stressor from having no job and income, which could motivate them to use MA.

Seemingly paradoxically, those who had higher knowledge of MA prevention and control had a significantly greater likelihood of MA use than those at low levels did. A study from Bari Imam [45] reported that nearly half of the participants continued to use their current drug despite knowing the adverse effects. Another study revealed that those with MA use had high knowledge levels of the especially harmful effects of cigarettes, alcohol, and cannabis [46]. For our study, cases were recruited from a list of MA treatment clinics; it is highly possible that those cases in the study received education regarding MA use from health professionals. By contrast, the controls were selected from community members and therefore had low-level knowledge of MA prevention and control. MA users not attending such clinics may also have a much lower level of knowledge. Although a knowledge behavior gap between cases and controls was observed, this study did not focus on this point. In addition, assessing knowledge and MA use could be impacted by the temporal sequence in this study because both knowledge and MA use are assessed simultaneously.

Cigarette smoking and alcohol consumption were associated with a greater likelihood of MA use among Lahu youths than among other groups. Several studies [25,47] have reported a higher rate of MA use among hill tribe people in Thailand who smoke than among those who do not smoke. A systematic review [48] and a study conducted in South Africa [49] confirmed the increased rate of MA use among those who engaged in smoking behavior among young adults. A study conducted in Iran reported that smoking tobacco before MA use was common [2]. Alcohol consumption has been reported as a risk behavior to MA use in several studies [11,43]. A study conducted in Vietnam confirmed the strong association between alcohol use and MA use [50]. Several studies also reported that alcohol use was a common pattern that precedes methamphetamine use [2,51]. Among Lahu youths, cigarette smoking and alcohol were very common because of the accessibility in the villages, and youths may have started using these kinds of substances before starting MA. For these reasons, drug use transition and polydrug use behavior might occur among Lahu youths.

MA accessibility was associated with MA use among Lahu youths. Like other studies in Thailand, accessibility led to individuals initiating MA use [9]. Moreover, the area of shared borders has increased, and MA access near borders is more likely [11]. According to our study area, Lahu youth in remote areas share borders with significant MA smuggling areas [15] with MA available at extremely affordable prices [18], which increases access to MA among Lahu youths.

## Limitations

In accordance with the research methodology used in this case‒control study, three approaches were used to identify controls: verbal questions, a urine test, and three references from community members. Although these were the best methods for identifying the controls, they did not guarantee that all the controls had never used MA. An advantage of a case‒control study is that it can assess the causal relationship between variables using the odds ratio, an indirect method. In this study, cases were randomly selected from clinic lists, which may not be a good representation of all MA users, particularly those who did not attend a clinic and could not be selected as cases. These two groups may differ in their characteristics. In addition, the study focused on Lahu youths; therefore, it is unclear whether the findings can be applied to other tribes who are living in the same areas. One more significant point of the study is the illegal status of MA in Thailand, leading some people to not disclose their MA use. This could be one of the concerns of misclassification in the present study, particularly in the control group. Finally, although all selected cases were willing to participate in the study, three selected cases had moved to other provinces and could not be reached for further information.

## Conclusion

Several individual, interpersonal, and structural factors contribute to MA use among Lahu youths. Females, no schooling, unemployed status, high knowledge of MA, and substance use behaviors are associated with MA use among Lahu youths. Creating a national policy for promoting access to education among Lahu youth who are living in remote areas and special training programs that fully engage local people to improve their preventive behaviors in MA prevention and control should be carefully created and implemented in Lahu villages, which are located in remote and border areas. Moreover, existing laws and regulations related to reducing the availability of MA and substances in the villages could be complemented to reduce the size of the problem. For further research, a community-based quasi- or full experimental study focusing on preventing new MA use among Lahu and other hill tribe youths should be conducted.

## Supporting information

S1 Appendix Question guide. (DOCX)

S1 File STROBE. (DOC)
