# Supplementary material for: Factors influencing methamphetamine use among Lahu hill tribe youth in Chiang Rai, Thailand: A case‒control study
Source: PLoS One. 2026 Mar 12;21(3):e0344775. doi: 10.1371/journal.pone.0344775 (PMC12981475; doi:10.1371/journal.pone.0344775)
Supplement: S1 Appendix — (DOCX) [file pone.0344775.s001.docx]

**QUESTIONNAIRE**

# Researcher part

1. MA use history (Seft-assessment)


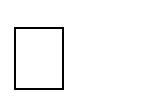


Yes


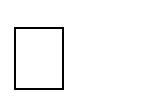


No

1. Have MA treatment experienced (Urine test result)


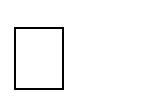


Yes


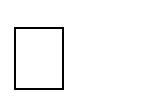


No

1.
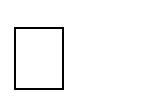

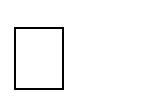

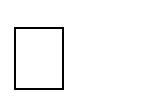
Never use of MA by Trible-check method Head villager Family Friend (Choice “C” Only asking in a questionnaire of cases)

**Part 1:** Demographic characteristic


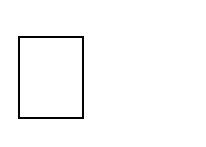
(Please check ( ) into the box)

- 1. Age years
  2.
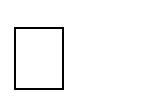
Gender Male
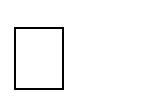
Female
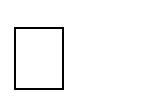
Others……………………
  3. Identification card


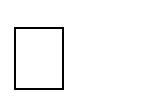
Thai national card (Light blue card)


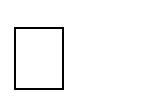

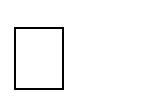
Non-Thai national card (White or Pink card) Nothing

- 1. Education level


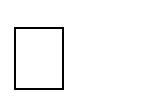

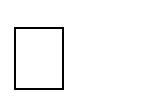

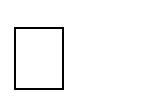

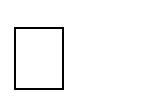
No educate Primary school Secondary school High school


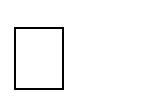

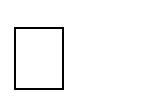
Vocational school / Technical College Bachelor's degree or above

- 1. Thai languages skill

| 1.5.1 Reading Thai | Excellent 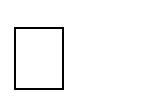 | Intermediate 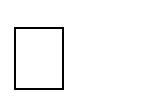 | Can’t 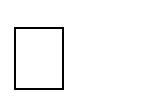 |
| --- | --- | --- | --- |
| 1.5.2 Writing Thai | Excellent 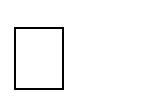 | Intermediate 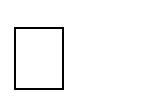 | Can’t 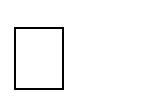 |
| 1.5.3 Speaking Thai | Excellent 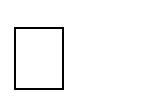 | Intermediate 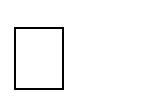 | Can’t 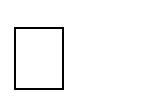 |

- 1. Occupation


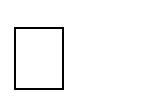

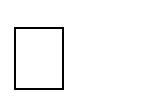
Unemployed Private employee


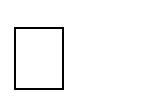

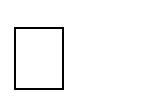
Governance employee Treader


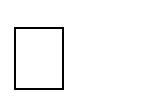

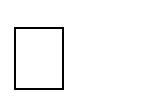

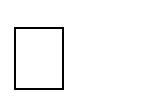
Agriculturist Daily wage worker Student

- 1. House hold income (including everyone)

Monthly income… Bath.

- 1. Partner


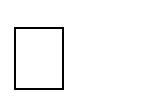

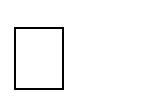
No / Never have Have / Ever had


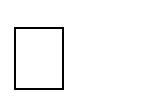

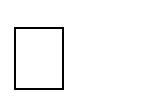
One person >2 persons

- 1. Married status


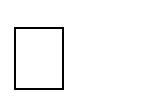

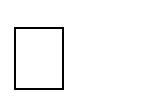

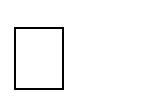

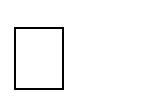
Single Married Separate Divorce / Widowed

- 1. Parental status


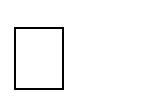

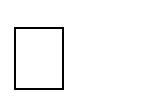

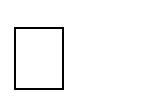

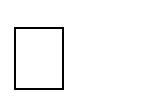

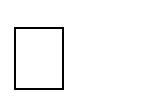

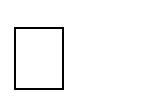
Living together Separated Divorce / Widowed Father die Mather die Both die

- 1. Stress status

| Symptoms | Never | Sometime | Often | Regular |
| --- | --- | --- | --- | --- |
| **1.** Within 2 - 4 weeks past you’re having Insomnia or  Hypersomnia |  |  |  |  |
| 2. Within 2 - 4 weeks past your loss of concentrate |  |  |  |  |
| **3.** Within 2 - 4 weeks past you’re having irritability |  |  |  |  |
| 4. Within 2 - 4 weeks past you’re feel boring |  |  |  |  |
| 5. Within 2 - 4 weeks passed you don't want to meet others |  |  |  |  |

- 1. When you face with problems who will you share with? (can answer more than one)


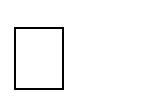

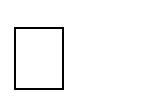

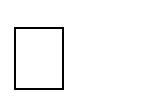

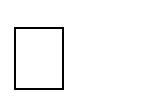

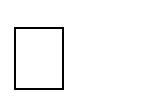
No one Friend Partner Family member Others………………

- 1. Social media use (can answer more than one)


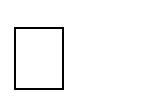

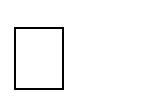

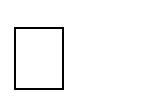

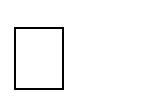

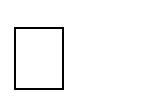

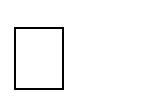
Facebook Line Twitter (X) Instagram Other……………… Never use


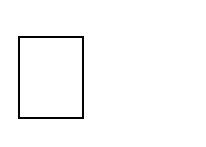
**Part 2:** Substances use behavior (Please check ( ) into the box)

- 1. Are you smoking?


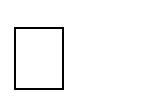


Yes


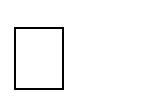


Ever


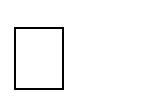


No

No. of cigarette per day


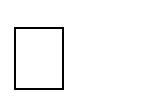
10 rolls 11 – 20 rolls


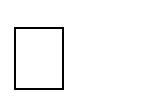


<


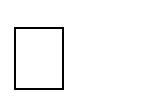
21 – 30 rolls 31 rolls


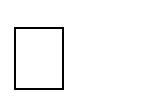


>

Do you think cigarette is easily accessible if want to?


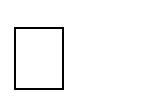

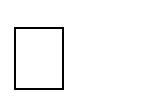
Difficulty Moderate


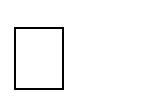


Easy

- 1. Are you drinking alcohol?


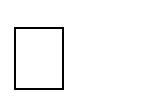


Yes


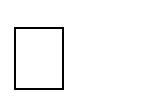


Ever


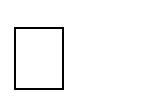


No

How often that you drinking whisky?


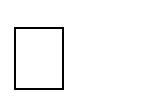
once/month

- - - 3 times/week


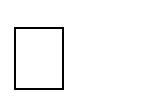


2

- 4 times/month

1. times/week


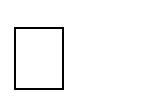


2


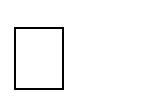


>


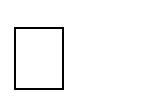

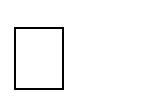
Among of whisky, you drink per times Among…………………… Flask Bottle

How often that you drinking beer?


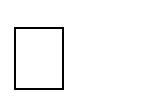
once/month

– 3 times/week


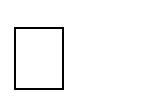


2

- 4 times/month

4 times/week


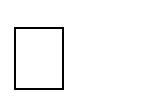


2


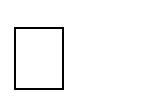


>


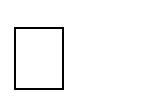

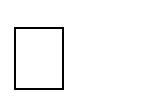
Among of whisky, you drink per times Among…………………… Can Bottle

Do you think Alcohol beverage is easily accessible if want to?


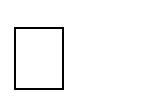

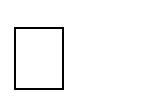
Difficulty Moderate


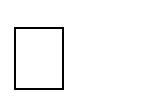


Easy

- 1. Are you use cannabis


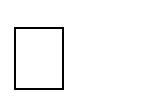


Ever


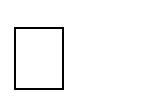


No

How often that you use cannabis?

- - - **
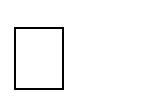
2** times/month once/week


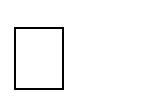


Yes


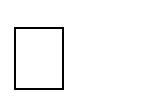


**1**

- - - 3 times/week – 6 times/week


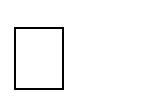


2


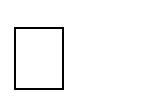


4


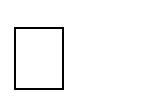
Everyday

Among of cannabis, you use per times… rolls

Do you think cannabis is easily accessible if want to?


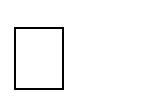

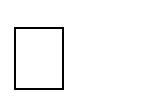
Difficulty Moderate

Easy

- 1. Are you use opium?

Ever

No

How often that you use opium?

- - - **2** times/month once/week

Yes

**1**

4

- - - 3 times/week Everyday

2

- - - 6 times/week

Among of cannabis, you use per times… rolls

Do you think opium is easily accessible if want to?

Difficulty Moderate

Easy

- 1. Are you use heroin?

Ever

No

How often that you use opium?

- - - **2** times/month once/week

Yes

**1**

4

- - - 3 times/week Everyday

2

- - - 6 times/week

Among of cannabis, you use per times… pill(s)

Do you think opium is easily accessible if want to?

Difficulty Moderate

Easy

# (2.6 Only asking in a questionnaire of cases)

- - 1. Have you ever smoked before using MA?

Yes

No

- - 1. Have you ever drinking whisky/beer before using MA?

Yes

No

- - 1. Have you ever use cannabis before using MA?

Yes

No

- - 1. Have you ever use opium before using MA?

Yes

No

- - 1. Have you ever use heroin before using MA?

Yes

No

**Part 3:** Knowledge and attitude towards MA

- 1. Do you ever curious about enjoyment form MA use?

Sometimes Never

Ever

- 1. Do you ever curious about taste form MA use?

Sometimes Never

Ever

- 1. Do you ever curious about feelings experienced MA use?

Sometimes Never

Ever

Knowledge towards MA

| Question | Correct | Incorrect | Unknow |
| --- | --- | --- | --- |
| 1. MA is illegal in Thailand |  |  |  |
| 2. MA cause the brain tissue damages, resulting in negative effects such as memory loss and  motion control, etc. |  |  |  |
| 3. People who regularly use MA must be  increased among MA gradually. |  |  |  |
| 4. Consuming MA causes tooth decay and gum  inflammation. |  |  |  |
| 5. MA and Ice (Crytal-MA in Thai word) are  similarly substance |  |  |  |
| 6. If consume MA continuously, will become addicted and causing MA Withdrawal syndrome  when lack of MA consuming |  |  |  |
| 7. Memory loss is not caused by taking MA. |  |  |  |

| Question | Correct | Incorrect | Unknow |
| --- | --- | --- | --- |
| 8. Diarrhea and joint pain found in people who  have MA Withdrawal syndrome |  |  |  |
| 9. Consuming MA increases the risk of  contracting Tuberculosis. |  |  |  |
| 10. Taking MA causes personality changes such  as aggressive and irritability |  |  |  |

Attitude towards MA

| Question | Agree | Neutral | Disagree |
| --- | --- | --- | --- |
| 1. MA have negative effects on the body more  than beneficial to the body |  |  |  |
| 2. MA can increase energy for workload |  |  |  |
| 3. MA increase the ability to working |  |  |  |
| 4. MA can reduce stress |  |  |  |
| 5. MA can release body pain |  |  |  |
| 6. MA doesn’t help you to increase speaking skill |  |  |  |
| 7. MA can stimulate to increase pleasure during  sexual intercourse |  |  |  |
| 8. MA can help to losing your weight |  |  |  |
| 9. MA can’t increase personal confidence in  socializing with others |  |  |  |
| 10. Consume MA is normally thing for people in  the village |  |  |  |

**Part 4:** Social and environmental factors

- 1. Have you ever argued with another person and been injured?

Never

Ever

- 1. Have you ever been physically harmed by another person?

Never

Ever

- 1. Have you ever been physically harmed by family member?

Never

Ever

- 1. Have you ever been bullied about your appearance in the past 12 months?

Never

Ever

- 1. Have you ever been bullied about your economic status in the past 12 months?

Never

Ever

- 1. Have you ever been bullied about your Lahu cultural in the past 12 months?

Never

Ever

- 1. Have you ever been bullied about your Thai communicate skill in the past 12 months?

Never

Ever

- 1. Do you have close friend who smoking?

Unsure

Yes

No

- 1. Do you have close friend who drinking alcohol beverage?

Unsure

Yes

No

- 1. Do you have close friend who use cannabis?

Unsure

Yes

No

- 1. Do you have close friend who use opium?

Unsure

Yes

No

- 1. Do you have close friend who use heroin?

Unsure

Yes

No

- 1. Do you have close friend who use MA?

Unsure

Yes

No

- 1. Have your friends ever invited you to use MA?

Unsure

Yes

No

- 1. Do you have family member who smoking?

Yes

No

- 1. Do you have family member who drinking alcohol beverage?

Yes

No

- 1. Do you have family member who use cannabis?

Yes

No

- 1. Do you have family member who use opium?

Yes

No

- 1. Do you have family member who use heroin?

Yes

No

- 1. Do you have family member who use MA?

Yes

No

- 1. Have your family ever invited you to use MA?

Yes

No

- 1. If a family member knows you use MA, will they be able to accept that? Acceptable Unacceptable Unsure
  2. In your opinion and experience, how accessibility of MA to seek if you want to? Difficulty

Easy

- 1. In your opinion and experience, how accessibility of MA to seek if you want to? Difficulty

Easy

- 1. How about MA use preventive activity in your village, do you recognize that?

Permanent Temporary

Have

Not have No idea
