# Supplementary material for: Factors influencing methamphetamine use among Lahu hill tribe youth in Chiang Rai, Thailand: A case‒control study
Source: PLoS One. 2026 Mar 12;21(3):e0344775. doi: 10.1371/journal.pone.0344775 (PMC12981475; doi:10.1371/journal.pone.0344775)
Supplement: Attachment — Submitted filename: ReviewReport_2025_PONE-D-25-27632.pdf [file pone.0344775.s003.pdf]

Review Report: **Factors influencing methamphetamine use among Lahu hill tribe youth Chiang Rai, Thailand: A case–control study**

Dear authors,

It is remarkable that you have clearly pointed out MA as a public health issue, and the Lahu youth population is vulnerable to that. Yet, I would like to clear some concerns and provide several comments to further enhance the understandability of the manuscript.

Abstract:

The abstract is well structured. However, there are some minor concerns from my side, which I might need a little clarification or a slight modification to the written abstract. Please address them accordingly:

1. Under the methods: "… The participants were recruited from…". Could you please clarify the sampling method here?
2. The authors stated, "A validated questionnaire was used to collect data, and multiple logistic regression was used to detect association factors at $\alpha = 0.05$."
3. The use of the term "detect association factors at $\alpha = 0.05$" is understandable. However, to make it technically sound, would you consider rewording the statement to provide an idea like "…with statistical significance set at $\alpha = 0.05$…"? Such a clear expression would avoid any ambiguity.
4. When I was referring to the data collection instrument of your study, I noticed that the expected outcomes of your study are not always binary. If the outcome measures are binary, it is technically accurate to use the "multiple logistic regression"; otherwise, it is recommended to use linear or ordinal regression for the data analysis. Please recheck your data analysis plan and supplement with related information in your methods section.

Introduction:

Informative approach in introducing the scope of your research area and the aims. You have mentioned the neurobiological effects of using MA and also the societal consequences. Special emphasis on the socio-economic disparities in the context of Thailand's geographical risks is an important fact.

1. However, the lines 11 to 19 do not create a bridge between the MA use with the socio-economic disparities that you have emphasised there, including the 'education'. Could you please elaborate on the association between the MA use and the mentioned socio-economic factors in the lines? Or could you briefly mention why Lahu youth are at higher risk by referring to the socioeconomic factors and geographic isolation?

It is appreciated that you have cited sources from both the Thai-originated sources and the international sources. When considering the logical flow of your introduction, it can be guessed that you have focused first on bringing the readers' attention to the global scenario, then Thailand, then Chiang Rai in Thailand, and the Lahu youth.

2. However, it would be better if you could also add a paragraph to emphasise the gaps in the literature, which should be a critical element of an introduction to a manuscript. Because, in your introduction, it was not explicitly stated why previous research was not sufficient, or why the previous research was not available in your area of interest as a controlled case study.
3. Lines 22 to 24: You mentioned the study objective. However, there was no justification in the introduction on why the case-control method was selected. Could you briefly mention why you have selected the case-control study design?
4. It is highly recommended to clarify/state the hypotheses rather than hinting it in a descriptive way through the research aim. Please consider modifying the way you report the aims by giving more focus into your study's hypotheses.
5. It is suggested to use your statements in lines 20-22 in the discussion section rather than in the introduction section.
6. Because your overall conclusion is to emphasise the need for "effective interventions and changes in policy" the introduction must expand a bit its concluding remarks by supplementing information about the policy relevance of this study.

Materials and Methods:

The methods section is satisfactorily planned. However, the following comments/ questions will help you to increase the readability and broaden the understanding of your study for readers with less understanding of statistical methods. Please consider modifying your writing based on the following:

1. Line 27: The same comment (comment 3 of the introduction) applies here. You may address it under study design.
2. Lines 29 to 30: Please consider using full sentences. Could you rewrite for better readability?
3. The sample size was calculated appropriately. However, to ensure expanded readability, it is better if you could add notes for the mentioned variables/constants/functions/symbols in the equation. This would help readers who may not have a wider understanding of equation-related statistical symbolic expressions.
4. Line 10 onwards: To enhance the objective verification, could you mention whether there were complementary self-reports compared to the urine test results? If the results were not complementary, what was the next step?
5. The triple triple-check method is obviously a strong method to ensure the selection validity of your sample. Yet, despite the triple-check, there might still be social desirability bias among the controls as the MA use can be under-reported because of its stigma. Could you please try considering this as a fact when addressing the previous comment, too?
6. Also, the controls from the same village might share unmeasured confounders such as access to the MA. How have you addressed this as a fact?

7. If I have understood your inclusion and exclusion criteria for the 'cases' accurately, there is a hint of recall bias on self-reported MA use, as there is an absence of a biochemical verification. This point is recommended to address in the methods section or as a limitation in the discussion section.
8. Line 21: The claim "a validated questionnaire…" must be reported cautiously. The explained process does not hint at a full validation process of the questionnaire. Please consider this a critical comment.
9. Line 22: Please cite the literature that you have referred to when developing the question items.
    a. "discussion with experts and local people…" How did you use the discussion findings to develop the questionnaire? Please explain in brief.
10. It is not very clear how you have analysed the responses for the question related to the social media lists (binary vs. frequency?). Could you please explain?
11. It is better to provide more information on your scoring system when managing the data.
    a. In the 'knowledge section,' you have combined 'no' and 'not sure' into the same score (= 0).
    b. In the 'attitude section', you have scored neutral responses as 1. This might mask the true attitude of the respondent as the Likert scale is a 5-point one. Therefore, please clarify your scoring methodology and state if any sensitivity analysis was considered to ensure the rigour of the scoring methodology.
12. Line 19: "The questionnaire was determined to be valid by three external experts in the field". Could you mention under what criteria the questionnaire was considered as validated?
    a. Please briefly mention the background of the experts.
13. Did any of the pilot study results influence you to revise the questionnaire?
    a. Is there any significant methodological outcome that you witnessed through the pilot study?
    b. And for what exact purpose did you administer the pilot test?
14. Both under the 'study sample' and the 'recruitment procedure', there is some overlapping information on the triple-check method. Please consider revising.
15. There is some clarity to be made on the variable selection, as this was not highlighted/connected well throughout the "introduction" section. Couldyou please explain the rationale for including the variables if the mentioned variables were chosen a priori based on theory or empirical univariate results?
16. In the step-wise regression, did you pre-specify the key confounders in the model regardless of the significance? Please elaborate a little.
    a. Then you may explain whether you have proceeded step-wise for exploratory variables.
17. Please briefly mention whether there was any correlation between variables such as education, occupation and income (VIF/tolerance).

18. Could you tell how the continuous predictors were handled?
19. Because the interpretations of odds/ratios can be affected, please mention how the knowledge/attitude scores were treated under the variable type.
20. Were there any missing data?
    a. If 'yes', how did you handle those?

Results:

The way you decided to report the results indicates the structural planning of reporting the results.

1. However, you may still reduce the redundancies in the text by streamlining the text and the table. It is suggested to refer the readers to tables rather than repeating a lot of context through a text, which can be easily grasped through the table figures.
2. There were inconsistencies noted in how the '*p*' was reported in terms of expressing it through the words. You may reconsider whether you use the term "p value" or "α" based on the referencing and reporting guidelines given by the journal.
3. Could you please include the top univariate associations in the text with *p*-values/ ORs rather than forcing the readers to consult the tables? Univariate statistics play a crucial role in your study. Hence, much emphasis should be on this, rather than allocating more space in the text to the descriptive data explanations.

Please ignore if you have addressed this comment through the methods section under statistical analysis. If note, please address:

4. Why are the other variables, except the age, absent in the multivariate model? Did you drop those in a step-wise regression model? Please mention the *p*-value if the missing variables were statistically not significant.

Discussion:

The discussion section has many strong points. You have directly addressed the key findings with citations to build a discussion. The most significant aspect is that you discussed the Lahu-specific vulnerabilities.

A. This will be an interesting section from a global point of view if you could <u>compare Lahu findings in-depth</u> to other Thai hill tribes or <u>global indigenous youth</u>.

Engaging the counterintuitive results and transition logic on substance use would catch the attention of the readers. However, you may still add more depth to the discussion by paying attention to the following aspects/comments/suggestions:

1. You may still discuss peer/family pressure (given the aOR = 13.79) in depth. You may discuss how the extreme risk of peer persuasion suggests MA use due to its communal living or limited alternative recreations.
    a. You may refer to social learning theory or cultural connaissance theory if you need more insights 😳.
2. "...cases learned from clinics…" Did cases score higher because they overestimated their knowledge? Please discuss. Because there may be a misclassification bias or a knowledge-behaviour gap.
3. It is great that you have mentioned the remote village access to MA. But could you discuss any recommendations based on any available national action plan on MA, on any micro-level strategies that Thailand proposed?
4. There are repetitions in the highlights of smoking and heroin. Could you synthesise your repeated claims into one paragraph (you may emphasise local drug sequencing patterns)?
5. Peer influence and accessibility related facts are repetitively discussed in the discussion section. Could you consider merging the facts into one subsection with a deeper synthesis?
6. In the conclusion, please make sure to synthesise your novel contributions by mentioning how the Lahu tribe findings may differ from the other domestic or international hill tribes.
7. The policy implications that you suggest should be more specific, actionable steps rather than being vague ("special training programmes" ??? like what ?). Could you consider proposing specific models by referring to a previous successful intervention or programme models?
8. It would be great if you could acknowledge the limitations of the study design and the biases. Please acknowledge the limitations of your study and provide suggestions/directions for further research.

I hope that the comments/questions will improve the presentation of your valuable contribution and increase the capacity of dissemination of the found knowledge by you. Congratulations!
