# Supplementary material for: Factors influencing methamphetamine use among Lahu hill tribe youth in Chiang Rai, Thailand: A case‒control study
Source: PLoS One. 2026 Mar 12;21(3):e0344775. doi: 10.1371/journal.pone.0344775 (PMC12981475; doi:10.1371/journal.pone.0344775)
Supplement: Attachment — Submitted filename: MA_Reviewers commenst.pdf [file pone.0344775.s007.pdf]

Response to reviewers comments

Dear Editor and reviewers,

Thank you very much for the opportunity to revise again with valuable comments and feedback from you. We have carefully revised based on all your comments and feedback. We re-analyzed the whole data set by treating age and knowledge in continuous forms, and the final model looks better than the previous one. Please see details on our feedback below, and very hope you are happy with this version.

Thank you,

TK

Thank you for submitting your manuscript to PLOS ONE. After careful consideration, we feel that it has merit but does not fully meet PLOS ONE's publication criteria as it currently stands. Therefore, we invite you to submit a revised version of the manuscript that addresses the points raised during the review process.

The three original reviewers have assessed your revised manuscript and thank you for addressing the issues they raised during the first round of review.

However, each of the three reviewers still has some requests for further improvements to the manuscript - please see their comments below.

I also have a concern with the categorisation of continuous variables, specifically the knowledge and attitude scores, although this applies to age as well.

1. You have dichotomised or trichotomised these variables for the logistic regression analysis. However, you have not provided a reason why knowledge and attitude scores should be split into three categories, or why the cutoffs should differ across these two measures. None of the sources you cite (refs 29-31) treat continuous variables in this manner (with the exception of a single analysis in ref 29). Unless there is good reason to do so (e.g., bimodal data), continuous variables should not be changed into categorical variables. Knowledge, attitude, and age can all be entered as continuous predictor variables in logistic regression. Please could you re-run your analyses with continuous predictors where relevant/appropriate?

: Thank you very much for the comments. We have re-analyzed the whole process in a stepwise with pr.(0.2) for selection variables into the model, and treated age and knowledge in continuous forms. The final model, ten (10) variables were retained in the model for predicting, and seven 97) variables were found to be associated with MA use. We have revised all relevant information, including Table 5. Please see page 2, lines 3-13; page 22, lines 3-15, table 5, discussion and conclusion sessions.

We do hope that you are happy with this version.

Reviewers' comments:

Reviewer #1: Dear Authors,

2. Thank you for responding to the review comments and being patient enough with providing further clarifications. There is one more suggestion from my side. Could you please consider replacing the statement "Missing data were checked before further analysis" to indicate that missing data were not reported?

: Thank you for the comment. We have modified the statement to “Missing data were checked and managed properly before further analysis.” Please see page 7, lines 29-30.

Reviewer #2: PONE-D-25-27632 Resubmission Review

Thank you to the authors for the responsive revisions.

The major issues have been addressed in the revision including correction of tables column summing and introduction of a separate table for the multivariate analysis (Table 5). The issue of the wide confidence intervals for certain variables was investigated concerning possible multicollinearity (with one variable dropped) using variable correlation and VIF (variance inflation factor) investigation. Small cell size was also identified as a possible culprit.

Below are a list of minor edits from reading the revised manuscript. I do not need to see these edits. Here again page and line # is indicated as page.line#. a=>b means change a to b. [Comments in brackets simply clarify the change.]

3.- Throughout : p value or p-value
[as the editor prefers ... in the US it is mostly p-value]

: Thank you for the notice. We totally agreed with you and have changed to p-value all points used in the paper.

4.- 4.28: :: => : [delete one colon]

: Thank you, it's improved

5.- 7.19: drop => dropped

: Thank you, it's improved

6.- 7.24: were => was [singular, agrees with 'reliability' as subject]

: Thank you, it's improved

7.- 8.9: determine => examine

: Thank you, it's improved

8. - 21.9: fold(=> fold ([insert space]
: Thank you, it's improved

9.- Page 22 Table 5: [add * after Yes/Ever p-value concerning Heroin use]
0.001 => 0.001*
: Thank you, it's improved

10. - 23.16: Either smoking or heroin use, was identified
=> Both smoking and heroin use were identified

: Thank you, it's improved

11. - 23.22: particularly young adults => particularly among young adults
: Thank you, it's improved

12. - 23.26: use of cod heroin and MA => use of codrug heroin and MA
or: => use of heroin and MA [not sure what 'cod' means]
: Thank you, it's improved, it is "codrug.."

13. - 24.11: MA access => MA access near borders
: Thank you, it's improved

14. - 24.24: difficult to => unclear whether
: Thank you, it's improved

15.- 25.9: laws => existing laws
[as in complement existing laws? Or perhaps meaning strengthen existing laws?]
: Thank you, it's improved

Reviewer #3: I thank the authors for their thorough effort in responding to our feedback. It shows that you have tried to answer to the variety of suggestions and comments reviewer put forward. While you've demonstrated understanding of the methodological concerns raised, a significant disconnect exists between your explanations to reviewers and what appears in the manuscript. Readers would greatly benefit from the clarifications you provided in your responses being incorporated into the manuscript text.

16. Your responses contained valuable explanations for: sample size calculation rationale based on the Thailand narcotics control report; the logic behind different income thresholds (15,000 baht for graduates vs. $1,400 poverty line); your data completion procedures that minimized missing values; the rationale for age-only adjustment in the final model; and the categorization of age variables. These explanations must be incorporated into the manuscript as they address fundamental questions readers may have about your methodology.

: Thank you for such great comments. We have added information on the main manuscript. Please see page 4, lines 10-11/ page 7, lines 33-34/ page 8, lines 1.

17. Additionally, while you explained age adjustment to reviewers, the final model's exclusion of other significant univariate factors (education, occupation, income, family status) despite your acknowledgment of "wide CIs due to small cells" suggests model instability rather than appropriate selection. Consider reporting exact cell counts for problematic variables and discussing precision limitations explicitly or use formal model selection criteria to justify your approach.

: Thanks for the great comment. We have revised the whole process of the analysis again using the stepwise method at selection propagability pr.(02) after putting all variables in the model and treating knowledge and age at continuing forms as recommended. It was found that 10 variables were found to be associated with MA use. Among the remained variables in the stepwise mode, three variables presented in the previous version were not added as the predictors: heroin use, having been persuaded by a friend, and smoking cigarettes.

While another six variables were added into the model as the predictors for the MA use: sex, cigarette accessibility, marital status, parent living status, occupation, and alcohol assumption (see Table 5). We have seriously considered together among the authors and agreed that this new model is much better than the previous one and is be used as our final model. At the end, seven (7) variables were associated with MA use. Then, we have

modified the relevant context and added information in the discussion part. We thank you so much for these great suggestions. Please see page details in Table 5, and page 22, lines 3-15.

Thank you,

TK

Tawatchai Apidehckul
