# Supplementary material for: Factors influencing methamphetamine use among Lahu hill tribe youth in Chiang Rai, Thailand: A case‒control study
Source: PLoS One. 2026 Mar 12;21(3):e0344775. doi: 10.1371/journal.pone.0344775 (PMC12981475; doi:10.1371/journal.pone.0344775)
Supplement: Attachment — Submitted filename: MA_Reviewers_commenst_auresp_3.pdf [file pone.0344775.s008.pdf]

Response to reviewers comments

Dear Editor and reviewers,

Thank you very much for the opportunity to revise again with very clear point for improvement. We have re-analysis and revised the paper as suggested. All references have been double checked to ensure that are align with the journal requirement. Please see the new submitted paper and very hope you happy this version.

Thank you,

TK

Editor

Please review your reference list to ensure that it is complete and correct. If you have cited papers that have been retracted, please include the rationale for doing so in the manuscript text, or remove these references and replace them with relevant current references. Any changes to the reference list should be mentioned in the rebuttal letter that accompanies your revised manuscript. If you need to cite a retracted article, indicate the article's retracted status in the References list and also include a citation and full reference for the retraction notice.

:Thank you very much, we have carefully checked all references before this submission

Reviewer #2: Thanks for taking care of the minor edits.

With apology concerning the regression model in Table 5 - one more run please:

The first revision regression model included is Cigarette Smoking with a confidence interval of 2.34-25.62 (p-value <0.001*)

in the second revision the regression model included two overlapping variables concerning smoking.

Cigarette Smoking: with a confidence interval of 11.57-73.612 (p-value <0.001*)
Cigarette Accessibility: with a confidence interval of 0.25-229.55 (p-value 0.241)

While Cigarette Smoking is significant in both the first and second revision, the second revision Cigarette Smoking has a much broader CI than the first revision while the overlapping variable Cigarette Accessibility has a massively large CI range 0.25-229.55. Likely co-linearity again.

Please rerun the regression model for Table 5 with either Cigarette Smoking or Cigarette Accessibility alone but not both. One could try each one separately for interest. My hunch is either will be significant and the CI range much narrower.

: We have re-run a final model again with removed cigarette accessibility out of the final model. The range of CI of cigarette smoking was narrower than 2nd revised. All information has been revised and improved accordingly. Please see in page.2, lines 3-13, page 22 lines 3-15, and table 5 in page 23-24.

Thank you,

TK

Tawatchai Apidehckul
