# Supplementary material for: Factors influencing methamphetamine use among Lahu hill tribe youth in Chiang Rai, Thailand: A case‒control study
Source: PLoS One. 2026 Mar 12;21(3):e0344775. doi: 10.1371/journal.pone.0344775 (PMC12981475; doi:10.1371/journal.pone.0344775)
Supplement: Attachment — Submitted filename: PONE-D-25-27632 review.docx [file pone.0344775.s004.docx]

PONE-D-25-27632

Factors influencing methamphetamine use among Lahu hill tribe youth Chiang Rai, Thailand: A case‒control study

- In the following page and line numbers will be given as: page.#
  The printed page in the file will be used for the body of the text for page number.
- Edit suggestions (some stylistic, some substantive) will be given as: a => b (change a to b). [my explanations as to why appear in square brackets]

Review

This research investigates factors influencing methamphetamine use among Lahu hill tribe youth in norther Thailand using a case-control study with 136 cases (using) and 272 controls (not using), mostly male (79.4%) across four districts of Chiang Rai Province. It is an important topic.

Various predictors are tested for significance between cases and controls and a regression model employed thereafter. It is not clear why the variables were specifically chosen in the logistic regression given there are many that are significant in the univariate comparison between cases and controls. (One can’t include everything, but articulate why particular ones are chosen. Perhaps one his highly correlated with others, so it stands as a proxy for several – for example the similar significance of use of Twitter (X) and Instagram by controls (about 1 in 5) but much less by cases (p <0.001).

Several predictors were identified as significant in the model, but the confidence intervals are exceedingly wide around the estimate (for example an odds ratio of 6.40 for No schooling had a 95% CI of (1.12, 34.17) and Heroin Use had an odds ratio of 26.85 and 95% CI of (4.17, 172.95).

From a distance, the wide CI’s could be due to two or more predictors in the model having high correlation resulting in multicollinearity which will inflate the confidence intervals. Where high correlation between two predictors is the case, one predictor may more or less stand as a proxy also for the other giving justification for eliminating one from the model.

Given this possibility the authors should run a correlation matrix for the possible predictors in the model and check whether a correlation of 0.7 or more exists between two or more predictors. If logically related one can drop one. This should tighten the confidence intervals. One could run it with one and then the other to see how each impacts the other variable results as well – without too much fishing. Also one variable might be more important for the context thematically which might inform which to keep even if it is slightly less predictive. It may be that two logically unrelated predictors have high correlation by chance, which leaves one a choice on whether or not to eliminate one. If both being in inflates the Cis, then one should likely eliminate one of the predictors, noting their correlation in any case.

Whatever model is settled on, do mention the predictor that was dropped and its correlation level with the one kept if a reader might ask why a seemingly important variable found significant in a simple comparison between cases and controls was not in the multivariable model.

- Insert a table with AOR, 95%CI, p-values only for the multivariable logistic regression results where all of the included variables and results are together rather than results in the long list of univariate results separated far from each other in the long Table 4 (Factors, pages 16-24). That will make it much easier to read, edit, and comment on the logistic regression predictor selections and results.
- Also move Variables [Lines 5.25-28] on variables in the multivariate logistic regression to just before the new table after Table 4 with only with regression results. Note that there are many independent variables (possible predictors) that are significant but only a subset are included in the model. State how were they selected for the model (for example no social media variable was included although some are very significant univariately). See note above also concerning collinearity.
- When “controlling for age” in the multivariable model, is age the actual age of the person in years or the categorical Age variable listed as 15-19 and 20-24 years? (If actual age was there a coefficient and significance for the change in the log-odds of the outcome for each year above age 15; if categorical, was there an AOR for the variable?)
- Concerning Data Availability “No – some restrictions will apply” – explain the restrictions (what and why).
- On the Table called Factors covering pages 17-24 the percentages in parentheses should sum to be 100% vertically, not horizontally. That most are about 1/3 to 2/3 horizontally is due to the fact that there are only half as many cases as controls. Given different denominators 33.3 and 66.7 horizontally actually means 50% of cases and 50% of controls, which would show as 50.0 and 50.0 if summed vertically. The percentages for all Cases and Controls in the table have to be summed vertically.
- Page 27: A section labeled “Limitations” needs to be added after the Discussion. How generalizable are the results among all Lahu youth? For example the cases all have been in a treatment program but some MA users are not in a treatment program. Could there be differences between the MA users in treatment and those not reaching a treatment program? (How does one get into a treatment program?) Would one expect similar results in other hill tribe groups? Do the results compare with studies of other hill tribe groups (if there are such studies)? What are some limitations and advantages of Case-Control design in general?

**Abstract**

Background:

- 1.23 [page1.line23]: social insecurity => social security
  [ impact on “health” before is a positive term, so impact on security keeps the second consistent]
- 1.24-25: [are the Lahu the second largest hill tribe in Thailand/northern Thailand? Or are Lahu youth identified as the second largest marginalized subpopulation within the Lahu tribe? If so which is the largest hill tribe marginalized population? Clarify.]
- [Perhaps:] 1.25: population => subpopulation

Results:

- [see comment above about large confidence intervals as indicating possible multicollinearity from correlated predictors in the logistic regression model – rerun model after if finding this to be the case and eliminating a predictor]
- 2.2: “After controlling for age”
  [is actual age included as a continuous variable, is the two-level categorical variable Age (15-19, 20-24) used? is there a coefficient and significance related to Age in the regression model? The univariate comparison for age as a categorical variable was not significant.]

Conclusions: [Good]

Keywords: [Good]

Introduction:

3.9-10: part of the route of MA import, which is imported from neighboring countries,
 => part of the route of MA import from neighboring countries, then

3.11: and being => or being

3.13: uneconomic => subsistence

3.16: live under => live below	[just sounds a little better]

3.18: including health care services
 => including health care services, because [tell why not granted a Thai ID card]

3.21: to MA use, => for MA use,

3.22-23: This study aimed to => The present study aims to

Methods:

3.32: case-control => case-control study

4.1: the odds ratio from the previous study => the ratio from a previous study
 [ratio of controls to cases not odds-ratio? … it sounds like a simple ratio.]

4.2: a total of 136 cases and 272 controls
 => a total of 136 cases and 272 controls (a control/case ratio of 2.0)

4.8: been recorded in the MA treatment program as a client.
 [Could Lahu MA-using youth recorded in a MA treatment program differ from Lahu MA-using youth who never make it to a treatment program? Mention this possibility or address this in the Discussion or under Limitations.]

4.25: gender with three options for their response: male, female, or other
 [no respondent indicated ‘other’ for Gender in Table 1 on page 7. In the text ‘sex’ is used as well as gender. I would use only use ‘gender’ to be consistent with the questionnaire unless meaning biological sex exactly and not social/psychological identification. Also in Thai language is there a difference between “sex” and “gender” in the word used in the questionnaire? Among Lahu people generally (in contrast to academic researchers) “gender” may simply be understood as biological sex.

4.26: social media sue => social media use

5.25-28 [Section on Variables … comment above on moving these lines to just before a table with multivariate logistic regression results collected together in one table only with selected predictors.]

5.29-6.17 Recruitment procedure [good section on Recruitment procedure]

6.18-27 Statistical analysis [Good section – other comments on variable selection elsewhere.]
Note that Pseudo R-squared of logistic regression does not indicate proportion of variation of the dependent variable explained by the independent predictors as is the case of R-squared for linear regression. Pseudo R-squared in logistic regression context indicates the relative improvement in model fit compared to a baseline model (often a null model without predictors but only an intercept – that is the overall proportion in the data of the dependent variable being = 1 (which is trivial in a Case-Control set up where the cases = 1 and comprise one-third of the data by design as 2 controls = 0 have been included for every case such that controls =0 are two-thirds of the data).
For the Hosmer-Lemeshow test a high p-value is good (better fit) and a low p-value indicates a bad fit. This test, which uses a chi-squared approach, has been shown to be sensitive to the number of data partitions it uses (generally 10 in most software packages by default). A different number of partitions gives a different significance level for the same data. For this reason some researchers advise against using it although it is used in SPSS. It does provide some indication of goodness of fit of the model.

- 6.28-7.2: Ethical approval and consent to participate [Good section]
- 7.3: Results
- 7.13-9.1: Table 1 Comparisons of general characteristics between case and control [Table is OK]
- 9.7: [New paragraph - indent]
- 9.7: Regarding Substance use behaviors => Regarding Substance use behaviors in Table 2 below,
- 9.15-16: Facebook (78.2%) and Line (69.9%) were the most commonly used social media platforms.
  => Facebook (78.2%) and Line (69.9%) were the most commonly used social media platforms, but use levels were not significantly different between cases and controls.
- 9.16-17: sharing personal challenges with family members (p value<0.001)
   [move to bottom of paragraph]
- 9.16-17: sharing personal challenges with family members (p value<0.001)
   => sharing personal challenges with family members was stark in contrast with only 36.0% of cases doing so compared to 62.5% of controls (p value<0.001) – cases were much more isolated from family in communication.
- 9.17-18: using Instagram (p value<0.001), and using Twitter (p-value<0.001) (Table 2).
  => While about 1 in 5 controls used Twitter (X) and Instagram, cases used both much less (2.9% and 5.1%) both with p-value<0.001 (Table 2).
- For long tables, see if there is a box for repeating the table column labels at the top of each page if the table covers multiple pages.
- 12.2: Two-thirds (71.6%) of the participants had their parents living together.
  [Move this to Page 7 for discussion of Table 1 which has this variable.]

  Two-thirds (71.6%) of the participants had their parents living together.

=> The parents of cases lived together 18% less than controls (p<.001).

- 12.2: Half of => In Table 3 below, half of
- 12.16 (Table 3). => Cases had much higher relative access to cannabis, opium, and heroin than Controls (all p<.001).
- Page 13: [note “Having been persuaded by friend to use MA” has 9.6% of Controls saying “Yes” which should be 0% by the inclusion criteria. Perhaps the 9.6% of cases indicates that a friend attempted to persuade them but did not succeed.]
- 15.11: [How were the predictors selected for inclusion in the multiple logistic regression model given so many significant univariate variables were not included?]
- 15.11: [Is age the actual age in years or the two-level categorical variable from Table 1? Did age have a coefficient or odds ratio and a significance level?]
- 15.11-15.22: [See comment above about inflated confidence intervals (CI’s) and the need to check for collinearity and rerunning model with collinearity reduced.]
- Page 16: [Table 4 should start on this page beneath the heading. Almost blank page.]
- [For Table 4 AOR, 95%CI, p-value for the multivariate logistic regression can be gathered into a separate table for the model which is much more easy to read.]
- Pages 17-23: [The variable %’s in parentheses are now summed horizontally for each Factor in Table 4. These must be summed vertically to sum to 100%. Horizontally the cases are mostly about 1/3 and controls about 2/3 as that is the proportion of the design.]
- Page 24: The significance note should appear on Page 23 under the end of the table.

Page 25: Discussion

- 25.2: use, including => use including	[delete comma]
- 25.3: high knowledge of MA and control,
   => high knowledge of MA prevention and control,
- 25.3: and being => being
- 25.13: due to their health literacy => due to their health literacy being low
- 25.15: compulsory => necessary
- 25.16: Those => Seemingly paradoxically, those
- 25.24: prevention and control.
   => prevention and control. MA users not attending such clinics may also have a much lower level of knowledge as well.
- 25.27-28: smoking hill tribes, people living => hill tribes, people smoking living
- 25.28: did not. => did not smoke.
- 26.16: school. We also found that having no family member to share a personal challenge was true for 2/3 of the cases compared to 1/3 of the controls.
- Lines 26.23-31 [move to 7section titled “Limitations” perhaps after 27.8 or before Conclusion at 26.32]
- 27.1: organizational layers => structural layers
- [Insert blank line after 27.8]

Page 27: A section labeled “Limitations” needs to be added after the Discussion. Move Lines 26.23-31 under Limitations.

- 27.12: my => our
- 27.14: village head => village heads
- 27:14: participant => participants
- 27.18: P Phootawan => Phootawan

References [good]

- 30.2: thai => Thai
- 30.10: Lahu ethnical, Thailand => [Lahu ethnicity, Thailand]
   [square brackets for title as original is in Thai]

%%%%%%%%%%%%%%%%%%%%%%%%%%%%%%%%%%%%%%%%%%%

Thank you for the opportunity to review this important research.
