# Supplementary material for: Factors influencing methamphetamine use among Lahu hill tribe youth in Chiang Rai, Thailand: A case‒control study
Source: PLoS One. 2026 Mar 12;21(3):e0344775. doi: 10.1371/journal.pone.0344775 (PMC12981475; doi:10.1371/journal.pone.0344775)
Supplement: Attachment — Submitted filename: Respone to reviewer comments .pdf [file pone.0344775.s006.pdf]

Response to editor and reviewers comments

PONE-D-25-27632
Factors influencing methamphetamine use among Lahu hill tribe youth Chiang Rai, Thailand: A case–control study
PLOS ONE

Dear Dr. Apidechkul,

Thank you for submitting your manuscript to PLOS ONE. After careful consideration, we feel that it has merit but does not fully meet PLOS ONE's publication criteria as it currently stands. Therefore, we invite you to submit a revised version of the manuscript that addresses the points raised during the review process.

==============================
Please take into account each of the referee suggestions and revise accordingly. Looking forward to your revised version!

==============================

Please submit your revised manuscript by Aug 11 2025 11:59PM. If you will need more time than this to complete your revisions, please reply to this message or contact the journal office at plosone@plos.org. When you're ready to submit your revision, log on to https://www.editorialmanager.com/pone/ and select the 'Submissions Needing Revision' folder to locate your manuscript file.

Please include the following items when submitting your revised manuscript:

- A rebuttal letter that responds to each point raised by the academic editor and reviewer(s). You should upload this letter as a separate file labeled 'Response to Reviewers'.
- A marked-up copy of your manuscript that highlights changes made to the original version. You should upload this as a separate file labeled 'Revised Manuscript with Track Changes'.
- An unmarked version of your revised paper without tracked changes. You should upload this as a separate file labeled 'Manuscript'.

If you would like to make changes to your financial disclosure, please include your updated statement in your cover letter. Guidelines for resubmitting your figure files are available below the reviewer comments at the end of this letter.

If applicable, we recommend that you deposit your laboratory protocols in protocols.io to enhance the reproducibility of your results. Protocols.io assigns your protocol its own identifier (DOI) so that it can be cited independently in the future. For instructions see: https://journals.plos.org/plosone/s/submission-guidelines#loc-laboratory-protocols.

Additionally, PLOS ONE offers an option for publishing peer-reviewed Lab Protocol articles, which describe protocols hosted on protocols.io. Read more information on sharing protocols at https://plos.org/protocols?utm_medium=editorial-email&utm_source=authorletters&utm_campaign=protocols.

We look forward to receiving your revised manuscript.

Kind regards,

Rining Wei, PhD
Academic Editor
PLOS ONE

Journal Requirements:

When submitting your revision, we need you to address these additional requirements.

1. Please ensure that your manuscript meets PLOS ONE's style requirements, including those for file naming. The PLOS ONE style templates can be found at https://journals.plos.org/plosone/s/file?id=wjVg/PLOSOne_formatting_sample_main_body.pdf and https://journals.plos.org/plosone/s/file?id=ba62/PLOSOne_formatting_sample_title_authors_affiliations.pdf

: Thank you, we have double checked before submission.

2. Please include a complete copy of PLOS' questionnaire on inclusivity in global research in your revised manuscript. Our policy for research in this area aims to improve transparency in the reporting of research performed outside of researchers' own country or community. The policy applies to researchers who have travelled to a different country to conduct research, research with Indigenous populations or their lands, and research on cultural artefacts. The questionnaire can also be requested at the journal's discretion for any other submissions, even if these conditions are not met. Please find more information on the policy and a link to download a blank copy of the questionnaire here: https://journals.plos.org/plosone/s/best-practices-in-research-reporting. Please upload a completed version of your questionnaire as Supporting Information when you resubmit your manuscript.

: Thank you, we have submitted the STROBE into the system.

3. We note that you have indicated that there are restrictions to data sharing for this study. For studies involving human research participant data or other sensitive data, we encourage authors to share de-identified or anonymized data. However, when data cannot be publicly shared for ethical reasons, we allow authors to make their data sets available upon request. For information on unacceptable data access restrictions, please see http://journals.plos.org/plosone/s/data-availability#loc-unacceptable-data-access-restrictions.

Before we proceed with your manuscript, please address the following prompts:

a) If there are ethical or legal restrictions on sharing a de-identified data set, please explain them in detail (e.g., data contain potentially identifying or sensitive patient information, data are owned by a third-party organization, etc.) and who has imposed them (e.g., a Research Ethics Committee or Institutional Review Board, etc.). Please also provide contact information for a data access committee, ethics committee, or other institutional body to which data requests may be sent.

b) If there are no restrictions, please upload the minimal anonymized data set necessary to replicate your study findings to a stable, public repository and provide us with the relevant URLs, DOIs, or accession numbers. Please see http://www.bmj.com/content/340/bmj.c181.long for guidelines on how to de-identify and prepare clinical data for publication. For a list of recommended repositories, please see https://journals.plos.org/plosone/s/recommended-repositories. You also have the option of uploading the data as Supporting Information files, but we would recommend depositing data directly to a data repository if possible.

Please update your Data Availability statement in the submission form accordingly.

Since the data includes several sensitive issues that might be against ethical issue, we could provide the data. However, data could be requested from the corresponding author in some parts, such as general information.

4. In the online submission form, you indicated that data are available in a reasonable request to the corresponding author.

Yes, with reasonable requests to the corresponding authors, general information about participants could be accepted.

All PLOS journals now require all data underlying the findings described in their manuscript to be freely available to other researchers, either 1. In a public repository, 2. Within the manuscript itself, or 3. Uploaded as supplementary information.
This policy applies to all data except where public deposition would breach compliance with the protocol approved by your research ethics board. If your data cannot be made publicly available for ethical or legal reasons (e.g., public availability would compromise

patient privacy), please explain your reasons on resubmission and your exemption request will be escalated for approval.

5. Please include captions for your Supporting Information files at the end of your manuscript, and update any in-text citations to match accordingly. Please see our Supporting Information guidelines for more information: http://journals.plos.org/plosone/s/supporting-information.

:Thank you,

6. Please update your submission to use the PLOS LaTeX template. The template and more information on our requirements for LaTeX submissions can be found at http://journals.plos.org/plosone/s/latex.

: Thank you

Additional Editor Comments :

Please take into account each of the referee suggestions and revise accordingly.

Comments to the Author

Reviewer #1: Dear authors,
Thank you for your important work addressing methamphetamine (MA) use among Lahu youth—a critical public health issue with significant societal implications. Your manuscript is a sound technical piece. However, you can still improve it more to convince the reader that your methods were rigor. Few more information about the methods and materials will do that for you :-). Several statistical findings on questionnaire validation data, pilot tests, sensitivity tests and VIF will enhance the availability of your data. Overall, your paper is well structured. But, further synthesis (e.g., merging repetitive points) and explicit limitations would bolster its impact. Therefore, please find the detailed review sheets for your further perusal and wishing you all the very best! I appreciate your dedication to this vital topic and thank you for the opportunity given to read and review your paper.

Review Report: Factors influencing methamphetamine use among Lahu hill tribe youth Chiang Rai, Thailand: A case–control study

Dear authors,

It is remarkable that you have clearly pointed out MA as a public health issue, and the Lahu youth population is vulnerable to that. Yet, I would like to clear some concerns and provide several comments to further enhance the understandability of the manuscript.

Abstract:

The abstract is well structured. However, there are some minor concerns from my side, which I might need a little clarification or a slight modification to the written abstract. Please address them accordingly:

1.Under the methods: "... The participants were recruited from...". Could you please clarify the sampling method here?

: We used a random method to recruit the sample. Please see page 1, line 31.

2.The authors stated, "A validated questionnaire was used to collect data, and multiple logistic regression was used to detect association factors at α = 0.05." The use of the term "detect association factors at α = 0.05" is understandable. However, to make it technically sound, would you consider rewording the statement to provide an idea like "...with statistical significance set at α = 0.05..."? Such a clear expression would avoid any ambiguity.

: Thank you for the comment. We agree with you and have revised accordingly. Please see page 1, line 34.

3. When I was referring to the data collection instrument of your study, I noticed that the expected outcomes of your study are not always binary. If the outcome measures are binary, it is technically accurate to use the "multiple logistic regression"; otherwise, it is recommended to use linear or ordinal regression for the data analysis. Please recheck your data analysis plan and supplement with related information in your methods section.

: Thank you for the comment. Our study outcome was whether to use AM or not, which was set at the beginning. Then we used the logistic regression instead of linear regression.

Introduction:

Informative approach in introducing the scope of your research area and the aims. You have mentioned the neurobiological effects of using MA and also the societal consequences. Special emphasis on the socio-economic disparities in the context of Thailand's geographical risks is an important fact.

4. However, the lines 11 to 19 do not create a bridge between the MA use with the socio- economic disparities that you have emphasised there, including the 'education'. Could you please elaborate on the association between the MA use and the mentioned

socio- economic factors in the lines? Or could you briefly mention why Lahu youth are at higher risk by referring to the socioeconomic factors and geographic isolation?

Thank you for the great comment. We have extended details in page 3, lines 18-20.

5. It is appreciated that you have cited sources from both the Thai-originated sources and the international sources. When considering the logical flow of your introduction, it can be guessed that you have focused first on bringing the readers' attention to the global scenario, then Thailand, then Chiang Rai in Thailand, and the Lahu youth. However, it would be better if you could also add a paragraph to emphasise the gaps in the literature, which should be a critical element of an introduction to a manuscript. Because, in your introduction, it was not explicitly stated why previous research was not sufficient, or why the previous research was not available in your area of interest as a controlled case study.

: Thank you for your excellent comment. We have added information in page 3, lines 24-28.

6.Lines 22 to 24: You mentioned the study objective. However, there was no justification in the introduction on why the case-control method was selected. Could you briefly mention why you have selected the case-control study design? It is highly recommended to clarify/state the hypotheses rather than hinting it in a descriptive way through the research aim. Please consider modifying the way you report the aims by giving more focus into your study's hypotheses.

: Thank you for the comment. We totally agree with your comment that to guide the direction to readers, we need a hypothesis. Please see page 4, lines 3-5.

7.It is suggested to use your statements in lines 20-22 in the discussion section rather than in the introduction section.

: Thank you for the comment. With the context of the existing sentence focused on general discussion, we have modified the paragraph to be the basis of the idea to raise the study objective in this paragraph. Thank you very much. Please see lines page 3, 24-29,

8.Because your overall conclusion is to emphasise the need for "effective interventions and changes in policy" the introduction must expand a bit its concluding remarks by supplementing information about the policy relevance of this study.

: Thank you for such a great idea. We have extended the information on page 3, lines 29-30.

Materials and Methods:

The methods section is satisfactorily planned. However, the following comments/ questions will help you to increase the readability and broaden the understanding of your study for readers with less understanding of statistical methods. Please consider modifying your writing based on the following:

9.Line 27: The same comment (comment 3 of the introduction) applies here. You may address it under study design.

: Thank you. Please see the information added on page 3, lines 28-30.

10.Lines 29 to 30: Please consider using full sentences. Could you rewrite for better readability?

: Thank you for the comment. It is revised and completed. Please see page 4, lines 3-5

11.The sample size was calculated appropriately. However, to ensure expanded readability, it is better if you could add notes for the mentioned variables/constants/functions/symbols in the equation. This would help readers who may not have a wider understanding of equation-related statistical symbolic expressions.

: Thank you, we have added information in all notes used.

12. Line 10 onwards: To enhance the objective verification, could you mention whether there were complementary self-reports compared to the urine test results? If the results were not complementary, what was the next step?

: Thank you for the great comment. We have revised the sentences in lines 17-21 on page 4 to ensure that the information has been cleared for readers. Individuals who responded to any of the three methods with "Yes" were assumed to be a case (MA user).

13.The triple triple-check method is obviously a strong method to ensure the selection validity of your sample. Yet, despite the triple-check, there might still be social desirability bias among the controls as the MA use can be under-reported because of its stigma. Could you please try considering this as a fact when addressing the previous comment, too?

Thank you. We do know that with the conditions of illegal MA use in Thailand and also social desirability bias, which could be either a higher or lower possibility to say "yes" or "no" for some people, but it is the best way that we could do at the moment. We have put our limitations of the study on page 24, lines 17-32. Please also see my response to question No.14.

14.Also, the controls from the same village might share unmeasured confounders such as access to the MA. How have you addressed this as a fact?

: We selected cases and controls randomly after being classified by the triple method, and before the interview, they did not know about the process of identifying/assessing, including the questions asked, which are common to a case-control study. We did not have any special techniques implemented. We understand that if we worked as an intervention study, participants might share something about the project implemented.

15.If I have understood your inclusion and exclusion criteria for the 'cases' accurately, there is a hint of recall bias on self-reported MA use, as there is an absence of a biochemical verification. This point is recommended to address in the methods section or as a limitation in the discussion section.

: Since MA use is a big issue for any person, recall bias might not happen. However, it's possible to say "No' even if someone has used it. Then the triple method was created to use for identifying cases and controls. However, we don't know that it is not a perfect method, then we have clarified in the limitation page 24, lines 17-32.

16.Line 21: The claim "a validated questionnaire..." must be reported cautiously. The explained process does not hint at a full validation process of the questionnaire. Please consider this a critical comment.

: Thank you for your critical comment. We have provided information on how we improved our questionnaire's quality on page 5, lines 27-33, and page 6, lines 1-13. Thank you so much for your valuable comment at this point.

17.Line 22: Please cite the literature that you have referred to when developing the question items. a. "discussion with experts and local people..." How did you use the discussion findings to develop the questionnaire? Please explain in brief.

: We used references 1, 8, 11, 25 as the major papers to develop our tool, and had added properly. Discussion with experts and local people, we discussed with two nurses who were working in the amphetamine cessation clinic at Chiang Rai Prachanukroh Regional hospital located in Chiang Rai Province, and four community people (two village headmen and two village health volunteers), see details in page 5, lines 30-34, and page 6, lines 1-7.

18. It is not very clear how you have analysed the responses for the question related to the social media lists (binary vs. frequency?). Could you please explain? It is better to provide more information on your scoring system when managing the data.

: The question asking about social media use is presented in question no.1.13 of the questionnaire. It provided several options of media platforms, and the response just checked the box that they are using. The outcome is "use" for the checked box, and "not use" for the uncheck box. Please see page 6, lines 13-14.

19. In the 'knowledge section,' you have combined 'no' and 'not sure' into the same score (= 0).

: So sorry for the mistake. The answers on: yes, no, and not sure were originally created in their original form; however, after piloting, we changed them into “correct”, “incorrect”, and “unknown”. The details are presented on page 6, lines 21-25.

20. In the ‘attitude section’, you have scored neutral responses as 1. This might mask the true attitude of the respondent as the Likert scale is a 5-point one. Therefore, please clarify your scoring methodology and state if any sensitivity analysis was considered to ensure the rigour of the scoring methodology.

:Thank you for the comment. We had tried our best to improve this section, particularly adjusting the scoring method using the ref no.29-31. The Cronbach alpha analysis was found at 83.0%.

21. Line 19: “The questionnaire was determined to be valid by three external experts in the field”. Could you mention under what criteria the questionnaire was considered as validated?

This section has been improved with details. Please see page 7, lines 7-10.

22. Please briefly mention the background of the experts.

There were three experts: one professional psychologist and one nurse who were working at the MA cessation clinic, and one healthcare provider working in a health-promoting hospital. Please see details on page 7, lines 7-10.

23. Did any of the pilot study results influence you to revise the questionnaire?

: Yes, there were eleven (11) points that need to be revised during the pilot, see details on page 7, lines 17-28.

24.a. Is there any significant methodological outcome that you witnessed through the pilot study?

: We found that asking the questions on MA was very sensitive to the people in the villages. It needed to provide essential information to the participants to make them understand the purpose of the study, and get their willingness to provide information. This was not over our expectations before implementing the project, and we were very careful during data collection to make sure that we got accurate information as much as possible.

25.b. And for what exact purpose did you administer the pilot test?

: Piloting was aimed to improve both the validity and reliability of the questionnaire, and we had achieved both aims at the end.

26.Both under the ‘study sample’ and the ‘recruitment procedure’, there is some overlapping information on the triple-check method. Please consider revising.

: Thank you, it’s improved.

27.There is some clarity to be made on the variable selection, as this was not highlighted/connected well throughout the “introduction” section. Could you please explain the rationale for including the variables if the mentioned variables were chosen a priori based on theory or empirical univariate results?

: Since we have many essential variables in the study, we decided to use the stepwise method at a selection of 0.2 in the multivariable stage. With this method, all variables were included in the model, and treated whether included or excluded from the model at 0.2 during the analysis.

28.In the step-wise regression, did you pre-specify the key confounders in the model regardless of the significance? Please elaborate a little.

: At the final model of the multivariable analysis, we added “age” to control before interpretation. Please see revised details on page 8, lines 9-12.

29. Then you may explain whether you have proceeded step-wise for exploratory variables.

: Since the univariable logistic regression was used in the first step, we used the stepwise multivariable logistic regression in the final step. Using the stepwise method with many predicted variables, all variables were explored for their association again at 0.2, including and excluding the model. Please see revised details on page 7, lines 31-34 and page 8, lines 1-9.

30. Please briefly mention whether there was any correlation between variables such as education, occupation and income (VIF/tolerance).

: Thank you, we have added information on page 8, lines 2-7.

31. Could you tell how the continuous predictors were handled?

We have only “age” as continuous data, and it was categorized into two categories before being entered into the model.

32. Because the interpretations of odds/ratios can be affected, please mention how the knowledge/attitude scores were treated under the variable type.

: Thank you for the small but great comment. We have added information “Both knowledge and attitudes were categorized before entering into the analysis, which

could impact the final interpretation. However, this was minimized by the large sample size recruited into the study," on page 6, lines 26-33, and page 7, line 1-4.

33. Were there any missing data? If 'yes', how did you handle those?

: No, there was no missing data because we had carefully revisited it again on the questionnaire before leaving the participants.

34 **Results**: The way you decided to report the results indicates the structural planning of reporting the results. However, you may still reduce the redundancies in the text by streamlining the text and the table. It is suggested to refer the readers to tables rather than repeating a lot of context through a text, which can be easily grasped through the table figures.

: Thank you for the comment, and we have revised all the result sections to reduce the information as much as possible, but maintain the understanding for readers.

35.There were inconsistencies noted in how the '*p*' was reported in terms of expressing it through the words. You may reconsider whether you use the term "p value" or "α" based on the referencing and reporting guidelines given by the journal.

: Thank you, we revised it to $p\text{-}value \leq 0.05$

36.Could you please include the top univariate associations in the text with *p*-values/ ORs rather than forcing the readers to consult the tables? Univariate statistics play a crucial role in your study. Hence, much emphasis should be on this, rather than allocating more space in the text to the descriptive data explanations. Please ignore if you have addressed this comment through the methods section under statistical analysis. If note, please address:

: Yes, all significant variables are presented in the text. However, after putting p-values or ORs in each significant variable and its sub-categories, they need a large space, so we decided not to present the details of either p-values or ORs.

37. Why are the other variables, except the age, absent in the multivariate model? Did you drop those in a step-wise regression model? Please mention the *p*-value if the missing variables were statistically not significant.

: Yes, the step-wise logistic regression model presents only the significant variables at the end. Please see page 8, lines 9-12.

Discussion:

The discussion section has many strong points. You have directly addressed the key findings with citations to build a discussion. The most significant aspect is that you discussed the Lahu-specific vulnerabilities. This will be an interesting section from a

global point of view if you could compare Lahu findings in-depth to other Thai hill tribes or global indigenous youth. Engaging the counterintuitive results and transition logic on substance use would catch the attention of the readers. However, you may still add more depth to the discussion by paying attention to the following aspects/comments/suggestions:

38. You may still discuss peer/family pressure (given the aOR = 13.79) in depth. You may discuss how the extreme risk of peer persuasion suggests MA use due to its communal living or limited alternative recreations. You may refer to social learning theory or cultural connaissance theory if you need more insights.

: Thank you for your great comment on this point. We have extended the information at this point using the social learning theory.

39.“...cases learned from clinics...” Did cases score higher because they overestimated their knowledge? Please discuss. Because there may be a misclassification bias or a knowledge-behaviour gap.

: Thank you for the comment. We have discussed multiple points in this issue, and we are sure that misclassification will be less likely to occur, but we could even use the triple method. However, we did not concern knowledge behavior gap between cases and controls in our study. Please see page 23, lines 24-25.

40.It is great that you have mentioned the remote village access to MA. But could you discuss any recommendations based on any available national action plan on MA, on any micro-level strategies that Thailand proposed?

: Thank you, please page 25, lines 5-9

41.There are repetitions in the highlights of smoking and heroin. Could you synthesise your repeated claims into one paragraph (you may emphasise local drug sequencing patterns)?

: Thank you for the excellent comment. We have revised and re written the entire section. Please see page 23, lines 16-30.

42.Peer influence and accessibility related facts are repetitively discussed in the discussion section. Could you consider merging the facts into one subsection with a deeper synthesis?

: Thank you for the comment. After discussing among the authors, with the difference between these two factors, separating the presentation could be easier for the reader. Then, we have maintained their original form but extended it with more details. We do hope you understand us.

43.In the conclusion, please make sure to synthesise your novel contributions by mentioning how the Lahu tribe findings may differ from the other domestic or international hill tribes.

: Thank you for the comment. We have revised based on our findings, and have extended more information in this section. Definitely, we carefully conclude and avoid overclaiming the findings.

44.The policy implications that you suggest should be more specific, actionable steps rather than being vague (“special training programmes” ??? like what ?). Could you consider proposing specific models by referring to a previous successful intervention or programme models?

: Thank you for the comment. We proposed a special training to improve knowledge and attitudes to prevent the use of MA among the Lahu youths. Please see page 25, lines 6-9.

45.It would be great if you could acknowledge the limitations of the study design and the biases. Please acknowledge the limitations of your study and provide suggestions/directions for further research.

: Thank you, please see page 24, lines 17-32.

I hope that the comments/questions will improve the presentation of your valuable contribution and increase the capacity of dissemination of the found knowledge by you. Congratulations!

Thank you so much for so many excellent comments. We appreciate all your efforts and feedback on our paper leading to substantial improvement. We also very hope you happy on our revised version.

Reviewer #2: PONE-D-25-27632
Factors influencing methamphetamine use among Lahu hill tribe youth Chiang Rai, Thailand: A case–control study
• In the following page and line numbers will be given as: page.#
The printed page in the file will be used for the body of the text for page number.

• Edit suggestions (some stylistic, some substantive) will be given as: a => b (change a to b). [my explanations as to why appear in square brackets]

: Thank you for the clarifications.

Review

This research investigates factors influencing methamphetamine use among Lahu hill tribe youth in norther Thailand using a case-control study with 136 cases (using) and 272 controls (not using), mostly male (79.4%) across four districts of Chiang Rai Province. It is an important topic.

46.Various predictors are tested for significance between cases and controls and a regression model employed thereafter. It is not clear why the variables were specifically chosen in the logistic regression given there are many that are significant in the univariate comparison between cases and controls. (One can't include everything, but articulate why particular ones are chosen. Perhaps one his highly correlated with others, so it stands as a proxy for several – for example the similar significance of use of Twitter (X) and Instagram by controls (about 1 in 5) but much less by cases ($p < 0.001$).

Several predictors were identified as significant in the model, but the confidence intervals are exceedingly wide around the estimate (for example an odds ratio of 6.40 for No schooling had a 95% CI of (1.12, 34.17) and Heroin Use had an odds ratio of 26.85 and 95% CI of (4.17, 172.95).

From a distance, the wide CI's could be due to two or more predictors in the model having high correlation resulting in multicollinearity which will inflate the confidence intervals. Where high correlation between two predictors is the case, one predictor may more or less stand as a proxy also for the other giving justification for eliminating one from the model.

Given this possibility the authors should run a correlation matrix for the possible predictors in the model and check whether a correlation of 0.7 or more exists between two or more predictors. If logically related one can drop one. This should tighten the confidence intervals. One could run it with one and then the other to see how each impacts the other variable results as well – without too much fishing. Also one variable might be more important for the context thematically which might inform which to keep even if it is slightly less predictive. It may be that two logically unrelated predictors have high correlation by chance, which leaves one a choice on whether or not to eliminate one. If both being in inflates the Cis, then one should likely eliminate one of the predictors, noting their correlation in any case. Whatever model is settled on, do mention the predictor that was dropped and its correlation level with the one kept if a reader might ask why a seemingly important variable found significant in a simple comparison between cases and controls was not in the multivariable model.

Thank you for the great comment, which coincides with one of the feedback from the first reviewer on No.30. We had assessed the collinearity by correlation matrix, to detect high correlation among the variables before deciding to put them into the logistic regression. In this step, only a pair of cannabis accessibility and opium accessibility was found to have a correlation of $r=0.87$, so we maintained only opium accessibility in the final prediction model. Variance inflation factor (VIF) and tolerance were also tested,

while including all variables in the model. Please see details on page 8, lines 2-12.

For the wide CIs, we observed that there were small samples that fell into some cells, then it could make wide interval of the CIs.

47. Insert a table with AOR, 95%CI, p-values only for the multivariable logistic regression results where all of the included variables and results are together rather than results in the long list of univariate results separated far from each other in the long Table 4 (Factors, pages 16-24). That will make it much easier to read, edit, and comment on the logistic regression predictor selections and results. Also move Variables [Lines 5.25-28] on variables in the multivariate logistic regression to just before the new table after Table 4 with only with regression results. Note that there are many independent variables (possible predictors) that are significant but only a subset are included in the model. State how were they selected for the model (for example no social media variable was included although some are very significant univariately). See note above also concerning collinearity.

: Thank you for the comment. We have created Table 5 to present the multivariate analysis as suggested. Please see page 21-22.

:About the sub-variables in some of the independent variables, our purpose from the initial stage of the project was to explore the indirect behaviors relevant to MA use; however, details of sub-variables were found during the pilot. Then we put all sub-variables in the questionnaire and preliminarily assessed in univariate analysis. However, in the final model, we needed to keep a high power of the test due to limited sample size, then we used one key independent variable in the model. We are also thinking of working on a new project that focuses on social media use behaviors and substance use behavior in the future. Thank you so much for your insightful comment and we do hope you understand us.

48. When “controlling for age” in the multivariable model, is age the actual age of the person in years or the categorical Age variable listed as 15-19 and 20-24 years? (If actual age was there a coefficient and significance for the change in the log-odds of the outcome for each year above age 15; if categorical, was there an AOR for the variable?)

: We control age in categorical form in the final stage after running the step-wise logistic regression, then we did not put it into the table to reduce the confusion.

49. Concerning Data Availability “No – some restrictions will apply” – explain the restrictions (what and why).

: Because this study is about MA use in the hill tribe and stateless people, which are serious issues, we need to ask the purpose and level of data in anyone’s request.

50.On the Table called Factors covering pages 17-24 the percentages in parentheses should sum to be 100% vertically, not horizontally. That most are about 1/3 to 2/3 horizontally is due to the fact that there are only half as many cases as controls. Given different denominators 33.3 and 66.7 horizontally actually means 50% of cases and 50% of controls, which would show as 50.0 and 50.0 if summed vertically. The percentages for all Cases and Controls in the table have to be summed vertically.

:Thank you for the comment. We have revised the percentages presented in Table 4 in vertical form.

51.Page 27: A section labeled "Limitations" needs to be added after the Discussion. How generalizable are the results among all Lahu youth? For example the cases all have been in a treatment program but some MA users are not in a treatment program. Could there be differences between the MA users in treatment and those not reaching a treatment program? (How does one get into a treatment program?) Would one expect similar results in other hill tribe groups? Do the results compare with studies of other hill tribe groups (if there are such studies)? What are some limitations and advantages of Case-Control design in general?

; Thank you for the great comments. We have extended our limitation to generalization, characteristics of cases that were selected from a clinic, and the advantage of a case-control. Please see page 24, lines 17-32.

Abstract
Background:
52. 1.23 [page1.line23]: social insecurity => social security
[impact on "health" before is a positive term, so impact on security keeps the second consistent]

: It is improved.

53. 1.24-25: [are the Lahu the second largest hill tribe in Thailand/northern Thailand? Or are Lahu youth identified as the second largest marginalized subpopulation within the Lahu tribe? If so which is the largest hill tribe marginalized population? Clarify.]

: Lahu is the second largest group of the hill tribe and stateless people after the Akha people. Lahu youths are also the second after the Akha. Please see page 3, lines 14-15.

54. [Perhaps:] 1.25: population => subpopulation

: It is improved.

Results:
55.[see comment above about large confidence intervals as indicating possible multicollinearity from correlated predictors in the logistic regression model – rerun model after if finding this to be the case and eliminating a predictor]

Thank you for the comment. We have re-examined the multicollinearity using the correlation matrix and re-run the model.

56. 2.2: "After controlling for age"
[is actual age included as a continuous variable, is the two-level categorical variable Age (15-19, 20-24) used? is there a coefficient and significance related to Age in the regression model? The univariate comparison for age as a categorical variable was not significant.]

: We used the categorical form in the model and did not present in the table as respone in question 48.

Conclusions: [Good]
Keywords: [Good]

Introduction:
57. 3.9-10: part of the route of MA import, which is imported from neighboring countries, => part of the route of MA import from neighboring countries, then

: It is improved.

58. 3.11: and being => or being

: It is improved.

59. 3.13: uneconomic => subsistence

: It is improved.

60. 3.16: live under => live below [just sounds a little better]

: It is improved.

61. 3.18: including health care services
=> including health care services, because [tell why not granted a Thai ID card]

: It is improved.

62. 3.21: to MA use, => for MA use,

: This sentence is replaced with the new one based on the reviewer no. 1 comment

63. 3.22-23: This study aimed to => The present study aims to

: It is improved.

Methods:
64. 3.32: case-control => case-control study

: It is improved.

65. 4.1: the odds ratio from the previous study => the ratio from a previous study
[ratio of controls to cases not odds-ratio? … it sounds like a simple ratio.]

: In this case, we mentioned the odds ratio reported from the previous study.

66. 4.2: a total of 136 cases and 272 controls
=> a total of 136 cases and 272 controls (a control/case ratio of 2.0)

: It is improved.

67. 4.8: been recorded in the MA treatment program as a client.
[Could Lahu MA-using youth recorded in a MA treatment program differ from Lahu MA-using youth who never make it to a treatment program? Mention this possibility or address this in the Discussion or under Limitations.]

: Thank you, we have extended the imitation in page 24, lines 17-24.

68. 4.25: gender with three options for their response: male, female, or other
[no respondent indicated ‘other’ for Gender in Table 1 on page 7. In the text ‘sex’ is used as well as gender. I would use only use ‘gender’ to be consistent with the questionnaire unless meaning biological sex exactly and not social/psychological identification. Also in Thai language is there a difference between “sex” and “gender” in the word used in the questionnaire? Among Lahu people generally (in contrast to academic researchers) “gender” may simply be understood as biological sex.

: Thank you for the notice and comment. We have now used gender to replace sex in the paper. In Thai, we have only one “sex” in general; however, in some circumstances, people might extend the context into two different senses: sex and gender.

69. 4.26: social media sue => social media use

: It is improved.

70. 5.25-28 [Section on Variables … comment above on moving these lines to just before a table with multivariate logistic regression results collected together in one table only with selected predictors.]

: Thank you for the great comment. After carefully looking into the sequence of the text body, we decided to move this section “Variable” to put before table 4 because both univariate and multivariate analyses are used to extend the thought of readers. We do hope you understand us. Please see 16, lines 1-6.

71. 5.29-6.17 Recruitment procedure [good section on Recruitment procedure]

: Thank you

72. 6.18-27 Statistical analysis [Good section – other comments on variable selection elsewhere.]

: Thank you, all comments have been revised

73. Note that Pseudo R-squared of logistic regression does not indicate proportion of variation of the dependent variable explained by the independent predictors as is the case of R-squared for linear regression. Pseudo R-squared in logistic regression context indicates the relative improvement in model fit compared to a baseline model (often a null model without predictors but only an intercept – that is the overall proportion in the data of the dependent variable being = 1 (which is trivial in a Case-Control set up where the cases = 1 and comprise one-third of the data by design as 2 controls = 0 have been included for every case such that controls =0 are two-thirds of the data).

For the Hosmer-Lemeshow test a high p-value is good (better fit) and a low p-value indicates a bad fit. This test, which uses a chi-squared approach, has been shown to be sensitive to the number of data partitions it uses (generally 10 in most software packages by default). A different number of partitions gives a different significance level for the same data. For this reason some researchers advise against using it although it is used in SPSS. It does provide some indication of goodness of fit of the model.

: Thank you so much for the valuable information.

74. 6.28-7.2: Ethical approval and consent to participate [Good section]

: Thank you

• 7.3: Results

75. 7.13-9.1: Table 1 Comparisons of general characteristics between case and control [Table is OK]

: Thank you

76. 9.7: [New paragraph - indent]

: Revised

77. 9.7: Regarding Substance use behaviors => Regarding Substance use behaviors in Table 2 below,

: It is improved.

78. 9.15-16: Facebook (78.2%) and Line (69.9%) were the most commonly used social media platforms.
=> Facebook (78.2%) and Line (69.9%) were the most commonly used social media platforms, but use levels were not significantly different between cases and controls.

: It is improved.

79. 9.16-17: sharing personal challenges with family members (p value<0.001) [move to bottom of paragraph]

: It is improved

80.9.16-17: sharing personal challenges with family members (p value<0.001)
=> sharing personal challenges with family members was stark in contrast with only 36.0% of cases doing so compared to 62.5% of controls (p value<0.001) – cases were much more isolated from family in communication.

: It is improved

81. 9.17-18: using Instagram (p value<0.001), and using Twitter (p-value<0.001) (Table 2).
=> While about 1 in 5 controls used Twitter (X) and Instagram, cases used both much less (2.9% and 5.1%) both with p-value<0.001 (Table 2).

: It is improved

82. For long tables, see if there is a box for repeating the table column labels at the top of each page if the table covers multiple pages.

: Thank you, all tables were checked to ensure their proper presentation, not repeating information.

83. 12.2: Two-thirds (71.6%) of the participants had their parents living together. [Move this to Page 7 for discussion of Table 1 which has this variable.]

: Thank you so much, it's moved to page 8, lines 27.

83. Two-thirds (71.6%) of the participants had their parents living together.
=> The parents of cases lived together 18% less than controls (p<.001).

: Thank you so much, it's moved to page 8, lines 27.

84. 12.2: Half of => In Table 3 below, half of

: It is improved

85. 12.16 (Table 3). => Cases had much higher relative access to cannabis, opium, and heroin than Controls (all p<.001).

: It is improved

86. Page 13: [note "Having been persuaded by friend to use MA" has 9.6% of Controls saying "Yes" which should be 0% by the inclusion criteria. Perhaps the 9.6% of cases indicates that a friend attempted to persuade them but did not succeed.]

: Yes, thank you for the comment.

87.15.11: [How were the predictors selected for inclusion in the multiple logistic regression model given so many significant univariate variables were not included?]

:We used the stepwise logistic regression; all variables were put into the model at the same time before interpretation.

88. 15.11: [Is age the actual age in years or the two-level categorical variable from Table 1? Did age have a coefficient or odds ratio and a significance level?]

: Age was categorized before being put into the model

89. 15.11-15.22: [See comment above about inflated confidence intervals (CI's) and the need to check for collinearity and rerunning model with collinearity reduced.]

: We have done all the steps as suggested to detect the collinearity

90. Page 16: [Table 4 should start on this page beneath the heading. Almost blank page.]

: It is rearranged properly.

91. [For Table 4 AOR, 95%CI, p-value for the multivariate logistic regression can be gathered into a separate table for the model which is much more easy to read.]

: Thank you, we have created new table, table 5.

92.Pages 17-23: [The variable %'s in parentheses are now summed horizontally for each Factor in Table 4. These must be summed vertically to sum to 100%. Horizontally the cases are mostly about 1/3 and controls about 2/3 as that is the proportion of the design.]

: All horizontal %'s are revised to vertical %'s, see table 4

93. Page 24: The significance note should appear on Page 23 under the end of the table.

: It is moved to the proper place/area under the table.

Page 25: Discussion
94. 25.2: use, including => use including [delete comma]

: It is improved

95. 25.3: high knowledge of MA and control,

=> high knowledge of MA prevention and control,
: It is improved

96. 25.3: and being => being

: It is improved

97. 25.13: due to their health literacy => due to their health literacy being low

: It is improved

98. 25.15: compulsory => necessary

: It is improved

99. 25.16: Those => Seemingly paradoxically, those

: It is improved

100: 25.24: prevention and control.
=> prevention and control. MA users not attending such clinics may also have a much lower level of knowledge as well.

: It is improved together with one more additional sentence added from the first reviewer's feedback.

101. 25.27-28: smoking hill tribes, people living => hill tribes, people smoking living

: The whole sentence is revised and improved.

102. 25.28: did not. => did not smoke.

: It is improved

103. 26.16: school. We also found that having no family member to share a personal challenge was true for 2/3 of the cases compared to 1/3 of the controls.

: Thank you very much, the sentence is added.

104. Lines 26.23-31 [move to section titled “Limitations” perhaps after 27.8 or before Conclusion at 26.32]

: They are moved after “Limitations”

105. 27.1: organizational layers => structural layers

: It is improved

106. [Insert blank line after 27.8]

: Thank you, it’s inserted

107. Page 27: A section labeled “Limitations” needs to be added after the Discussion. Move Lines 26.23-31 under Limitations.

: Thank you, it’s added

108. 27.12: my => our

: It is improved

109. 27.14: village head => village heads

: It is improved

110. 27:14: participant => participants

: It is improved

111. 27.18: P Phootawan => Phootawan

: It is improved

References [good]
112. 30.2: thai => Thai

: It is improved
113. 30.10: Lahu ethnical, Thailand => [Lahu ethnicity, Thailand]
[square brackets for title as original is in Thai]

: It is improved

Thank you for the opportunity to review this important research.

: We thank you so much for your comments/ feedback and also corrections along the entire paper, very very appreciated!!

Reviewer #3: I want to commend the authors for their work on this relevant topic. The research tackles a critical public health issue affecting underprivileged populations and demonstrates interesting innovations, such as the creative triple-check control verification method and rigorous objective biomarker validation—both of which advance the epidemiological science among marginalized communities. Please find below my suggestions and concerns, organized point-by-point.

Introduction:

114. I'm curious about the national identification card system in Thailand, as this emerges as such an important factor in your findings. It would be helpful to understand the circumstances under which Lahu community members might not receive national identification cards and the implications this has for accessing services.

: Yes, as mentioned in the paper, the ID card is used for access to all public services in Thailand. Those who do not grant the ID will be facing several barriers, including being charged for the service, particularly access to healthcare services.

115. Your introduction does an excellent job establishing the MA use challenges among Lahu youth. I wonder if you might consider sharing additional insights about other socio-cultural characteristics of this population? This could help readers like myself better understand the broader environment that contributes to these patterns.

: Thank you so much, we have added several information about the Lahu people. Please see paragraphs 3-4 in the introduction section.

Methods:

115. The sample size calculation references a "previous study" (Thailand narcotics control annual report 2023). While the calculation is provided, it would enhance clarity to briefly explain how this reference study relates to the current methodological approach and justify the assumptions used.

Because the information from the Thailand narcotics control annual report 2023 was the recent information about the Am use in this population in Thailand, we decided to use it as the reference for our sample size calculation.

117. For improved readability, consider reorganizing the methods section by placing "recruitment procedure" immediately after "study sample," potentially subdividing this into "inclusion criteria" and "recruitment procedures." This would maintain the logical flow of understanding sample construction.

Thank you so much for the great comment. We have moved the subtopic as suggested.

118. If possible, I believe a participant flow diagram would benefit overall comprehension for the case-control by showing the selection process clearly.

Since the selection of cases came from four hospitals (the MA clinics), while controls were drawn from several villages, we have tried to create the diagram, but it seems less meaningful. Moreover, with the ethics, we have tried to maximize the possibility of retracing the participants. Then we decided not to put the diagram in the manuscript, and we do hope you understand us.

119. Regarding case selection from MA clinics: could the authors clarify whether this refers to a single clinic or multiple facilities? Additional context about the clinic setting(s) would help readers understand the representativeness of the case population.

: Cases were recruited from four district hospital MA clinics: Mae Fah Luang, Mae Chan, Muang, and Mae Suai district hospitals. Please page 5, lines 8-10.

120.Your control selection process sounds very thorough. I am still unsure whether controls were drawn from the entire eligible Lahu community aged 15-24, or if there were additional selection considerations. You might also consider moving that first results paragraph (with the final sample sizes) into methods—it would really strengthen the transparency of your approach.

: Controls were randomly selected after checking by the triple method from all youths aged 15-24 years living in the selected case. Afterwards, gender-matched to all target controls who lived in the case's village was conducted and checked by a triple method before finally selecting the controls. Please see details in page 5, lines 10-15.

: Thank you for the recommendation to move the first paragraph of the result under the sample size calculation. The paragraph was moved.

121. While sex-matching is well-justified given known gender differences in MA use, could you elaborate on the decision not to match on other factors like age range? The rationale for matching choices is crucial in case-control design and would contribute to the methods description.

: We had seriously discussed matching other profiles between case and control at the beginning of the project. However, we concluded that only gender would be enough because the age interval of the target participants was too wide, and we could not control it in the model. Too many factors matching could make it difficult to find controls.

122. The authors write that “There were several independent variables or predictors in the study, e.g., age, sex, marital status, education, occupation, family income, knowledge of MA prevention and control, and attitudes toward MA prevention and control.” I would suggest that they either clearly define the variables rather than putting out examples of what may appear in the study. For instance, STROBE reporting guidelines (doi:10.1371/journal.pmed.0040296) suggest we “clearly define all outcomes, exposures, predictors, potential confounders, and effect modifiers”, and the authors do provide some comprehensive description for variables in the “research instruments” sections. However, it’s preferable to have an objective perspective on variables used in the study, with clear definitions if not previously done. I suggest the authors revise their instruments and variables section to avoid redundant content and explicitly – and intentionally – present the selected variables, their treatment and application (e.g. outcome, covariate, exposure).

: Thank you for the comment. We have followed the STROBE guidelines in this manuscript development. We provided this information under the sub-topic of variable in the method section; however, with the suggestion of Reviewer 2, it has been moved before Table 4. Please see details on page 16, lines 3-8.

123. I'm interested in your 15,000-baht household income threshold. Could you help us understand how this relates to Thai socioeconomic standards? I noticed you mention Thailand's national poverty line of $1,400/year per family in the introduction—connecting these would perhaps help.

This is because the salary for a recent graduate (average age 20-21 years) in Thailand is 15,000 baht per month, so we used it as the cutoff. However, the $1,400/year per family is the national poverty line.

124. I think there might be a small calculation issue in Table 1's household income row (the percentages appear to sum to 109%)—just wanted to flag this for your review.

: Thank you for the observation. Since we worked on this manuscript by all authors, and at some stage, someone deleted some data. We have revised/and double-checked the entire data set in the paper.

• Please clarify whether missing data analysis or sensitivity analyses were conducted. If not performed, this should be explicitly stated. If conducted, the patterns and handling methods would be valuable to report.

: After the participants completed the questionnaire, it was checked again by the researcher before leaving. After entry into Excel, the data were checked for missing data at the beginning before data analysis. This is the process of completing the data. Almost all data are completed except family income because the participants told us that they did not know exactly.

Results:
125. Consider streamlining the presentation by reducing repetitive "p value" references throughout the text.

: Thank you for the great comment. We have deleted all the p-values presented in the text. Readers could see details on the p-value from the table, except for some standalone values.

126. The final (adjusted) multivariate model controls only for age despite multiple significant univariate associations (education, occupation, income, family status). Could you discuss the rationale for this approach and potential implications for residual confounding?

: Adjusting for age in the model because from our literature review, we found that age was a strong confounder in MA use detection. While several factors were included from the literature review and from interviews with the people in the villages and health care providers who were working in the MA clinics, were collected from the participants. Even though a strong study design, a case-control study, was conducted, several factors were found to be associated at the univariate analysis, but did not find a significant association in the multivariate analysis, and had wide CIs. The majority could be because a few samples fell in some cells (due to many variables put into the model). Please see details in the limitation section, page 24.

127. Could you clarify whether you included age as a continuous variable or in categorical groups in your multivariate analysis? Just a small detail that would be helpful.

Age was categorized into two groups before being put into the model.

128. Given the cross-sectional measurement of exposures and outcomes, could you

address the temporal sequence challenges, particularly for variables like "knowledge of MA prevention and control"? This pattern suggests the knowledge was acquired through treatment programs after MA use initiation, rather than representing a causal pathway to use. For clarity, I would recommend the authors explicitly state that their counterintuitive findings regarding increased use associated with MA knowledge may be caused by reverse causation bias: knowledge came after MA use, not before.

: Thank you so much for the critical comment. Even though we used a case-control design in the study, for data collection by stressing the sequence of the exposures and outcome during the interview, the temporal sequence challenges were still an issue. We extended our discussion in page 23 lines 13-15.

129. Some effect sizes appear unusually large with very wide confidence intervals (e.g., heroin use: AOR 30.14, CI: 4.29-211.95; friend persuasion: AOR 13.79, CI: 3.80-50.05). These suggest either very small cell sizes or potential data quality issues. Could you provide the actual numbers in these cells and discuss the reliability of these estimates?

: Thank you very much for the comment on this issue, which is one of the concerns and notices of other reviewers as well.

130. Before concluding, I would also recommend the authors consider discussing study limitations more comprehensively, including potential selection biases, temporal sequence issues, and generalizability beyond this specific population.

: Thank you for the great comment, which is similar to other review comments. We have extended this information on our limitations. Please see page 24, lines 17-32, on the subhead “Limitations”.

Finally, we would like to thank all valuable comments from all three reviewers, which led to substantial improvement on our manuscript. We do really hope that yiu happy in this revied version.

Thank you,

TK

Tawatchai Apidehckul
