# Supplementary material for: Factors influencing methamphetamine use among Lahu hill tribe youth in Chiang Rai, Thailand: A case‒control study
Source: PLoS One. 2026 Mar 12;21(3):e0344775. doi: 10.1371/journal.pone.0344775 (PMC12981475; doi:10.1371/journal.pone.0344775)
Supplement: Peer review history

# Peer review: Decision Letter 0

**DOI:** 10.1371/journal.pone.0344775.r001

**Authors:** Rining Wei

27 Jun 2025

Dear Dr. Apidechkul,

Thank you for submitting your manuscript to PLOS ONE. After careful consideration, we feel that it has merit but does not fully meet PLOS ONE’s publication criteria as it currently stands. Therefore, we invite you to submit a revised version of the manuscript that addresses the points raised during the review process.

==============================

==============================

Please submit your revised manuscript by Aug 11 2025 11:59PM. If you will need more time than this to complete your revisions, please reply to this message or contact the journal office at plosone@plos.org . When you're ready to submit your revision, log on to https://www.editorialmanager.com/pone/ and select the 'Submissions Needing Revision' folder to locate your manuscript file.

- A rebuttal letter that responds to each point raised by the academic editor and reviewer(s). You should upload this letter as a separate file labeled 'Response to Reviewers'.
- A marked-up copy of your manuscript that highlights changes made to the original version. You should upload this as a separate file labeled 'Revised Manuscript with Track Changes'.
- An unmarked version of your revised paper without tracked changes. You should upload this as a separate file labeled 'Manuscript'.

If you would like to make changes to your financial disclosure, please include your updated statement in your cover letter. Guidelines for resubmitting your figure files are available below the reviewer comments at the end of this letter.

If applicable, we recommend that you deposit your laboratory protocols in protocols.io to enhance the reproducibility of your results. Protocols.io assigns your protocol its own identifier (DOI) so that it can be cited independently in the future. For instructions see: https://journals.plos.org/plosone/s/submission-guidelines#loc-laboratory-protocols . Additionally, PLOS ONE offers an option for publishing peer-reviewed Lab Protocol articles, which describe protocols hosted on protocols.io. Read more information on sharing protocols at https://plos.org/protocols?utm_medium=editorial-email&utm_source=authorletters&utm_campaign=protocols .

We look forward to receiving your revised manuscript.

Kind regards,

Rining Wei, PhD

Academic Editor

PLOS ONE

Journal Requirements:

When submitting your revision, we need you to address these additional requirements.

1. Please ensure that your manuscript meets PLOS ONE's style requirements, including those for file naming. The PLOS ONE style templates can be found at https://journals.plos.org/plosone/s/file?id=wjVg/PLOSOne_formatting_sample_main_body.pdf and https://journals.plos.org/plosone/s/file?id=ba62/PLOSOne_formatting_sample_title_authors_affiliations.pdf

2. Please include a complete copy of PLOS’ questionnaire on inclusivity in global research in your revised manuscript. Our policy for research in this area aims to improve transparency in the reporting of research performed outside of researchers’ own country or community. The policy applies to researchers who have travelled to a different country to conduct research, research with Indigenous populations or their lands, and research on cultural artefacts. The questionnaire can also be requested at the journal’s discretion for any other submissions, even if these conditions are not met. Please find more information on the policy and a link to download a blank copy of the questionnaire here: https://journals.plos.org/plosone/s/best-practices-in-research-reporting. Please upload a completed version of your questionnaire as Supporting Information when you resubmit your manuscript.

3. We note that you have indicated that there are restrictions to data sharing for this study. For studies involving human research participant data or other sensitive data, we encourage authors to share de-identified or anonymized data. However, when data cannot be publicly shared for ethical reasons, we allow authors to make their data sets available upon request. For information on unacceptable data access restrictions, please see http://journals.plos.org/plosone/s/data-availability#loc-unacceptable-data-access-restrictions.

Before we proceed with your manuscript, please address the following prompts:

a) If there are ethical or legal restrictions on sharing a de-identified data set, please explain them in detail (e.g., data contain potentially identifying or sensitive patient information, data are owned by a third-party organization, etc.) and who has imposed them (e.g., a Research Ethics Committee or Institutional Review Board, etc.). Please also provide contact information for a data access committee, ethics committee, or other institutional body to which data requests may be sent.

b) If there are no restrictions, please upload the minimal anonymized data set necessary to replicate your study findings to a stable, public repository and provide us with the relevant URLs, DOIs, or accession numbers. Please see http://www.bmj.com/content/340/bmj.c181.long for guidelines on how to de-identify and prepare clinical data for publication. For a list of recommended repositories, please see https://journals.plos.org/plosone/s/recommended-repositories. You also have the option of uploading the data as Supporting Information files, but we would recommend depositing data directly to a data repository if possible.

Please update your Data Availability statement in the submission form accordingly.

4. In the online submission form, you indicated that data are available in reasonable request to corresponding author.

All PLOS journals now require all data underlying the findings described in their manuscript to be freely available to other researchers, either 1. In a public repository, 2. Within the manuscript itself, or 3. Uploaded as supplementary information.

This policy applies to all data except where public deposition would breach compliance with the protocol approved by your research ethics board. If your data cannot be made publicly available for ethical or legal reasons (e.g., public availability would compromise patient privacy), please explain your reasons on resubmission and your exemption request will be escalated for approval.

5. Please include captions for your Supporting Information files at the end of your manuscript, and update any in-text citations to match accordingly. Please see our Supporting Information guidelines for more information: http://journals.plos.org/plosone/s/supporting-information.

6. Please update your submission to use the PLOS LaTeX template. The template and more information on our requirements for LaTeX submissions can be found at http://journals.plos.org/plosone/s/latex.

Additional Editor Comments :

Please take into account each of the referee suggestions and revise accordingly.

[Note: HTML markup is below. Please do not edit.]

Reviewers' comments:

Reviewer's Responses to Questions

**Comments to the Author**

1. Is the manuscript technically sound, and do the data support the conclusions?

Reviewer #1: Partly

Reviewer #2: Yes

Reviewer #3: Partly

**********

2. Has the statistical analysis been performed appropriately and rigorously?

Reviewer #1: Yes

Reviewer #2: No

Reviewer #3: No

**********

3. Have the authors made all data underlying the findings in their manuscript fully available?

The PLOS Data policy

Reviewer #1: No

Reviewer #2: No

Reviewer #3: No

**********

4. Is the manuscript presented in an intelligible fashion and written in standard English?

Reviewer #1: Yes

Reviewer #2: Yes

Reviewer #3: Yes

**********

Reviewer #1: Dear authors,

Thank you for your important work addressing methamphetamine (MA) use among Lahu youth—a critical public health issue with significant societal implications. Your manuscript is a sound technical piece. However, you can still improve it more to convince the reader that your methods were rigor. Few more information about the methods and materials will do that for you :-). Several statistical findings on questionnaire validation data, pilot tests, sensitivity tests and VIF will enhance the availability of your data. Overall, your paper is well structured. But, further synthesis (e.g., merging repetitive points) and explicit limitations would bolster its impact. Therefore, please find the detailed review sheets for your further perusal and wishing you all the very best! I appreciate your dedication to this vital topic and thank you for the opportunity given to read and review your paper.

Reviewer #2: PONE-D-25-27632

Factors influencing methamphetamine use among Lahu hill tribe youth Chiang Rai, Thailand: A case‒control study

• In the following page and line numbers will be given as: page.#

The printed page in the file will be used for the body of the text for page number.

• Edit suggestions (some stylistic, some substantive) will be given as: a => b (change a to b). [my explanations as to why appear in square brackets]

Review

This research investigates factors influencing methamphetamine use among Lahu hill tribe youth in norther Thailand using a case-control study with 136 cases (using) and 272 controls (not using), mostly male (79.4%) across four districts of Chiang Rai Province. It is an important topic.

Various predictors are tested for significance between cases and controls and a regression model employed thereafter. It is not clear why the variables were specifically chosen in the logistic regression given there are many that are significant in the univariate comparison between cases and controls. (One can’t include everything, but articulate why particular ones are chosen. Perhaps one his highly correlated with others, so it stands as a proxy for several – for example the similar significance of use of Twitter (X) and Instagram by controls (about 1 in 5) but much less by cases (p <0.001).

Several predictors were identified as significant in the model, but the confidence intervals are exceedingly wide around the estimate (for example an odds ratio of 6.40 for No schooling had a 95% CI of (1.12, 34.17) and Heroin Use had an odds ratio of 26.85 and 95% CI of (4.17, 172.95).

From a distance, the wide CI’s could be due to two or more predictors in the model having high correlation resulting in multicollinearity which will inflate the confidence intervals. Where high correlation between two predictors is the case, one predictor may more or less stand as a proxy also for the other giving justification for eliminating one from the model.

Given this possibility the authors should run a correlation matrix for the possible predictors in the model and check whether a correlation of 0.7 or more exists between two or more predictors. If logically related one can drop one. This should tighten the confidence intervals. One could run it with one and then the other to see how each impacts the other variable results as well – without too much fishing. Also one variable might be more important for the context thematically which might inform which to keep even if it is slightly less predictive. It may be that two logically unrelated predictors have high correlation by chance, which leaves one a choice on whether or not to eliminate one. If both being in inflates the Cis, then one should likely eliminate one of the predictors, noting their correlation in any case.

Whatever model is settled on, do mention the predictor that was dropped and its correlation level with the one kept if a reader might ask why a seemingly important variable found significant in a simple comparison between cases and controls was not in the multivariable model.

• Insert a table with AOR, 95%CI, p-values only for the multivariable logistic regression results where all of the included variables and results are together rather than results in the long list of univariate results separated far from each other in the long Table 4 (Factors, pages 16-24). That will make it much easier to read, edit, and comment on the logistic regression predictor selections and results.

• Also move Variables [Lines 5.25-28] on variables in the multivariate logistic regression to just before the new table after Table 4 with only with regression results. Note that there are many independent variables (possible predictors) that are significant but only a subset are included in the model. State how were they selected for the model (for example no social media variable was included although some are very significant univariately). See note above also concerning collinearity.

• When “controlling for age” in the multivariable model, is age the actual age of the person in years or the categorical Age variable listed as 15-19 and 20-24 years? (If actual age was there a coefficient and significance for the change in the log-odds of the outcome for each year above age 15; if categorical, was there an AOR for the variable?)

• Concerning Data Availability “No – some restrictions will apply” – explain the restrictions (what and why).

• On the Table called Factors covering pages 17-24 the percentages in parentheses should sum to be 100% vertically, not horizontally. That most are about 1/3 to 2/3 horizontally is due to the fact that there are only half as many cases as controls. Given different denominators 33.3 and 66.7 horizontally actually means 50% of cases and 50% of controls, which would show as 50.0 and 50.0 if summed vertically. The percentages for all Cases and Controls in the table have to be summed vertically.

• Page 27: A section labeled “Limitations” needs to be added after the Discussion. How generalizable are the results among all Lahu youth? For example the cases all have been in a treatment program but some MA users are not in a treatment program. Could there be differences between the MA users in treatment and those not reaching a treatment program? (How does one get into a treatment program?) Would one expect similar results in other hill tribe groups? Do the results compare with studies of other hill tribe groups (if there are such studies)? What are some limitations and advantages of Case-Control design in general?

Abstract

Background:

• 1.23 [page1.line23]: social insecurity => social security

[ impact on “health” before is a positive term, so impact on security keeps the second consistent]

• 1.24-25: [are the Lahu the second largest hill tribe in Thailand/northern Thailand? Or are Lahu youth identified as the second largest marginalized subpopulation within the Lahu tribe? If so which is the largest hill tribe marginalized population? Clarify.]

• [Perhaps:] 1.25: population => subpopulation

Results:

• [see comment above about large confidence intervals as indicating possible multicollinearity from correlated predictors in the logistic regression model – rerun model after if finding this to be the case and eliminating a predictor]

• 2.2: “After controlling for age”

[is actual age included as a continuous variable, is the two-level categorical variable Age (15-19, 20-24) used? is there a coefficient and significance related to Age in the regression model? The univariate comparison for age as a categorical variable was not significant.]

Conclusions: [Good]

Keywords: [Good]

Introduction:

3.9-10: part of the route of MA import, which is imported from neighboring countries,

=> part of the route of MA import from neighboring countries, then

3.11: and being => or being

3.13: uneconomic => subsistence

3.16: live under => live below [just sounds a little better]

3.18: including health care services

=> including health care services, because [tell why not granted a Thai ID card]

3.21: to MA use, => for MA use,

3.22-23: This study aimed to => The present study aims to

Methods:

3.32: case-control => case-control study

4.1: the odds ratio from the previous study => the ratio from a previous study

[ratio of controls to cases not odds-ratio? … it sounds like a simple ratio.]

4.2: a total of 136 cases and 272 controls

=> a total of 136 cases and 272 controls (a control/case ratio of 2.0)

4.8: been recorded in the MA treatment program as a client.

[Could Lahu MA-using youth recorded in a MA treatment program differ from Lahu MA-using youth who never make it to a treatment program? Mention this possibility or address this in the Discussion or under Limitations.]

4.25: gender with three options for their response: male, female, or other

[no respondent indicated ‘other’ for Gender in Table 1 on page 7. In the text ‘sex’ is used as well as gender. I would use only use ‘gender’ to be consistent with the questionnaire unless meaning biological sex exactly and not social/psychological identification. Also in Thai language is there a difference between “sex” and “gender” in the word used in the questionnaire? Among Lahu people generally (in contrast to academic researchers) “gender” may simply be understood as biological sex.

4.26: social media sue => social media use

5.25-28 [Section on Variables … comment above on moving these lines to just before a table with multivariate logistic regression results collected together in one table only with selected predictors.]

5.29-6.17 Recruitment procedure [good section on Recruitment procedure]

6.18-27 Statistical analysis [Good section – other comments on variable selection elsewhere.]

Note that Pseudo R-squared of logistic regression does not indicate proportion of variation of the dependent variable explained by the independent predictors as is the case of R-squared for linear regression. Pseudo R-squared in logistic regression context indicates the relative improvement in model fit compared to a baseline model (often a null model without predictors but only an intercept – that is the overall proportion in the data of the dependent variable being = 1 (which is trivial in a Case-Control set up where the cases = 1 and comprise one-third of the data by design as 2 controls = 0 have been included for every case such that controls =0 are two-thirds of the data).

For the Hosmer-Lemeshow test a high p-value is good (better fit) and a low p-value indicates a bad fit. This test, which uses a chi-squared approach, has been shown to be sensitive to the number of data partitions it uses (generally 10 in most software packages by default). A different number of partitions gives a different significance level for the same data. For this reason some researchers advise against using it although it is used in SPSS. It does provide some indication of goodness of fit of the model.

• 6.28-7.2: Ethical approval and consent to participate [Good section]

• 7.3: Results

• 7.13-9.1: Table 1 Comparisons of general characteristics between case and control [Table is OK]

• 9.7: [New paragraph - indent]

• 9.7: Regarding Substance use behaviors => Regarding Substance use behaviors in Table 2 below,

• 9.15-16: Facebook (78.2%) and Line (69.9%) were the most commonly used social media platforms.

=> Facebook (78.2%) and Line (69.9%) were the most commonly used social media platforms, but use levels were not significantly different between cases and controls.

• 9.16-17: sharing personal challenges with family members (p value<0.001)

[move to bottom of paragraph]

• 9.16-17: sharing personal challenges with family members (p value<0.001)

=> sharing personal challenges with family members was stark in contrast with only 36.0% of cases doing so compared to 62.5% of controls (p value<0.001) – cases were much more isolated from family in communication.

• 9.17-18: using Instagram (p value<0.001), and using Twitter (p-value<0.001) (Table 2).

=> While about 1 in 5 controls used Twitter (X) and Instagram, cases used both much less (2.9% and 5.1%) both with p-value<0.001 (Table 2).

• For long tables, see if there is a box for repeating the table column labels at the top of each page if the table covers multiple pages.

• 12.2: Two-thirds (71.6%) of the participants had their parents living together.

[Move this to Page 7 for discussion of Table 1 which has this variable.]

Two-thirds (71.6%) of the participants had their parents living together.

=> The parents of cases lived together 18% less than controls (p<.001).

• 12.2: Half of => In Table 3 below, half of

• 12.16 (Table 3). => Cases had much higher relative access to cannabis, opium, and heroin than Controls (all p<.001).

• Page 13: [note “Having been persuaded by friend to use MA” has 9.6% of Controls saying “Yes” which should be 0% by the inclusion criteria. Perhaps the 9.6% of cases indicates that a friend attempted to persuade them but did not succeed.]

• 15.11: [How were the predictors selected for inclusion in the multiple logistic regression model given so many significant univariate variables were not included?]

• 15.11: [Is age the actual age in years or the two-level categorical variable from Table 1? Did age have a coefficient or odds ratio and a significance level?]

• 15.11-15.22: [See comment above about inflated confidence intervals (CI’s) and the need to check for collinearity and rerunning model with collinearity reduced.]

• Page 16: [Table 4 should start on this page beneath the heading. Almost blank page.]

• [For Table 4 AOR, 95%CI, p-value for the multivariate logistic regression can be gathered into a separate table for the model which is much more easy to read.]

• Pages 17-23: [The variable %’s in parentheses are now summed horizontally for each Factor in Table 4. These must be summed vertically to sum to 100%. Horizontally the cases are mostly about 1/3 and controls about 2/3 as that is the proportion of the design.]

• Page 24: The significance note should appear on Page 23 under the end of the table.

Page 25: Discussion

• 25.2: use, including => use including [delete comma]

• 25.3: high knowledge of MA and control,

=> high knowledge of MA prevention and control,

• 25.3: and being => being

• 25.13: due to their health literacy => due to their health literacy being low

• 25.15: compulsory => necessary

• 25.16: Those => Seemingly paradoxically, those

• 25.24: prevention and control.

=> prevention and control. MA users not attending such clinics may also have a much lower level of knowledge as well.

• 25.27-28: smoking hill tribes, people living => hill tribes, people smoking living

• 25.28: did not. => did not smoke.

• 26.16: school. We also found that having no family member to share a personal challenge was true for 2/3 of the cases compared to 1/3 of the controls.

• Lines 26.23-31 [move to section titled “Limitations” perhaps after 27.8 or before Conclusion at 26.32]

• 27.1: organizational layers => structural layers

• [Insert blank line after 27.8]

Page 27: A section labeled “Limitations” needs to be added after the Discussion. Move Lines 26.23-31 under Limitations.

• 27.12: my => our

• 27.14: village head => village heads

• 27:14: participant => participants

• 27.18: P Phootawan => Phootawan

References [good]

• 30.2: thai => Thai

• 30.10: Lahu ethnical, Thailand => [Lahu ethnicity, Thailand]

[square brackets for title as original is in Thai]

%%%%%%%%%%%%%%%%%%%%%%%%%%%%%%%%%%%%%%%%%%%

Thank you for the opportunity to review this important research.

Reviewer #3: I want to commend the authors for their work on this relevant topic. The research tackles a critical public health issue affecting underprivileged populations and demonstrates interesting innovations, such as the creative triple-check control verification method and rigorous objective biomarker validation—both of which advance the epidemiological science among marginalized communities. Please find below my suggestions and concerns, organized point-by-point.

Introduction:

• I'm curious about the national identification card system in Thailand, as this emerges as such an important factor in your findings. It would be helpful to understand the circumstances under which Lahu community members might not receive national identification cards and the implications this has for accessing services.

• Your introduction does an excellent job establishing the MA use challenges among Lahu youth. I wonder if you might consider sharing additional insights about other socio-cultural characteristics of this population? This could help readers like myself better understand the broader environment that contributes to these patterns.

Methods:

• The sample size calculation references a "previous study" (Thailand narcotics control annual report 2023). While the calculation is provided, it would enhance clarity to briefly explain how this reference study relates to the current methodological approach and justify the assumptions used.

• For improved readability, consider reorganizing the methods section by placing “recruitment procedure" immediately after "study sample," potentially subdividing this into "inclusion criteria" and "recruitment procedures." This would maintain the logical flow of understanding sample construction.

• If possible, I believe a participant flow diagram would benefit overall comprehension for the case-control by showing the selection process clearly.

• Regarding case selection from MA clinics: could the authors clarify whether this refers to a single clinic or multiple facilities? Additional context about the clinic setting(s) would help readers understand the representativeness of the case population.

• Your control selection process sounds very thorough. I am still unsure whether controls were drawn from the entire eligible Lahu community aged 15-24, or if there were additional selection considerations. You might also consider moving that first results paragraph (with the final sample sizes) into methods—it would really strengthen the transparency of your approach.

• While sex-matching is well-justified given known gender differences in MA use, could you elaborate on the decision not to match on other factors like age range? The rationale for matching choices is crucial in case-control design and would contribute to the methods description.

• The authors write that “There were several independent variables or predictors in the study, e.g., age, sex, marital status, education, occupation, family income, knowledge of MA prevention and control, and attitudes toward MA prevention and control.” I would suggest that they either clearly define the variables rather than putting out examples of what may appear in the study. For instance, STROBE reporting guidelines (doi:10.1371/journal.pmed.0040296) suggest we “clearly define all outcomes, exposures, predictors, potential confounders, and effect modifiers”, and the authors do provide some comprehensive description for variables in the “research instruments” sections. However, it’s preferable to have an objective perspective on variables used in the study, with clear definitions if not previously done. I suggest the authors revise their instruments and variables section to avoid redundant content and explicitly – and intentionally – present the selected variables, their treatment and application (e.g. outcome, covariate, exposure).

• I'm interested in your 15,000-baht household income threshold. Could you help us understand how this relates to Thai socioeconomic standards? I noticed you mention Thailand's national poverty line of $1,400/year per family in the introduction—connecting these would perhaps help.

• I think there might be a small calculation issue in Table 1's household income row (the percentages appear to sum to 109%)—just wanted to flag this for your review.

• Please clarify whether missing data analysis or sensitivity analyses were conducted. If not performed, this should be explicitly stated. If conducted, the patterns and handling methods would be valuable to report.

Results:

• Consider streamlining the presentation by reducing repetitive "p value" references throughout the text.

• The final (adjusted) multivariate model controls only for age despite multiple significant univariate associations (education, occupation, income, family status). Could you discuss the rationale for this approach and potential implications for residual confounding?

• Could you clarify whether you included age as a continuous variable or in categorical groups in your multivariate analysis? Just a small detail that would be helpful.

• Given the cross-sectional measurement of exposures and outcomes, could you address the temporal sequence challenges, particularly for variables like "knowledge of MA prevention and control"? This pattern suggests the knowledge was acquired through treatment programs after MA use initiation, rather than representing a causal pathway to use. For clarity, I would recommend the authors explicitly state that their counterintuitive findings regarding increased use associated with MA knowledge may be caused by reverse causation bias: knowledge came after MA use, not before.

• Some effect sizes appear unusually large with very wide confidence intervals (e.g., heroin use: AOR 30.14, CI: 4.29-211.95; friend persuasion: AOR 13.79, CI: 3.80-50.05). These suggest either very small cell sizes or potential data quality issues. Could you provide the actual numbers in these cells and discuss the reliability of these estimates?

Before concluding, I would also recommend the authors consider discussing study limitations more comprehensively, including potential selection biases, temporal sequence issues, and generalizability beyond this specific population.

**********

what does this mean? ). If published, this will include your full peer review and any attached files.

If you choose “no”, your identity will remain anonymous but your review may still be made public.

**Do you want your identity to be public for this peer review?** For information about this choice, including consent withdrawal, please see our Privacy Policy

Reviewer #1: No

Reviewer #2: **Yes:** Kirk C Allison

Reviewer #3: **Yes:** Cézar D. Luquine Jr.

**********

[NOTE: If reviewer comments were submitted as an attachment file, they will be attached to this email and accessible via the submission site. Please log into your account, locate the manuscript record, and check for the action link "View Attachments". If this link does not appear, there are no attachment files.]

While revising your submission, please upload your figure files to the Preflight Analysis and Conversion Engine (PACE) digital diagnostic tool, https://pacev2.apexcovantage.com/ . PACE helps ensure that figures meet PLOS requirements. To use PACE, you must first register as a user. Registration is free. Then, login and navigate to the UPLOAD tab, where you will find detailed instructions on how to use the tool. If you encounter any issues or have any questions when using PACE, please email PLOS at figures@plos.org . Please note that Supporting Information files do not need this step.

Attachment Submitted filename: ReviewReport_2025_PONE-D-25-27632.pdf

Attachment Submitted filename: PONE-D-25-27632 review.docx

# Author comment: Author response to Decision Letter 1

**DOI:** 10.1371/journal.pone.0344775.r002

21 Aug 2025

Response to editor and reviewers comments

PONE-D-25-27632

Factors influencing methamphetamine use among Lahu hill tribe youth Chiang Rai, Thailand: A case‒control study

PLOS ONE

Dear Dr. Apidechkul,

Thank you for submitting your manuscript to PLOS ONE. After careful consideration, we feel that it has merit but does not fully meet PLOS ONE’s publication criteria as it currently stands. Therefore, we invite you to submit a revised version of the manuscript that addresses the points raised during the review process.

==============================

Please take into account each of the referee suggestions and revise accordingly.

Looking forward to your revised version!

==============================

Please submit your revised manuscript by Aug 11 2025 11:59PM. If you will need more time than this to complete your revisions, please reply to this message or contact the journal office at plosone@plos.org. When you're ready to submit your revision, log on to https://www.editorialmanager.com/pone/ and select the 'Submissions Needing Revision' folder to locate your manuscript file.

Please include the following items when submitting your revised manuscript:

• A rebuttal letter that responds to each point raised by the academic editor and reviewer(s). You should upload this letter as a separate file labeled 'Response to Reviewers'.

• A marked-up copy of your manuscript that highlights changes made to the original version. You should upload this as a separate file labeled 'Revised Manuscript with Track Changes'.

• An unmarked version of your revised paper without tracked changes. You should upload this as a separate file labeled 'Manuscript'.

If you would like to make changes to your financial disclosure, please include your updated statement in your cover letter. Guidelines for resubmitting your figure files are available below the reviewer comments at the end of this letter.

If applicable, we recommend that you deposit your laboratory protocols in protocols.io to enhance the reproducibility of your results. Protocols.io assigns your protocol its own identifier (DOI) so that it can be cited independently in the future. For instructions see: https://journals.plos.org/plosone/s/submission-guidelines#loc-laboratory-protocols. Additionally, PLOS ONE offers an option for publishing peer-reviewed Lab Protocol articles, which describe protocols hosted on protocols.io. Read more information on sharing protocols at https://plos.org/protocols?utm_medium=editorial-email&utm_source=authorletters&utm_campaign=protocols.

We look forward to receiving your revised manuscript.

Kind regards,

Rining Wei, PhD

Academic Editor

PLOS ONE

Journal Requirements:

When submitting your revision, we need you to address these additional requirements.

1. Please ensure that your manuscript meets PLOS ONE's style requirements, including those for file naming. The PLOS ONE style templates can be found at https://journals.plos.org/plosone/s/file?id=wjVg/PLOSOne_formatting_sample_main_body.pdf and https://journals.plos.org/plosone/s/file?id=ba62/PLOSOne_formatting_sample_title_authors_affiliations.pdf

: Thank you, we have double checked before submission.

2. Please include a complete copy of PLOS’ questionnaire on inclusivity in global research in your revised manuscript. Our policy for research in this area aims to improve transparency in the reporting of research performed outside of researchers’ own country or community. The policy applies to researchers who have travelled to a different country to conduct research, research with Indigenous populations or their lands, and research on cultural artefacts. The questionnaire can also be requested at the journal’s discretion for any other submissions, even if these conditions are not met. Please find more information on the policy and a link to download a blank copy of the questionnaire here: https://journals.plos.org/plosone/s/best-practices-in-research-reporting. Please upload a completed version of your questionnaire as Supporting Information when you resubmit your manuscript.

: Thank you, we have submitted the STROBE into the system.

3. We note that you have indicated that there are restrictions to data sharing for this study. For studies involving human research participant data or other sensitive data, we encourage authors to share de-identified or anonymized data. However, when data cannot be publicly shared for ethical reasons, we allow authors to make their data sets available upon request. For information on unacceptable data access restrictions, please see http://journals.plos.org/plosone/s/data-availability#loc-unacceptable-data-access-restrictions.

Before we proceed with your manuscript, please address the following prompts:

a) If there are ethical or legal restrictions on sharing a de-identified data set, please explain them in detail (e.g., data contain potentially identifying or sensitive patient information, data are owned by a third-party organization, etc.) and who has imposed them (e.g., a Research Ethics Committee or Institutional Review Board, etc.). Please also provide contact information for a data access committee, ethics committee, or other institutional body to which data requests may be sent.

b) If there are no restrictions, please upload the minimal anonymized data set necessary to replicate your study findings to a stable, public repository and provide us with the relevant URLs, DOIs, or accession numbers. Please see http://www.bmj.com/content/340/bmj.c181.long for guidelines on how to de-identify and prepare clinical data for publication. For a list of recommended repositories, please see https://journals.plos.org/plosone/s/recommended-repositories. You also have the option of uploading the data as Supporting Information files, but we would recommend depositing data directly to a data repository if possible.

Please update your Data Availability statement in the submission form accordingly.

Since the data includes several sensitive issues that might be against ethical issue, we could provide the data. However, data could be requested from the corresponding author in some parts, such as general information.

4. In the online submission form, you indicated that data are available in a reasonable request to the corresponding author.

Yes, with reasonable requests to the corresponding authors, general information about participants could be accepted.

All PLOS journals now require all data underlying the findings described in their manuscript to be freely available to other researchers, either 1. In a public repository, 2. Within the manuscript itself, or 3. Uploaded as supplementary information.

This policy applies to all data except where public deposition would breach compliance with the protocol approved by your research ethics board. If your data cannot be made publicly available for ethical or legal reasons (e.g., public availability would compromise patient privacy), please explain your reasons on resubmission and your exemption request will be escalated for approval.

5. Please include captions for your Supporting Information files at the end of your manuscript, and update any in-text citations to match accordingly. Please see our Supporting Information guidelines for more information: http://journals.plos.org/plosone/s/supporting-information.

:Thank you,

6. Please update your submission to use the PLOS LaTeX template. The template and more information on our requirements for LaTeX submissions can be found at http://journals.plos.org/plosone/s/latex.

: Thank you

Additional Editor Comments :

Please take into account each of the referee suggestions and revise accordingly.

Comments to the Author

Reviewer #1: Dear authors,

Thank you for your important work addressing methamphetamine (MA) use among Lahu youth—a critical public health issue with significant societal implications. Your manuscript is a sound technical piece. However, you can still improve it more to convince the reader that your methods were rigor. Few more information about the methods and materials will do that for you :-). Several statistical findings on questionnaire validation data, pilot tests, sensitivity tests and VIF will enhance the availability of your data. Overall, your paper is well structured. But, further synthesis (e.g., merging repetitive points) and explicit limitations would bolster its impact. Therefore, please find the detailed review sheets for your further perusal and wishing you all the very best! I appreciate your dedication to this vital topic and thank you for the opportunity given to read and review your paper.

Review Report: Factors influencing methamphetamine use among Lahu hill tribe youth Chiang Rai, Thailand: A case‒control study

Dear authors,

It is remarkable that you have clearly pointed out MA as a public health issue, and the Lahu youth population is vulnerable to that. Yet, I would like to clear some concerns and provide several comments to further enhance the understandability of the manuscript.

Abstract:

The abstract is well structured. However, there are some minor concerns from my side, which I might need a little clarification or a slight modification to the written abstract. Please address them accordingly:

1.Under the methods: “... The participants were recruited from...”. Could you please clarify the sampling method here?

: We used a random method to recruit the sample. Please see page 1, line 31.

2.The authors stated, “A validated questionnaire was used to collect data, and multiple logistic regression was used to detect association factors at α = 0.05.” The use of the term “detect association factors at α = 0.05” is understandable. However, to make it technically sound, would you consider rewording the statement to provide an idea like “...with statistical significance set at α = 0.05...”? Such a clear expression would avoid any ambiguity.

: Thank you for the comment. We agree with you and have revised accordingly. Please see page 1, line 34.

3. When I was referring to the data collection instrument of your study, I noticed that the expected outcomes of your study are not always binary. If the outcome measures are binary, it is technically accurate to use the “multiple logistic regression”; otherwise, it is recommended to use linear or ordinal regression for the data analysis. Please recheck your data analysis plan and supplement with related information in your methods section.

: Thank you for the comment. Our study outcome was whether to use AM or not, which was set at the beginning. Then we used the logistic regression instead of linear regression.

Introduction:

Informative approach in introducing the scope of your research area and the aims. You have mentioned the neurobiological effects of using MA and also the societal consequences. Special emphasis on the socio-economic disparities in the context of Thailand's geographical risks is an important fact.

4. However, the lines 11 to 19 do not create a bridge between the MA use with the socio- economic disparities that you have emphasised there, including the ‘education’. Could you please elaborate on the association between the MA use and the mentioned socio- economic factors in the lines? Or could you briefly mention why Lahu youth are at higher risk by referring to the socioeconomic factors and geographic isolation?

Thank you for the great comment. We have extended details in page 3, lines 18-20.

5. It is appreciated that you have cited sources from both the Thai-originated sources and the international sources. When considering the logical flow of your introduction, it can be guessed that you have focused first on bringing the readers’ attention to the global scenario, then Thailand, then Chiang Rai in Thailand, and the Lahu youth. However, it would be better if you could also add a paragraph to emphasise the gaps in the literature, which should be a critical element of an introduction to a manuscript. Because, in your introduction, it was not explicitly stated why previous research was not sufficient, or why the previous research was not available in your area of interest as a controlled case study.

: Thank you for your excellent comment. We have added information in page 3, lines 24-28.

6.Lines 22 to 24: You mentioned the study objective. However, there was no justification in the introduction on why the case-control method was selected. Could you briefly mention why you have selected the case-control study design? It is highly recommended to clarify/state the hypotheses rather than hinting it in a descriptive way through the research aim. Please consider modifying the way you report the aims by giving more focus into your study’s hypotheses.

: Thank you for the comment. We totally agree with your comment that to guide the direction to readers, we need a hypothesis. Please see page 4, lines 3-5.

7.It is suggested to use your statements in lines 20-22 in the discussion section rather than in the introduction section.

: Thank you for the comment. With the context of the existing sentence focused on general discussion, we have modified the paragraph to be the basis of the idea to raise the study objective in this paragraph. Thank you very much. Please see lines page 3, 24-29,

8.Because your overall conclusion is to emphasise the need for “effective interventions and changes in policy” the introduction must expand a bit its concluding remarks by supplementing information about the policy relevance of this study.

: Thank you for such a great idea. We have extended the information on page 3, lines 29-30.

Materials and Methods:

The methods section is satisfactorily planned. However, the following comments/ questions will help you to increase the readability and broaden the understanding of your study for readers with less understanding of statistical methods. Please consider modifying your writing based on the following:

9.Line 27: The same comment (comment 3 of the introduction) applies here. You may address it under study design.

: Thank you. Please see the information added on page 3, lines 28-30.

10.Lines 29 to 30: Please consider using full sentences. Could you rewrite for better readability?

: Thank you for the comment. It is revised and completed. Please see page 4, lines 3-5

11.The sample size was calculated appropriately. However, to ensure expanded readability, it is better if you could add notes for the mentioned variables/constants/functions/symbols in the equation. This would help readers who may not have a wider understanding of equation-related statistical symbolic expressions.

: Thank you, we have added information in all notes used.

12. Line 10 onwards: To enhance the objective verification, could you mention whether there were complementary self-reports compared to the urine test results? If the results were not complementary, what was the next step?

: Thank you for the great comment. We have revised the sentences in lines 17-21 on page 4 to ensure that the information has been cleared for readers. Individuals who responded to any of the three methods with “Yes” were assumed to be a case (MA user).

13.The triple triple-check method is obviously a strong method to ensure the selection validity of your sample. Yet, despite the triple-check, there might still be social desirability bias among the controls as the MA use can be under-reported because of its stigma. Could you please try considering this as a fact when addressing the previous comment, too?

Thank you. We do know that with the conditions of illegal MA use in Thailand and also social desirability bias, which could be either a higher or lower possibility to say “yes” or “no” for some people, but it is the best way that we could do at the moment. We have put our limitations of the study on page 24, lines 17-32. Please also see my response to question No.14.

14.Also, the controls from the same village might share unmeasured confounders such as access to the MA. How have you addressed this as a fact?

: We selected cases and controls randomly after being classified by the triple method, and before the interview, they did not know about the process of identifying/assessing, including the questions asked, which are common to a case-control study. We

Attachment Submitted filename: Respone to reviewer comments .pdf

# Peer review: Decision Letter 1

**DOI:** 10.1371/journal.pone.0344775.r003

**Authors:** Steve Zimmerman

28 Oct 2025

Dear Dr. Apidechkul,

Thank you for submitting your manuscript to PLOS ONE. After careful consideration, we feel that it has merit but does not fully meet PLOS ONE’s publication criteria as it currently stands. Therefore, we invite you to submit a revised version of the manuscript that addresses the points raised during the review process.

However, each of the three reviewers still has some requests for further improvements to the manuscript - please see their comments below.

I also have a concern with the categorisation of continuous variables, specifically the knowledge and attitude scores, although this applies to age as well.

You have dichotomised or trichotomised these variables for the logistic regression analysis. However, you have not provided a reason why knowledge and attitude scores should be split into three categories, or why the cutoffs should differ across these two measures. None of the sources you cite (refs 29-31) treat continuous variables in this manner (with the exception of a single analysis in ref 29). Unless there is good reason to do so (e.g., bimodal data), continuous variables should not be changed into categorical variables. Knowledge, attitude, and age can all be entered as continuous predictor variables in logistic regression. Please could you re-run your analyses with continuous predictors where relevant/appropriate?

Could you also please carefully revise the manuscript to address all comments raised by the reviewers?

Please submit your revised manuscript by Dec 12 2025 11:59PM. If you will need more time than this to complete your revisions, please reply to this message or contact the journal office at plosone@plos.org . When you're ready to submit your revision, log on to https://www.editorialmanager.com/pone/ and select the 'Submissions Needing Revision' folder to locate your manuscript file.

- A rebuttal letter that responds to each point raised by the academic editor and reviewer(s). You should upload this letter as a separate file labeled 'Response to Reviewers'.
- A marked-up copy of your manuscript that highlights changes made to the original version. You should upload this as a separate file labeled 'Revised Manuscript with Track Changes'.
- An unmarked version of your revised paper without tracked changes. You should upload this as a separate file labeled 'Manuscript'.

If you would like to make changes to your financial disclosure, please include your updated statement in your cover letter. Guidelines for resubmitting your figure files are available below the reviewer comments at the end of this letter.

If applicable, we recommend that you deposit your laboratory protocols in protocols.io to enhance the reproducibility of your results. Protocols.io assigns your protocol its own identifier (DOI) so that it can be cited independently in the future. For instructions see: https://journals.plos.org/plosone/s/submission-guidelines#loc-laboratory-protocols . Additionally, PLOS ONE offers an option for publishing peer-reviewed Lab Protocol articles, which describe protocols hosted on protocols.io. Read more information on sharing protocols at https://plos.org/protocols?utm_medium=editorial-email&utm_source=authorletters&utm_campaign=protocols .

We look forward to receiving your revised manuscript.

Kind regards,

Steve Zimmerman, PhD

Senior Editor, PLOS One

Journal Requirements:

If the reviewer comments include a recommendation to cite specific previously published works, please review and evaluate these publications to determine whether they are relevant and should be cited. There is no requirement to cite these works unless the editor has indicated otherwise.

[Note: HTML markup is below. Please do not edit.]

Reviewers' comments:

Reviewer's Responses to Questions

**Comments to the Author**

Reviewer #1: All comments have been addressed

Reviewer #2: All comments have been addressed

Reviewer #3: (No Response)

**********

2. Is the manuscript technically sound, and do the data support the conclusions?

Reviewer #1: Yes

Reviewer #2: Yes

Reviewer #3: Partly

**********

3. Has the statistical analysis been performed appropriately and rigorously?

Reviewer #1: (No Response)

Reviewer #2: Yes

Reviewer #3: I Don't Know

**********

4. Have the authors made all data underlying the findings in their manuscript fully available?

The PLOS Data policy

Reviewer #1: (No Response)

Reviewer #2: Yes

Reviewer #3: Yes

**********

5. Is the manuscript presented in an intelligible fashion and written in standard English?

Reviewer #1: Yes

Reviewer #2: Yes

Reviewer #3: Yes

**********

Reviewer #1: Dear Authors,

Thank you for responding to the review comments and being patient enough with providing further clarifications. There is one more suggestion from my side. Could you please consider replacing the statement "Missing data were checked before further analysis" to indicate that missing data were not reported?

Wishing you all the very best!

Reviewer #2: PONE-D-25-27632 Resubmission Review

Thank you to the authors for the responsive revisions.

The major issues have been addressed in the revision including correction of tables column summing and introduction of a separate table for the multivariate analysis (Table 5). The issue of the wide confidence intervals for certain variables was investigated concerning possible multicollinearity (with one variable dropped) using variable correlation and VIF (variance inflation factor) investigation. Small cell size was also identified as a possible culprit.

Below are a list of minor edits from reading the revised manuscript. I do not need to see these edits. Here again page and line # is indicated as page.line#. a=>b means change a to b. [Comments in brackets simply clarify the change.]

- Throughout : p value or p-value

[as the editor prefers … in the US it is mostly p-value]

- 4.28: :: => : [delete one colon]

- 7.19: drop => dropped

- 7.24: were => was [singular, agrees with ‘reliability’ as subject]

- 8.9: determine => examine

- 21.9: fold( => fold ( [insert space]

- Page 22 Table 5: [add * after Yes/Ever p-value concerning Heroin use]

0.001 => 0.001*

- 23.16: Either smoking or heroin use, was identified

=> Both smoking and heroin use were identified

- 23.22: particularly young adults => particularly among young adults

- 23.26: use of cod heroin and MA => use of codrug heroin and MA

or: => use of heroin and MA [not sure what ‘cod’ means]

- 24.11: MA access => MA access near borders

- 24.24: difficult to => unclear whether

- 25.9: laws => existing laws

[as in complement existing laws? Or perhaps meaning strengthen existing laws?]

Thanks again for working on this important area.

Reviewer #3: I thank the authors for their thorough effort in responding to our feedback. It shows that you have tried to answer to the variety of suggestions and comments reviewer put forward. While you've demonstrated understanding of the methodological concerns raised, a significant disconnect exists between your explanations to reviewers and what appears in the manuscript. Readers would greatly benefit from the clarifications you provided in your responses being incorporated into the manuscript text.

Your responses contained valuable explanations for: sample size calculation rationale based on the Thailand narcotics control report; the logic behind different income thresholds (15,000 baht for graduates vs. $1,400 poverty line); your data completion procedures that minimized missing values; the rationale for age-only adjustment in the final model; and the categorization of age variables. These explanations must be incorporated into the manuscript as they address fundamental questions readers may have about your methodology.

Additionally, while you explained age adjustment to reviewers, the final model's exclusion of other significant univariate factors (education, occupation, income, family status) despite your acknowledgment of “wide CIs due to small cells” suggests model instability rather than appropriate selection. Consider reporting exact cell counts for problematic variables and discussing precision limitations explicitly or use formal model selection criteria to justify your approach.

**********

what does this mean? ). If published, this will include your full peer review and any attached files.

If you choose “no”, your identity will remain anonymous but your review may still be made public.

**Do you want your identity to be public for this peer review?** For information about this choice, including consent withdrawal, please see our Privacy Policy

Reviewer #1: **Yes:** D.L.I.H.K. PEIRIS

Reviewer #2: **Yes:** Kirk C Allison

Reviewer #3: **Yes:** Cézar Luquine

**********

[NOTE: If reviewer comments were submitted as an attachment file, they will be attached to this email and accessible via the submission site. Please log into your account, locate the manuscript record, and check for the action link "View Attachments". If this link does not appear, there are no attachment files.]

To ensure your figures meet our technical requirements, please review our figure guidelines: https://journals.plos.org/plosone/s/figures

You may also use PLOS’s free figure tool, NAAS, to help you prepare publication quality figures: https://journals.plos.org/plosone/s/figures#loc-tools-for-figure-preparation.

NAAS will assess whether your figures meet our technical requirements by comparing each figure against our figure specifications.

# Author comment: Author response to Decision Letter 2

**DOI:** 10.1371/journal.pone.0344775.r004

12 Nov 2025

Response to reviewers comments

Dear Editor and reviewers,

Thank you very much for the opportunity to revise again with valuable comments and feedback from you. We have carefully revised based on all your comments and feedback. We re-analyzed the whole data set by treating age and knowledge in continuous forms, and the final model looks better than the previous one. Please see details on our feedback below, and very hope you are happy with this version.

Thank you,

TK

Thank you for submitting your manuscript to PLOS ONE. After careful consideration, we feel that it has merit but does not fully meet PLOS ONE’s publication criteria as it currently stands. Therefore, we invite you to submit a revised version of the manuscript that addresses the points raised during the review process.

The three original reviewers have assessed your revised manuscript and thank you for addressing the issues they raised during the first round of review.

However, each of the three reviewers still has some requests for further improvements to the manuscript - please see their comments below.

I also have a concern with the categorisation of continuous variables, specifically the knowledge and attitude scores, although this applies to age as well.

1. You have dichotomised or trichotomised these variables for the logistic regression analysis. However, you have not provided a reason why knowledge and attitude scores should be split into three categories, or why the cutoffs should differ across these two measures. None of the sources you cite (refs 29-31) treat continuous variables in this manner (with the exception of a single analysis in ref 29). Unless there is good reason to do so (e.g., bimodal data), continuous variables should not be changed into categorical variables. Knowledge, attitude, and age can all be entered as continuous predictor variables in logistic regression. Please could you re-run your analyses with continuous predictors where relevant/appropriate?

: Thank you very much for the comments. We have re-analyzed the whole process in a stepwise with pr.(0.2) for selection variables into the model, and treated age and knowledge in continuous forms. The final model, ten (10) variables were retained in the model for predicting, and seven 97) variables were found to be associated with MA use. We have revised all relevant information, including Table 5. Please see page 2, lines 3-13; page 22, lines 3-15, table 5, discussion and conclusion sessions.

We do hope that you are happy with this version.

Reviewers' comments:

Reviewer #1: Dear Authors,

2. Thank you for responding to the review comments and being patient enough with providing further clarifications. There is one more suggestion from my side. Could you please consider replacing the statement "Missing data were checked before further analysis" to indicate that missing data were not reported?

: Thank you for the comment. We have modified the statement to “Missing data were checked and managed properly before further analysis.” Please see page 7, lines 29-30.

Reviewer #2: PONE-D-25-27632 Resubmission Review

Thank you to the authors for the responsive revisions.

The major issues have been addressed in the revision including correction of tables column summing and introduction of a separate table for the multivariate analysis (Table 5). The issue of the wide confidence intervals for certain variables was investigated concerning possible multicollinearity (with one variable dropped) using variable correlation and VIF (variance inflation factor) investigation. Small cell size was also identified as a possible culprit.

Below are a list of minor edits from reading the revised manuscript. I do not need to see these edits. Here again page and line # is indicated as page.line#. a=>b means change a to b. [Comments in brackets simply clarify the change.]

3.- Throughout : p value or p-value

[as the editor prefers … in the US it is mostly p-value]

: Thank you for the notice. We totally agreed with you and have changed to p-value all points used in the paper.

4.- 4.28: :: => : [delete one colon]

: Thank you, it’s improved

5.- 7.19: drop => dropped

: Thank you, it’s improved

6.- 7.24: were => was [singular, agrees with ‘reliability’ as subject]

: Thank you, it’s improved

7.- 8.9: determine => examine

: Thank you, it’s improved

8. - 21.9: fold( => fold ( [insert space]

: Thank you, it’s improved

9.- Page 22 Table 5: [add * after Yes/Ever p-value concerning Heroin use]

0.001 => 0.001*

: Thank you, it’s improved

10. - 23.16: Either smoking or heroin use, was identified

=> Both smoking and heroin use were identified

: Thank you, it’s improved

11. - 23.22: particularly young adults => particularly among young adults

: Thank you, it’s improved

12. - 23.26: use of cod heroin and MA => use of codrug heroin and MA

or: => use of heroin and MA [not sure what ‘cod’ means]

: Thank you, it’s improved, it is “codrug..”

13. - 24.11: MA access => MA access near borders

: Thank you, it’s improved

14. - 24.24: difficult to => unclear whether

: Thank you, it’s improved

15.- 25.9: laws => existing laws

[as in complement existing laws? Or perhaps meaning strengthen existing laws?]

: Thank you, it’s improved

Reviewer #3: I thank the authors for their thorough effort in responding to our feedback. It shows that you have tried to answer to the variety of suggestions and comments reviewer put forward. While you've demonstrated understanding of the methodological concerns raised, a significant disconnect exists between your explanations to reviewers and what appears in the manuscript. Readers would greatly benefit from the clarifications you provided in your responses being incorporated into the manuscript text.

16. Your responses contained valuable explanations for: sample size calculation rationale based on the Thailand narcotics control report; the logic behind different income thresholds (15,000 baht for graduates vs. $1,400 poverty line); your data completion procedures that minimized missing values; the rationale for age-only adjustment in the final model; and the categorization of age variables. These explanations must be incorporated into the manuscript as they address fundamental questions readers may have about your methodology.

: Thank you for such great comments. We have added information on the main manuscript. Please see page 4, lines 10-11/ page 7, lines 33-34/ page 8, lines 1.

17. Additionally, while you explained age adjustment to reviewers, the final model's exclusion of other significant univariate factors (education, occupation, income, family status) despite your acknowledgment of “wide CIs due to small cells” suggests model instability rather than appropriate selection. Consider reporting exact cell counts for problematic variables and discussing precision limitations explicitly or use formal model selection criteria to justify your approach.

: Thanks for the great comment. We have revised the whole process of the analysis again using the stepwise method at selection propagability pr.(02) after putting all variables in the model and treating knowledge and age at continuing forms as recommended. It was found that 10 variables were found to be associated with MA use. Among the remained variables in the stepwise mode, three variables presented in the previous version were not added as the predictors: heroin use, having been persuaded by a friend, and smoking cigarettes.

While another six variables were added into the model as the predictors for the MA use: sex, cigarette accessibility, marital status, parent living status, occupation, and alcohol assumption (see Table 5). We have seriously considered together among the authors and agreed that this new model is much better than the previous one and is be used as our final model. At the end, seven (7) variables were associated with MA use. Then, we have modified the relevant context and added information in the discussion part. We thank you so much for these great suggestions. Please see page details in Table 5, and page 22, lines 3-15.

Thank you,

TK

Tawatchai Apidehckul

Attachment Submitted filename: MA_Reviewers commenst.pdf

# Peer review: Decision Letter 2

**DOI:** 10.1371/journal.pone.0344775.r005

**Authors:** Aditya Pawar

11 Dec 2025

Dear Dr. Apidechkul,

Thank you for submitting your manuscript to PLOS ONE. After careful consideration, we feel that it has merit but does not fully meet PLOS ONE’s publication criteria as it currently stands. Therefore, we invite you to submit a revised version of the manuscript that addresses the points raised during the review process.

Please submit your revised manuscript by Jan 25 2026 11:59PM. If you will need more time than this to complete your revisions, please reply to this message or contact the journal office at plosone@plos.org . When you're ready to submit your revision, log on to https://www.editorialmanager.com/pone/ and select the 'Submissions Needing Revision' folder to locate your manuscript file.

- A rebuttal letter that responds to each point raised by the academic editor and reviewer(s). You should upload this letter as a separate file labeled 'Response to Reviewers'.
- A marked-up copy of your manuscript that highlights changes made to the original version. You should upload this as a separate file labeled 'Revised Manuscript with Track Changes'.
- An unmarked version of your revised paper without tracked changes. You should upload this as a separate file labeled 'Manuscript'.

If applicable, we recommend that you deposit your laboratory protocols in protocols.io to enhance the reproducibility of your results. Protocols.io assigns your protocol its own identifier (DOI) so that it can be cited independently in the future. For instructions see: https://journals.plos.org/plosone/s/submission-guidelines#loc-laboratory-protocols . Additionally, PLOS ONE offers an option for publishing peer-reviewed Lab Protocol articles, which describe protocols hosted on protocols.io. Read more information on sharing protocols at https://plos.org/protocols?utm_medium=editorial-email&utm_source=authorletters&utm_campaign=protocols .

We look forward to receiving your revised manuscript.

Kind regards,

Aditya Pawar

Guest Editor

PLOS One

Journal Requirements:

If the reviewer comments include a recommendation to cite specific previously published works, please review and evaluate these publications to determine whether they are relevant and should be cited. There is no requirement to cite these works unless the editor has indicated otherwise.

Please review your reference list to ensure that it is complete and correct. If you have cited papers that have been retracted, please include the rationale for doing so in the manuscript text, or remove these references and replace them with relevant current references. Any changes to the reference list should be mentioned in the rebuttal letter that accompanies your revised manuscript. If you need to cite a retracted article, indicate the article’s retracted status in the References list and also include a citation and full reference for the retraction notice.

Additional Editor Comments:

Kindly address the final comments by Reviewer 2.

[Note: HTML markup is below. Please do not edit.]

Reviewers' comments:

Reviewer's Responses to Questions

**Comments to the Author**

Reviewer #1: All comments have been addressed

Reviewer #2: All comments have been addressed

**********

2. Is the manuscript technically sound, and do the data support the conclusions?

Reviewer #1: Yes

Reviewer #2: Yes

**********

3. Has the statistical analysis been performed appropriately and rigorously?

Reviewer #1: Yes

Reviewer #2: Yes

**********

4. Have the authors made all data underlying the findings in their manuscript fully available?

The PLOS Data policy

Reviewer #1: Yes

Reviewer #2: Yes

**********

5. Is the manuscript presented in an intelligible fashion and written in standard English?

Reviewer #1: Yes

Reviewer #2: Yes

**********

Reviewer #1: Dear Authors,

Thank you for going through revisions and making the paper clearer. I do not have further comments.

Reviewer #2: Thanks for taking care of the minor edits.

With apology concerning the regression model in Table 5 - one more run please:

The first revision regression model included is Cigarette Smoking with a confidence interval of 2.34-25.62 (p-value <0.001*)

in the second revision the regression model included two overlapping variables concerning smoking.

Cigarette Smoking: with a confidence interval of 11.57-73.612 (p-value <0.001*)

Cigarette Accessibility: with a confidence interval of 0.25-229.55 (p-value 0.241)

While Cigarette Smoking is significant in both the first and second revision, the second revision Cigarette Smoking has a much broader CI than the first revision while the overlapping variable Cigarette Accessibility has a massively large CI range 0.25-229.55. Likely co-linearity again.

Please rerun the regression model for Table 5 with either Cigarette Smoking or Cigarette Accessibility alone but not both. One could try each one separately for interest. My hunch is either will be significant and the CI range much narrower.

With appreciation for your work.

**********

what does this mean? ). If published, this will include your full peer review and any attached files.

If you choose “no”, your identity will remain anonymous but your review may still be made public.

**Do you want your identity to be public for this peer review?** For information about this choice, including consent withdrawal, please see our Privacy Policy

Reviewer #1: **Yes:** D.L.I.H.K. PEIRIS

Reviewer #2: No

**********

[NOTE: If reviewer comments were submitted as an attachment file, they will be attached to this email and accessible via the submission site. Please log into your account, locate the manuscript record, and check for the action link "View Attachments". If this link does not appear, there are no attachment files.]

To ensure your figures meet our technical requirements, please review our figure guidelines: https://journals.plos.org/plosone/s/figures

You may also use PLOS’s free figure tool, NAAS, to help you prepare publication quality figures: https://journals.plos.org/plosone/s/figures#loc-tools-for-figure-preparation.

NAAS will assess whether your figures meet our technical requirements by comparing each figure against our figure specifications.

# Author comment: Author response to Decision Letter 3

**DOI:** 10.1371/journal.pone.0344775.r006

15 Dec 2025

Response to reviewers comments

Dear Editor and reviewers,

Thank you very much for the opportunity to revise again with very clear point for improvement. We have re-analysis and revised the paper as suggested. All references have been double checked to ensure that are align with the journal requirement. Please see the new submitted paper and very hope you happy this version.

Thank you,

TK

Editor

Please review your reference list to ensure that it is complete and correct. If you have cited papers that have been retracted, please include the rationale for doing so in the manuscript text, or remove these references and replace them with relevant current references. Any changes to the reference list should be mentioned in the rebuttal letter that accompanies your revised manuscript. If you need to cite a retracted article, indicate the article’s retracted status in the References list and also include a citation and full reference for the retraction notice.

:Thank you very much, we have carefully checked all references before this submission

Reviewer #2: Thanks for taking care of the minor edits.

With apology concerning the regression model in Table 5 - one more run please:

The first revision regression model included is Cigarette Smoking with a confidence interval of 2.34-25.62 (p-value <0.001*)

in the second revision the regression model included two overlapping variables concerning smoking.

Cigarette Smoking: with a confidence interval of 11.57-73.612 (p-value <0.001*)

Cigarette Accessibility: with a confidence interval of 0.25-229.55 (p-value 0.241)

While Cigarette Smoking is significant in both the first and second revision, the second revision Cigarette Smoking has a much broader CI than the first revision while the overlapping variable Cigarette Accessibility has a massively large CI range 0.25-229.55. Likely co-linearity again.

Please rerun the regression model for Table 5 with either Cigarette Smoking or Cigarette Accessibility alone but not both. One could try each one separately for interest. My hunch is either will be significant and the CI range much narrower.

: We have re-run a final model again with removed cigarette accessibility out of the final model. The range of CI of cigarette smoking was narrower than 2nd revised. All information has been revised and improved accordingly. Please see in page.2, lines 3-13, page 22 lines 3-15, and table 5 in page 23-24.

Thank you,

TK

Tawatchai Apidehckul

Attachment Submitted filename: MA_Reviewers_commenst_auresp_3.pdf

# Peer review: Decision Letter 3

**DOI:** 10.1371/journal.pone.0344775.r007

**Authors:** Ghaffar Ali

3 Feb 2026

Dear Dr. Apidechkul,

Please address the final minor comments from Reviewer 2.

Please submit your revised manuscript by Mar 18 2026 11:59PM. If you will need more time than this to complete your revisions, please reply to this message or contact the journal office at plosone@plos.org . When you're ready to submit your revision, log on to https://www.editorialmanager.com/pone/ and select the 'Submissions Needing Revision' folder to locate your manuscript file.

Please include the following items when submitting your revised manuscript:

- A letter that responds to each point raised by the academic editor and reviewer(s). You should upload this letter as a separate file labeled 'Response to Reviewers'.
- A marked-up copy of your manuscript that highlights changes made to the original version. You should upload this as a separate file labeled 'Revised Manuscript with Track Changes'.
- An unmarked version of your revised paper without tracked changes. You should upload this as a separate file labeled 'Manuscript'.

If applicable, we recommend that you deposit your laboratory protocols in protocols.io to enhance the reproducibility of your results. Protocols.io assigns your protocol its own identifier (DOI) so that it can be cited independently in the future. For instructions see: https://journals.plos.org/plosone/s/submission-guidelines#loc-laboratory-protocols . Additionally, PLOS ONE offers an option for publishing peer-reviewed Lab Protocol articles, which describe protocols hosted on protocols.io. Read more information on sharing protocols at https://plos.org/protocols?utm_medium=editorial-email&utm_source=authorletters&utm_campaign=protocols .

We look forward to receiving your revised manuscript.

Kind regards,

Alejandro Torrado Pacheco, PhD

Associate Editor

PLOS One

on behalf of

Ghaffar Ali, PhD

Academic Editor

PLOS One

Journal Requirements:

If the reviewer comments include a recommendation to cite specific previously published works, please review and evaluate these publications to determine whether they are relevant and should be cited. There is no requirement to cite these works unless the editor has indicated otherwise.

Please review your reference list to ensure that it is complete and correct. If you have cited papers that have been retracted, please include the rationale for doing so in the manuscript text, or remove these references and replace them with relevant current references. Any changes to the reference list should be mentioned in the rebuttal letter that accompanies your revised manuscript. If you need to cite a retracted article, indicate the article’s retracted status in the References list and also include a citation and full reference for the retraction notice.

[Note: HTML markup is below. Please do not edit.]

Reviewers' comments:

Reviewer's Responses to Questions

**Comments to the Author**

Reviewer #1: All comments have been addressed

Reviewer #2: All comments have been addressed

**********

2. Is the manuscript technically sound, and do the data support the conclusions?

Reviewer #1: Yes

Reviewer #2: Yes

**********

3. Has the statistical analysis been performed appropriately and rigorously?

Reviewer #1: N/A

Reviewer #2: Yes

**********

4. Have the authors made all data underlying the findings in their manuscript fully available?

The PLOS Data policy

Reviewer #1: Yes

Reviewer #2: Yes

**********

5. Is the manuscript presented in an intelligible fashion and written in standard English?

Reviewer #1: Yes

Reviewer #2: Yes

**********

Reviewer #1: Dear Authors,

I do not have further comments to add at this moment. However, it is appreciated that you have invested time to recheck the regression models, and the formatting of the paper, including the in-text citations and the references.

Thank you.

Reviewer #2: 1.27.2026 Comments on third revision.

Thank you again for re-running the model for Table 5 with one smoking variable. It is fine. I appreciate your long patience for all of my requests.

In closing I would like to suggest a substantive comment for Table 4 and a minor edit in introducing Table 5. Fielder's choice (meaning your choice whether to do this) ...

%%%%%%%%%%%%%%%%%%%%%%%%

** PAGE 17 concerning Table 4 results:

Looking at Table 4’s Univariate analysis on pages 20-21 I notice a pattern when comparing odds ratios between ‘friend culture’ and ‘family culture’ regarding Smoking, Alcohol use, Cannabis etc. use, MA use, and MA pressure. All ORs were significant, however the Friend ORs were systematically higher than the Family ORs for each of these:

Smoking ORs: Friends 5.04, Family 2.68

Alcohol use ORs: Friends 3.20, Family 3.05

Cannabis etc use ORs: Friends 4.74, Family 1.98

MA use ORs: Friends 11.26, Family 2.59

MA use pressure ORs: Friends 15.77, Family 4.24

So in every case ORs on the impact of Friends was higher than ORs on the impact of Family, sometimes much higher.

Given this, on page 17 after the last paragraph (17.14) you might add a comment such as:

“In the univariate analysis (Table 4) it is notable that the influence of friends exceeded that of the influence of family in the odds ratios in each category regarding Smoking, Alcohol use, Cannabis etc. use, MA use, and pressure to use MA – in some cases the Friends ORs were more than two- to three-fold higher than Family ORs . This has implications for prevention and intervention concerning social networks.”

[This comment also or instead could be placed somewhere in the Discussion section.]

%%%%%%%%%%%%%%%%%

** PAGE 22 introducing Table 5:

Page 22.4 model. => model (Table 5).

%%%%%%%%%%%%%%%%%%

I do not need to see the paper again. Thank you for doing this research. May it have fruitful effects for interventions for Lahu youth. Blessings!

**********

what does this mean? ). If published, this will include your full peer review and any attached files.

If you choose “no”, your identity will remain anonymous but your review may still be made public.

**Do you want your identity to be public for this peer review?** For information about this choice, including consent withdrawal, please see our Privacy Policy

Reviewer #1: No

Reviewer #2: **Yes:** Kirk C Allison

**********

[NOTE: If reviewer comments were submitted as an attachment file, they will be attached to this email and accessible via the submission site. Please log into your account, locate the manuscript record, and check for the action link "View Attachments". If this link does not appear, there are no attachment files.]

To ensure your figures meet our technical requirements, please review our figure guidelines: https://journals.plos.org/plosone/s/figures

You may also use PLOS’s free figure tool, NAAS, to help you prepare publication quality figures: https://journals.plos.org/plosone/s/figures#loc-tools-for-figure-preparation.

NAAS will assess whether your figures meet our technical requirements by comparing each figure against our figure specifications.

# Author comment: Author response to Decision Letter 4

**DOI:** 10.1371/journal.pone.0344775.r008

3 Feb 2026

Respond to reviewers’ comments

Dear Editor,

Thank you so much for the opportunity to revise this paper based on the valuable comments from the reviewers. We have carefully revised and do hope that you happy with this version.

Thank you,

TK

Please use the space provided to explain your answers to the questions above. You may also include additional comments for the author, including concerns about dual publication, research ethics, or publication ethics. (Please upload your review as an attachment if it exceeds 20,000 characters)

Reviewer #1: Dear Authors,

I do not have further comments to add at this moment. However, it is appreciated that you have invested time to recheck the regression models, and the formatting of the paper, including the in-text citations and the references.

Thank you.

: Thank you very much

Reviewer #2: 1.27.2026 Comments on third revision.

Thank you again for re-running the model for Table 5 with one smoking variable. It is fine. I appreciate your long patience for all of my requests.

In closing I would like to suggest a substantive comment for Table 4 and a minor edit in introducing Table 5. Fielder's choice (meaning your choice whether to do this) ...

%%%%%%%%%%%%%%%%%%%%%%%%

** PAGE 17 concerning Table 4 results:

Looking at Table 4’s Univariate analysis on pages 20-21 I notice a pattern when comparing odds ratios between ‘friend culture’ and ‘family culture’ regarding Smoking, Alcohol use, Cannabis etc. use, MA use, and MA pressure. All ORs were significant, however the Friend ORs were systematically higher than the Family ORs for each of these:

Smoking ORs: Friends 5.04, Family 2.68

Alcohol use ORs: Friends 3.20, Family 3.05

Cannabis etc use ORs: Friends 4.74, Family 1.98

MA use ORs: Friends 11.26, Family 2.59

MA use pressure ORs: Friends 15.77, Family 4.24

So in every case ORs on the impact of Friends was higher than ORs on the impact of Family, sometimes much higher.

Given this, on page 17 after the last paragraph (17.14) you might add a comment such as:

“In the univariate analysis (Table 4) it is notable that the influence of friends exceeded that of the influence of family in the odds ratios in each category regarding Smoking, Alcohol use, Cannabis etc. use, MA use, and pressure to use MA – in some cases the Friends ORs were more than two- to three-fold higher than Family ORs . This has implications for prevention and intervention concerning social networks.”

: Thank you so much for your kind support. It’s added, please see page 17, line 15-19.

[This comment also or instead could be placed somewhere in the Discussion section.]

%%%%%%%%%%%%%%%%%

** PAGE 22 introducing Table 5:

Page 22.4 model. => model (Table 5).

: Thank you, it is added, page 22, line 4.

%%%%%%%%%%%%%%%%%%

I do not need to see the paper again. Thank you for doing this research. May it have fruitful effects for interventions for Lahu youth. Blessings!

: Thank you so much

TK

# Editor report: Decision Letter 4

**DOI:** 10.1371/journal.pone.0344775.r009

**Authors:** Ghaffar Ali

25 Feb 2026

Factors influencing methamphetamine use among Lahu hill tribe youth in Chiang Rai, Thailand: A case-control study

PONE-D-25-27632R4

Dear Dr. Apidechkul,

We’re pleased to inform you that your manuscript has been judged scientifically suitable for publication and will be formally accepted for publication once it meets all outstanding technical requirements.

Within one week, you’ll receive an e-mail detailing the required amendments. When these have been addressed, you’ll receive a formal acceptance letter and your manuscript will be scheduled for publication.

An invoice will be generated when your article is formally accepted. Please note, if your institution has a publishing partnership with PLOS and your article meets the relevant criteria, all or part of your publication costs will be covered. Please make sure your user information is up-to-date by logging into Editorial Manager at Editorial Manager®  and clicking the ‘Update My Information' link at the top of the page. For questions related to billing, please contact billing support .

If your institution or institutions have a press office, please notify them about your upcoming paper to help maximize its impact. If they’ll be preparing press materials, please inform our press team as soon as possible -- no later than 48 hours after receiving the formal acceptance. Your manuscript will remain under strict press embargo until 2 pm Eastern Time on the date of publication. For more information, please contact onepress@plos.org.

Kind regards,

Ghaffar Ali, PhD

Academic Editor

PLOS One

Additional Editor Comments (optional):

Reviewers' comments:

# Editor report: Acceptance letter

**DOI:** 10.1371/journal.pone.0344775.r010

**Authors:** Ghaffar Ali

PONE-D-25-27632R4

PLOS One

Dear Dr. Apidechkul,

I'm pleased to inform you that your manuscript has been deemed suitable for publication in PLOS One. Congratulations! Your manuscript is now being handed over to our production team.

At this stage, our production department will prepare your paper for publication. This includes ensuring the following:

* All references, tables, and figures are properly cited

* All relevant supporting information is included in the manuscript submission,

* There are no issues that prevent the paper from being properly typeset

You will receive further instructions from the production team, including instructions on how to review your proof when it is ready. Please keep in mind that we are working through a large volume of accepted articles, so please give us a few days to review your paper and let you know the next and final steps.

Lastly, if your institution or institutions have a press office, please let them know about your upcoming paper now to help maximize its impact. If they'll be preparing press materials, please inform our press team within the next 48 hours. Your manuscript will remain under strict press embargo until 2 pm Eastern Time on the date of publication. For more information, please contact onepress@plos.org.

You will receive an invoice from PLOS for your publication fee after your manuscript has reached the completed accept phase. If you receive an email requesting payment before acceptance or for any other service, this may be a phishing scheme. Learn how to identify phishing emails and protect your accounts at https://explore.plos.org/phishing.

If we can help with anything else, please email us at customercare@plos.org.

Thank you for submitting your work to PLOS ONE and supporting open access.

Kind regards,

PLOS ONE Editorial Office Staff

on behalf of

Prof. Ghaffar Ali

Academic Editor

PLOS One
